# Supplementary figures and images for: The Ca2+-activated cation channel TRPM4 is a positive regulator of pressure overload-induced cardiac hypertrophy (part 2 of 2)
Source: eLife. 2021 Jun 30;10:e66582. doi: 10.7554/eLife.66582 (PMC8245133; doi:10.7554/eLife.66582)

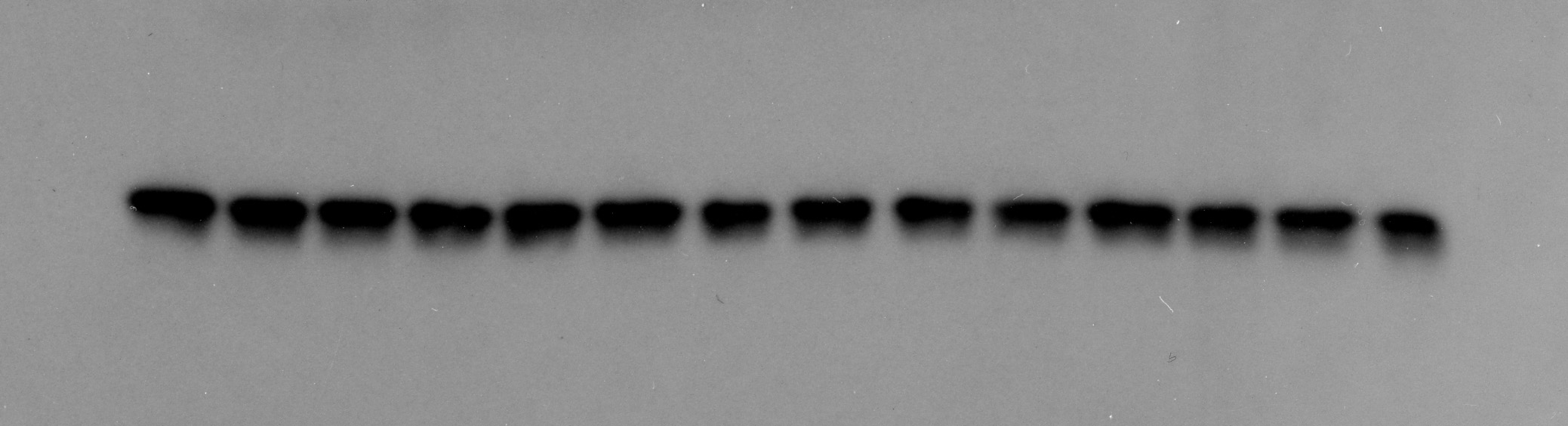

Supplement: Source data 1. [file elife-66582-data1.zip › Blots and Blot Figs/Blots/WT Nu/WT Histone H2B5.jpg]

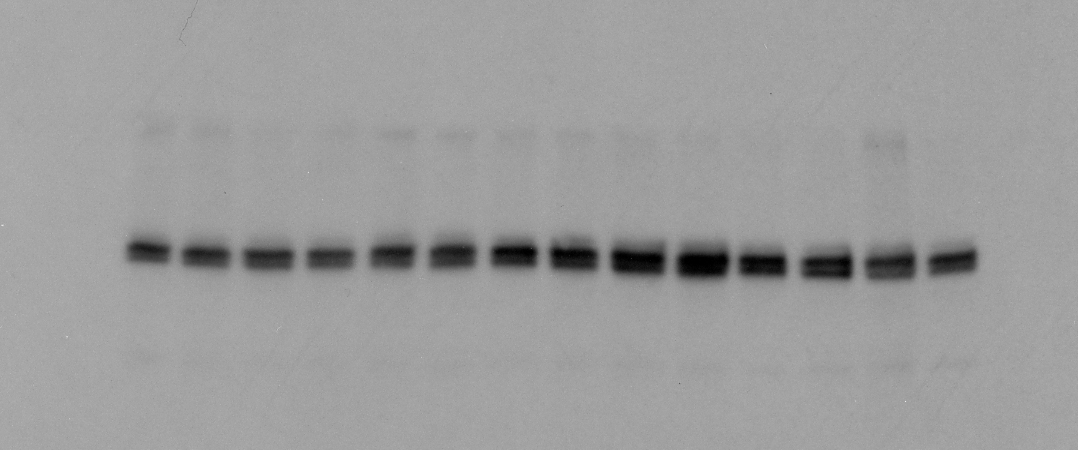

Supplement: Source data 1. [file elife-66582-data1.zip › Blots and Blot Figs/Blots/WT Nu/WT Nu CaMK2.jpg]

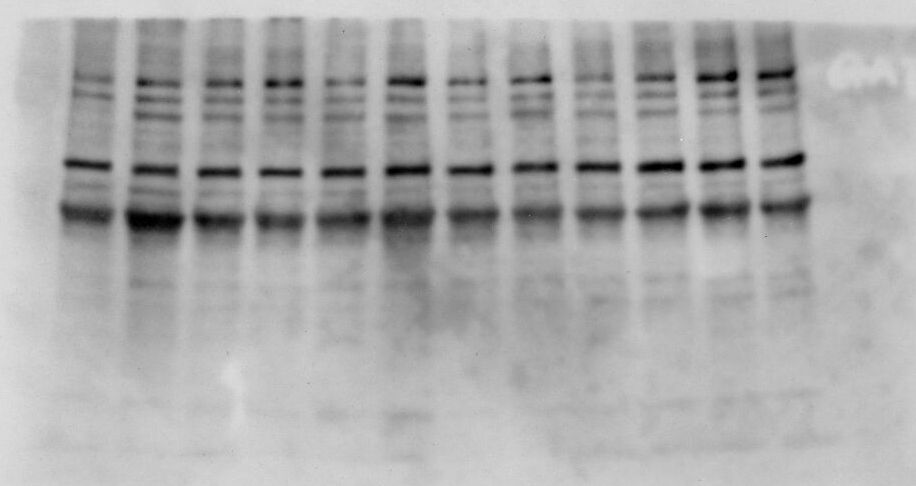

Supplement: Source data 1. [file elife-66582-data1.zip › Blots and Blot Figs/Blots/WT Nu/WT Nu GATA4.jpg]

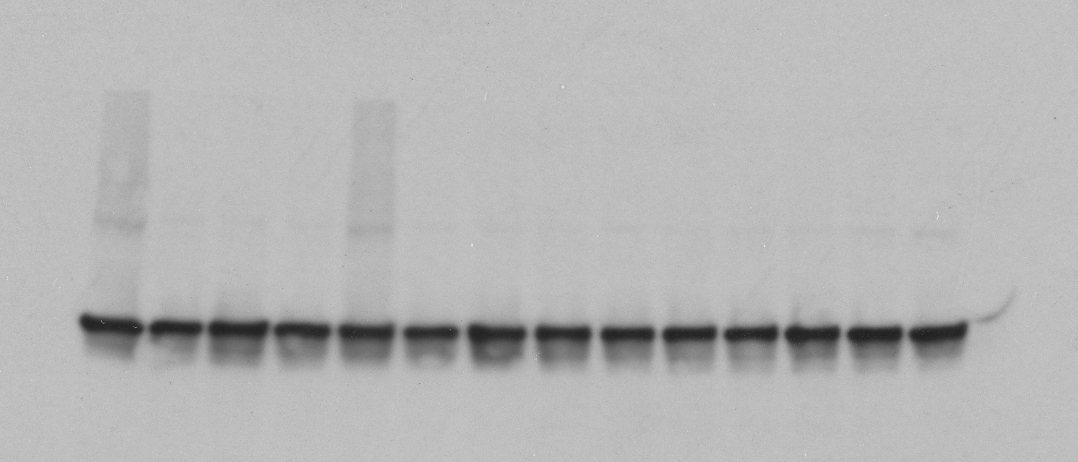

Supplement: Source data 1. [file elife-66582-data1.zip › Blots and Blot Figs/Blots/WT Nu/WT Nu GSK3b.jpg]

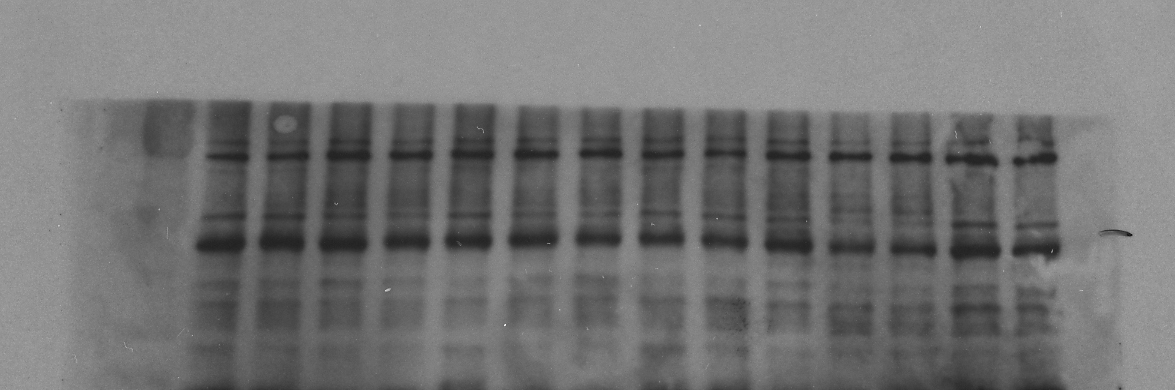

Supplement: Source data 1. [file elife-66582-data1.zip › Blots and Blot Figs/Blots/WT Nu/WT Nu HDAC4.jpg]

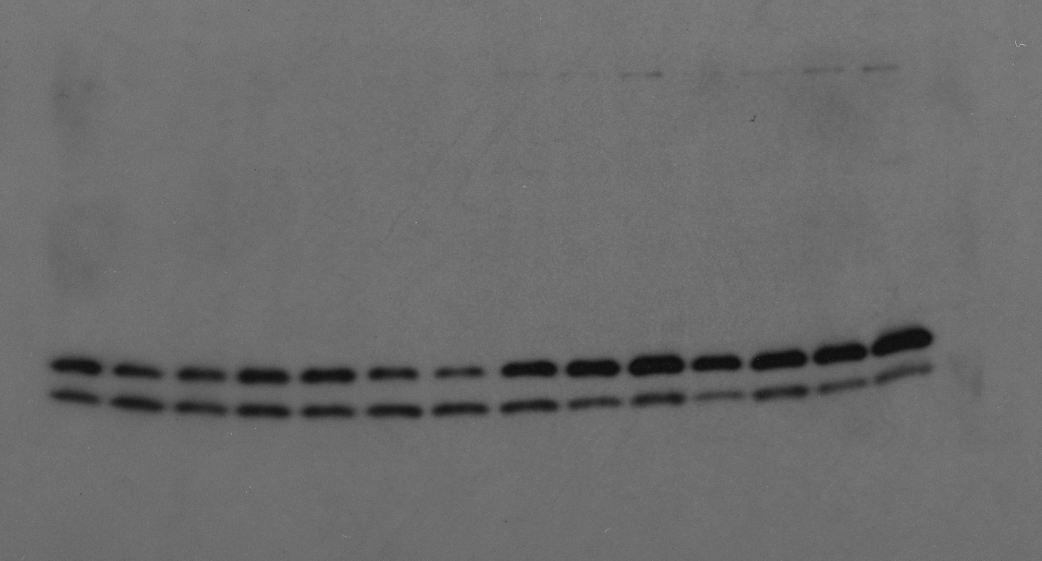

Supplement: Source data 1. [file elife-66582-data1.zip › Blots and Blot Figs/Blots/WT Nu/WT Nu MEF2.jpg]

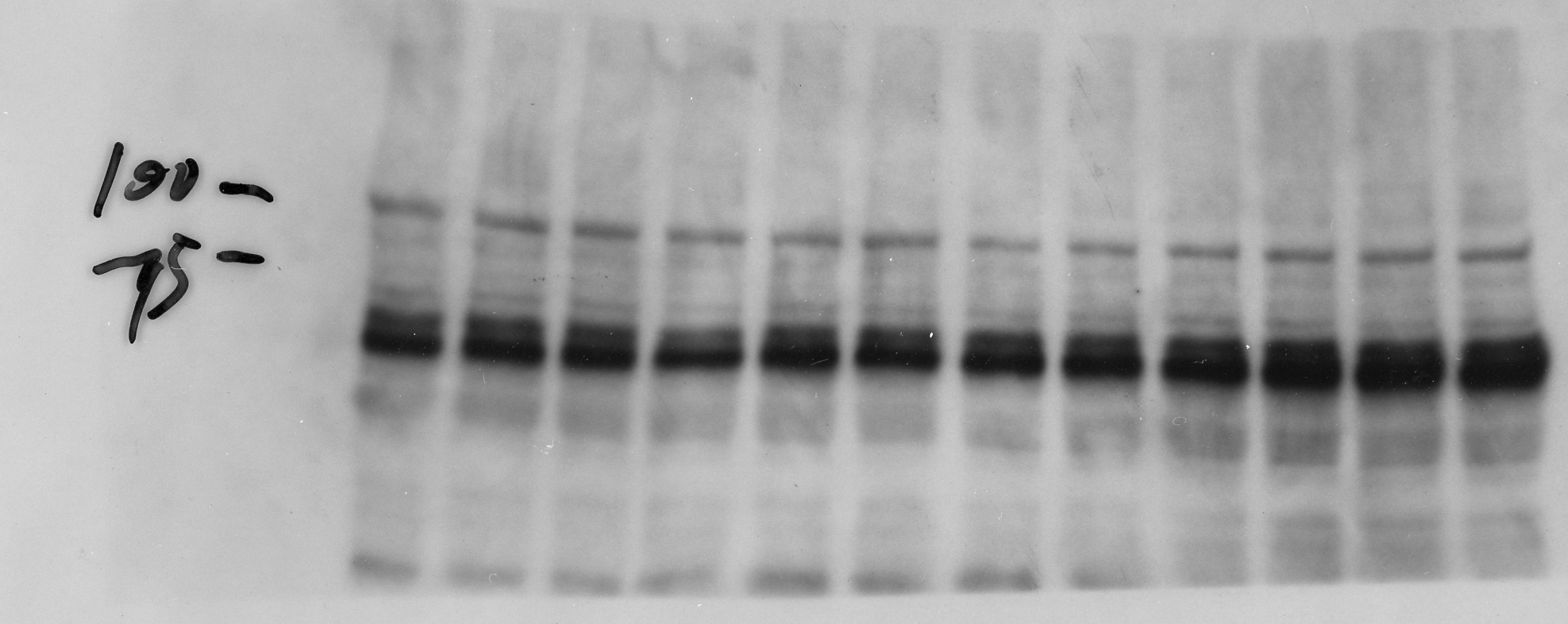

Supplement: Source data 1. [file elife-66582-data1.zip › Blots and Blot Figs/Blots/WT Nu/WT Nu NFAT.jpg]

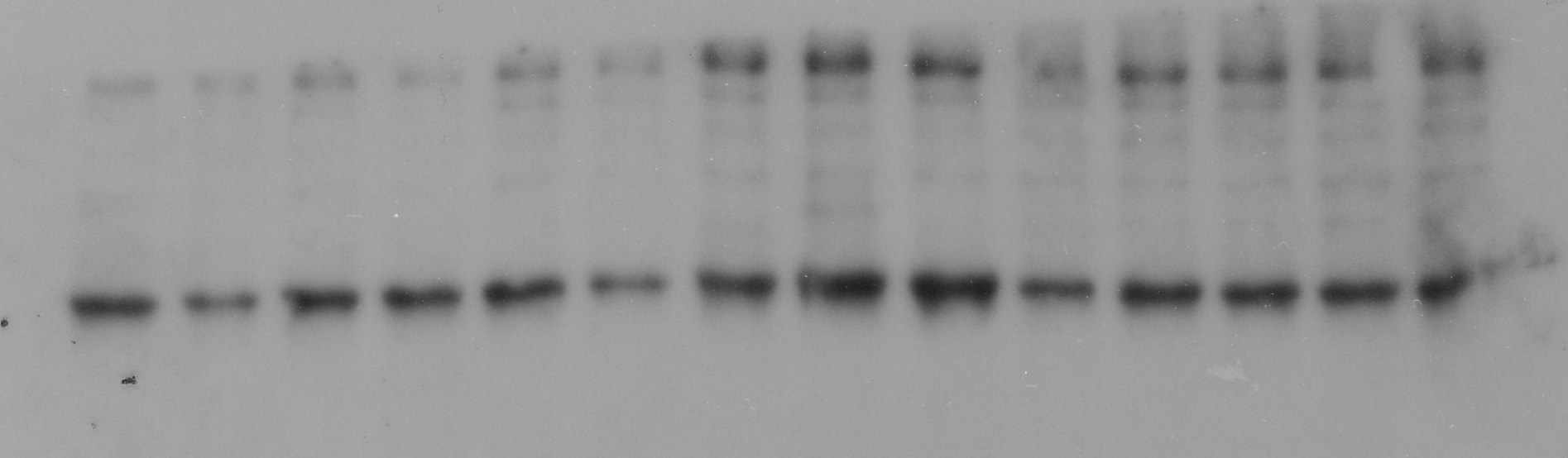

Supplement: Source data 1. [file elife-66582-data1.zip › Blots and Blot Figs/Blots/WT Nu/WT Nu p-CaMK2.jpg]

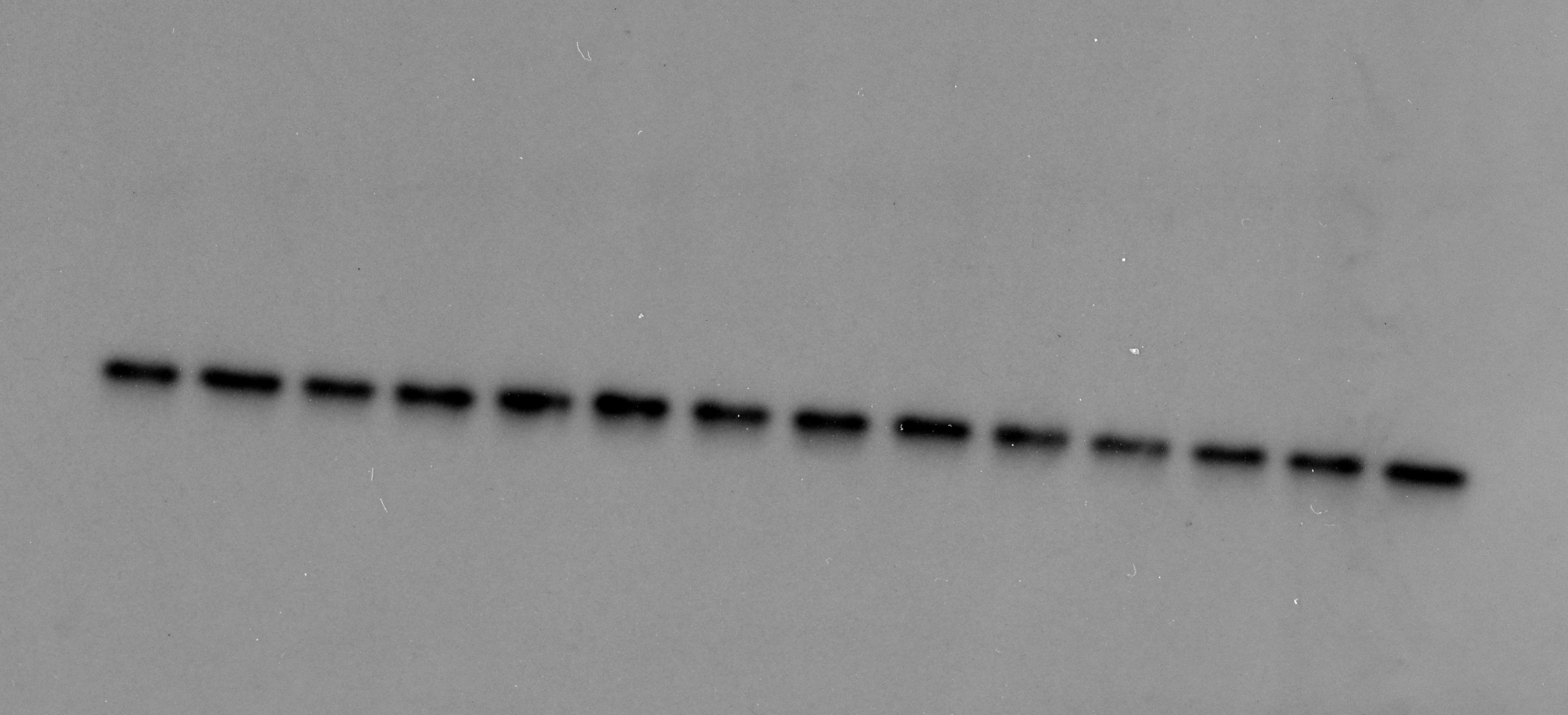

Supplement: Source data 1. [file elife-66582-data1.zip › Blots and Blot Figs/Blots/WT Nu/WT Nu p-GSK3b.jpg]

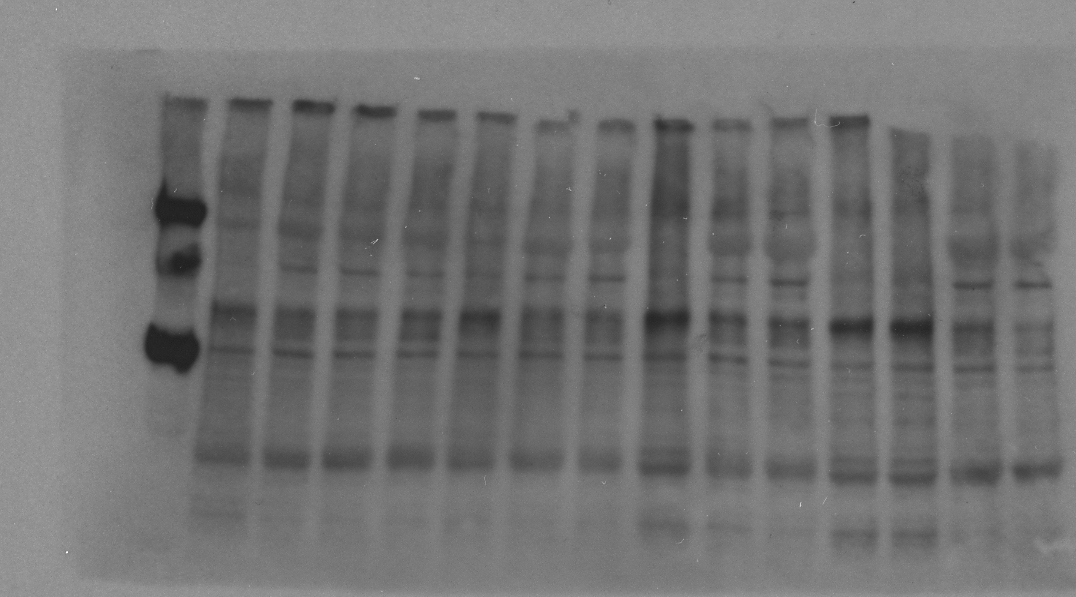

Supplement: Source data 1. [file elife-66582-data1.zip › Blots and Blot Figs/Blots/WT Nu/WT Nu p-HDAC4.jpg]

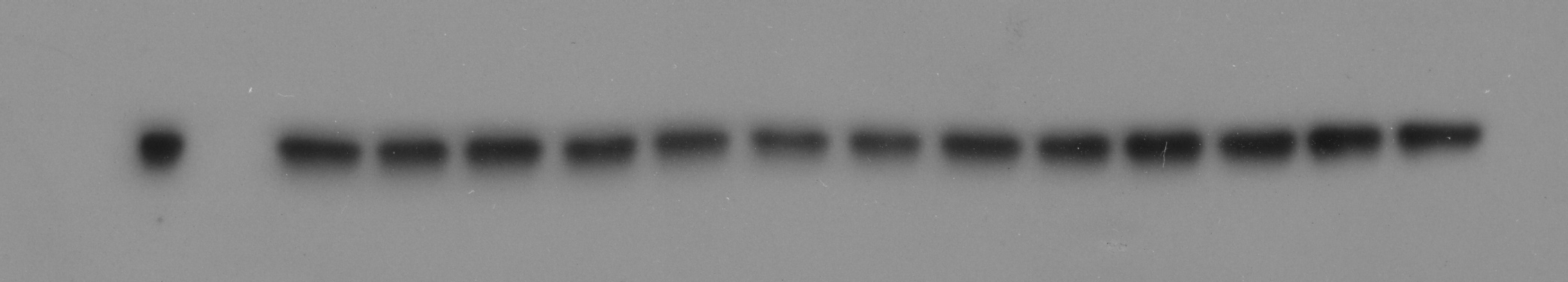

Supplement: Source data 1. [file elife-66582-data1.zip › Blots and Blot Figs/Blots/WT TRPM4/CM 14days GAPDH.jpg]

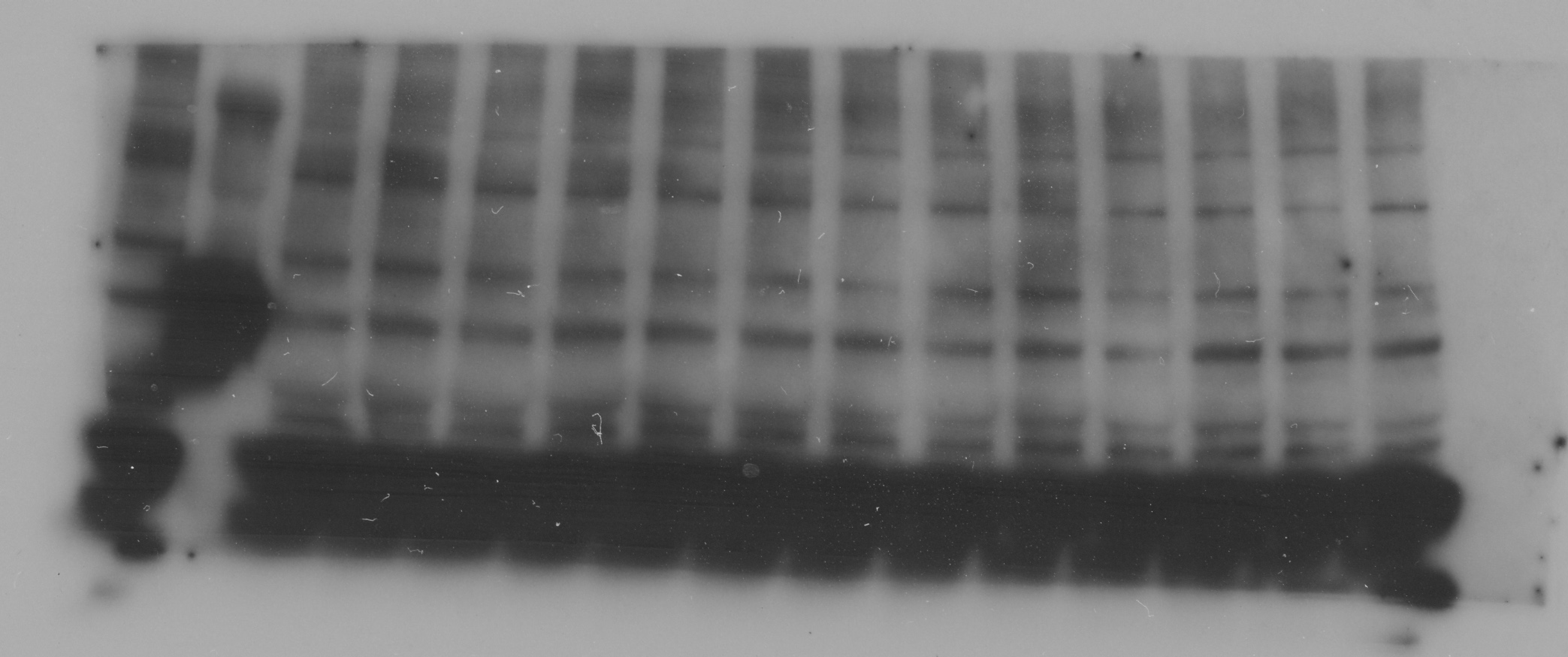

Supplement: Source data 1. [file elife-66582-data1.zip › Blots and Blot Figs/Blots/WT TRPM4/CM 14days TRPM4.jpg]

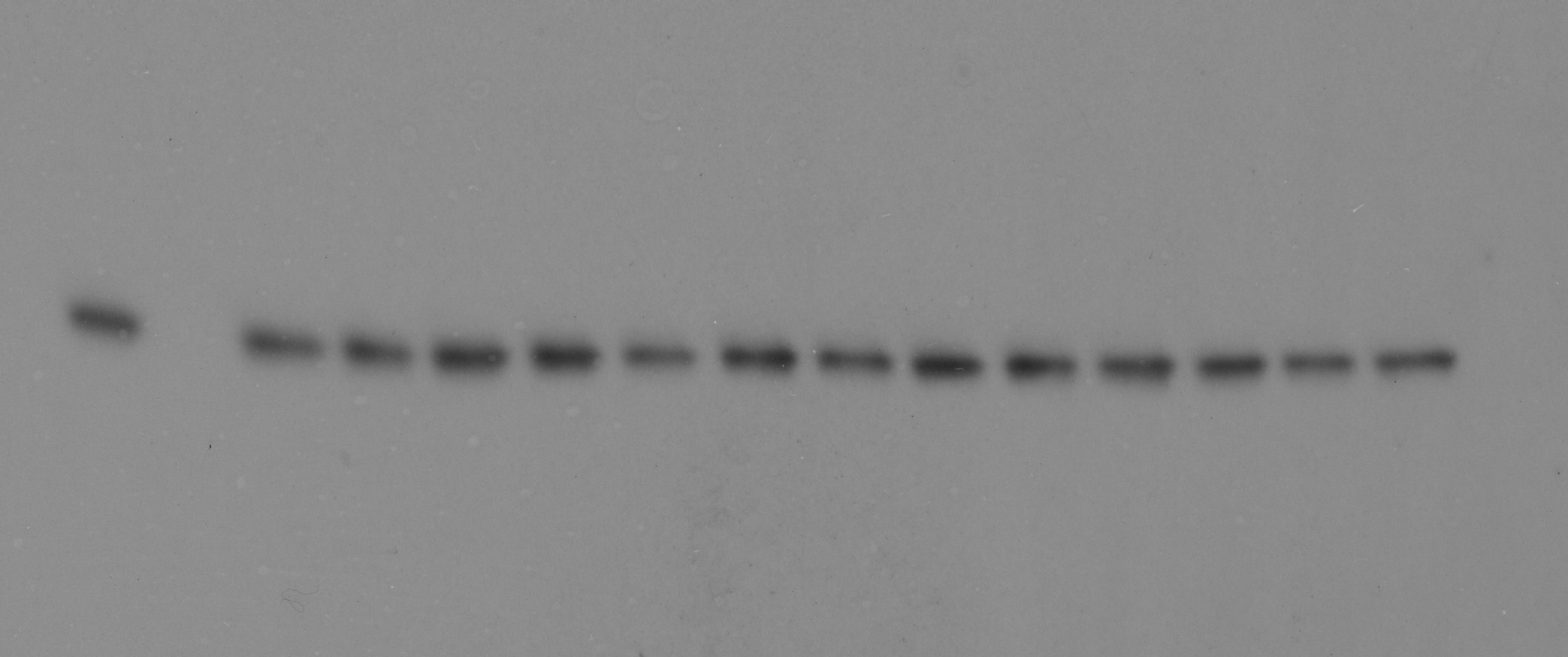

Supplement: Source data 1. [file elife-66582-data1.zip › Blots and Blot Figs/Blots/WT TRPM4/CM 2days GAPDH.jpg]

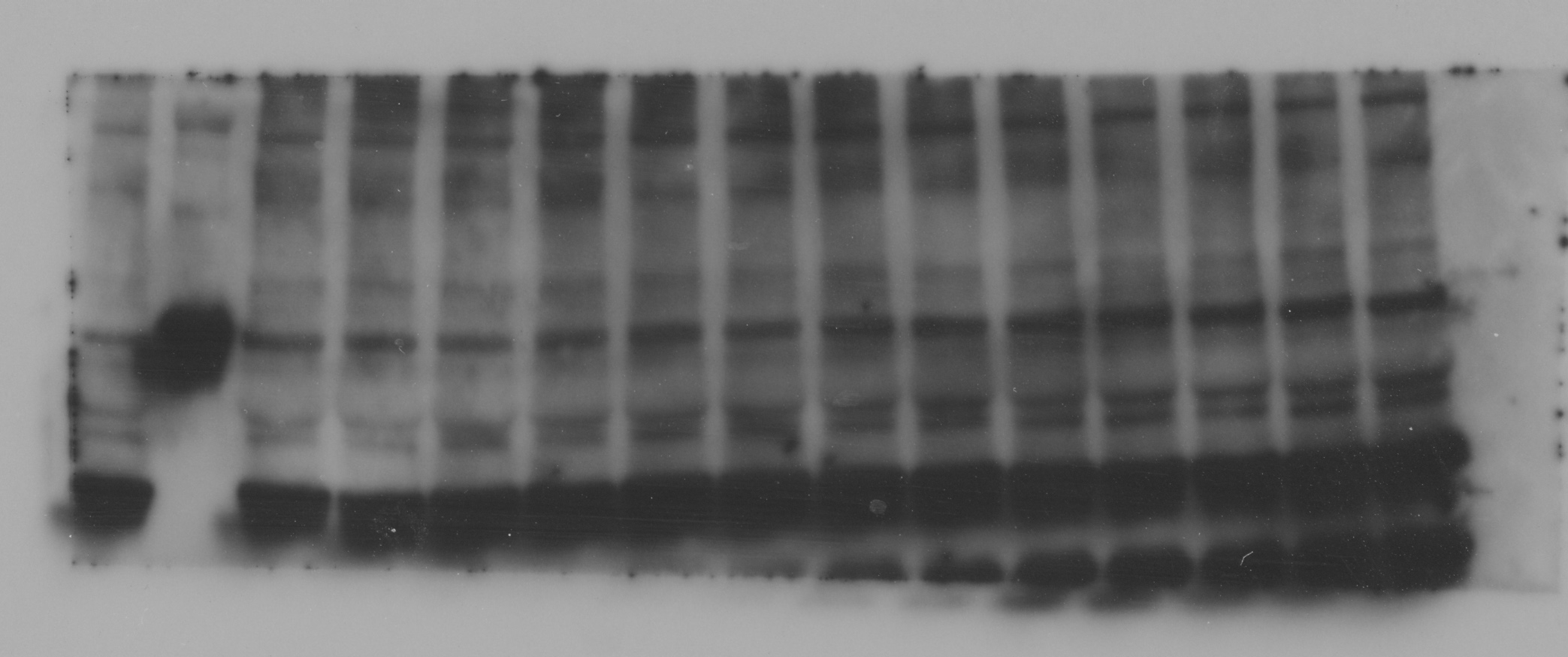

Supplement: Source data 1. [file elife-66582-data1.zip › Blots and Blot Figs/Blots/WT TRPM4/CM 2days TRPM4.jpg]

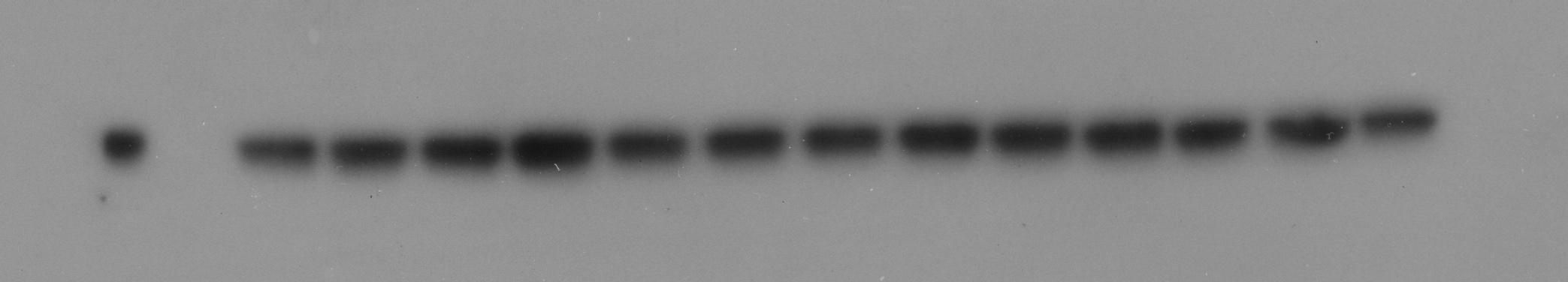

Supplement: Source data 1. [file elife-66582-data1.zip › Blots and Blot Figs/Blots/WT TRPM4/LV 14days GAPDH.jpg]

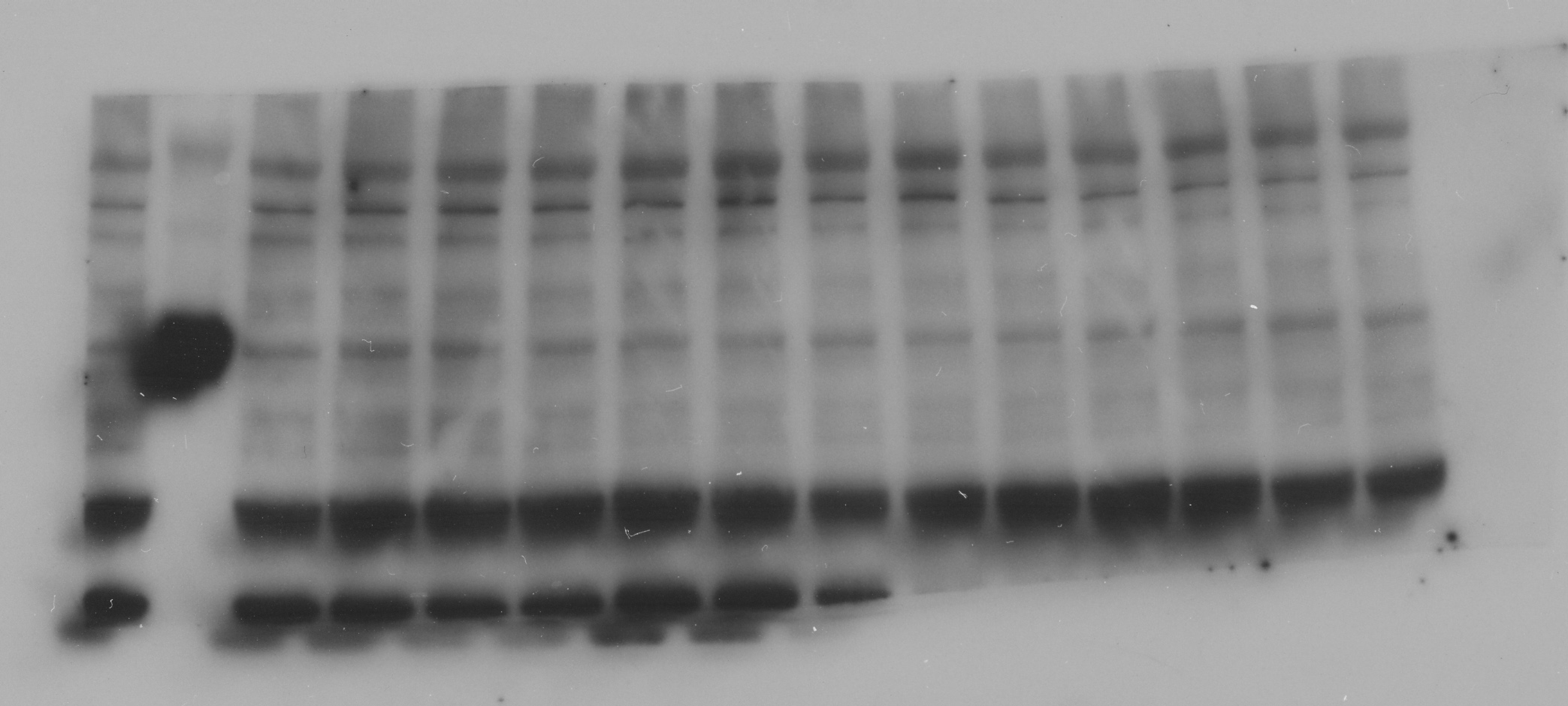

Supplement: Source data 1. [file elife-66582-data1.zip › Blots and Blot Figs/Blots/WT TRPM4/LV 14days TRPM4.jpg]

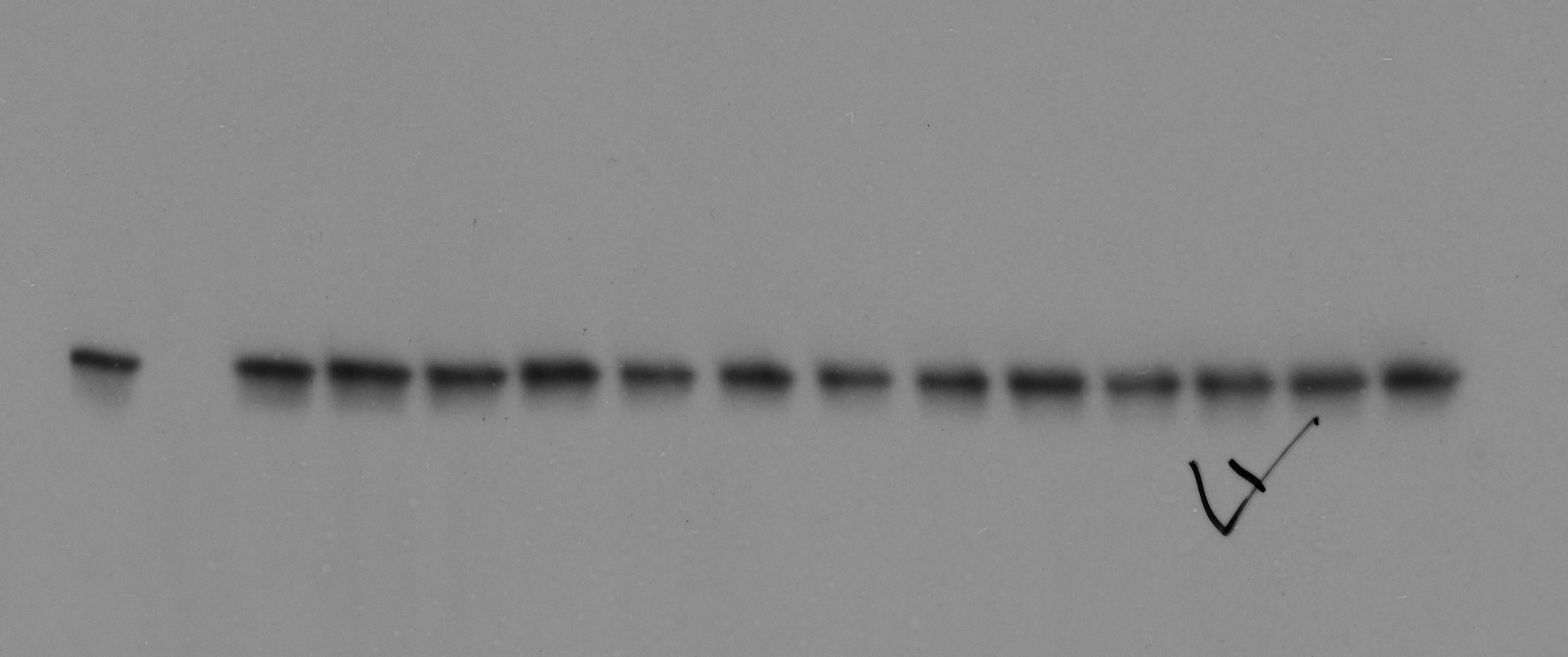

Supplement: Source data 1. [file elife-66582-data1.zip › Blots and Blot Figs/Blots/WT TRPM4/LV 2days GAPDH.jpg]

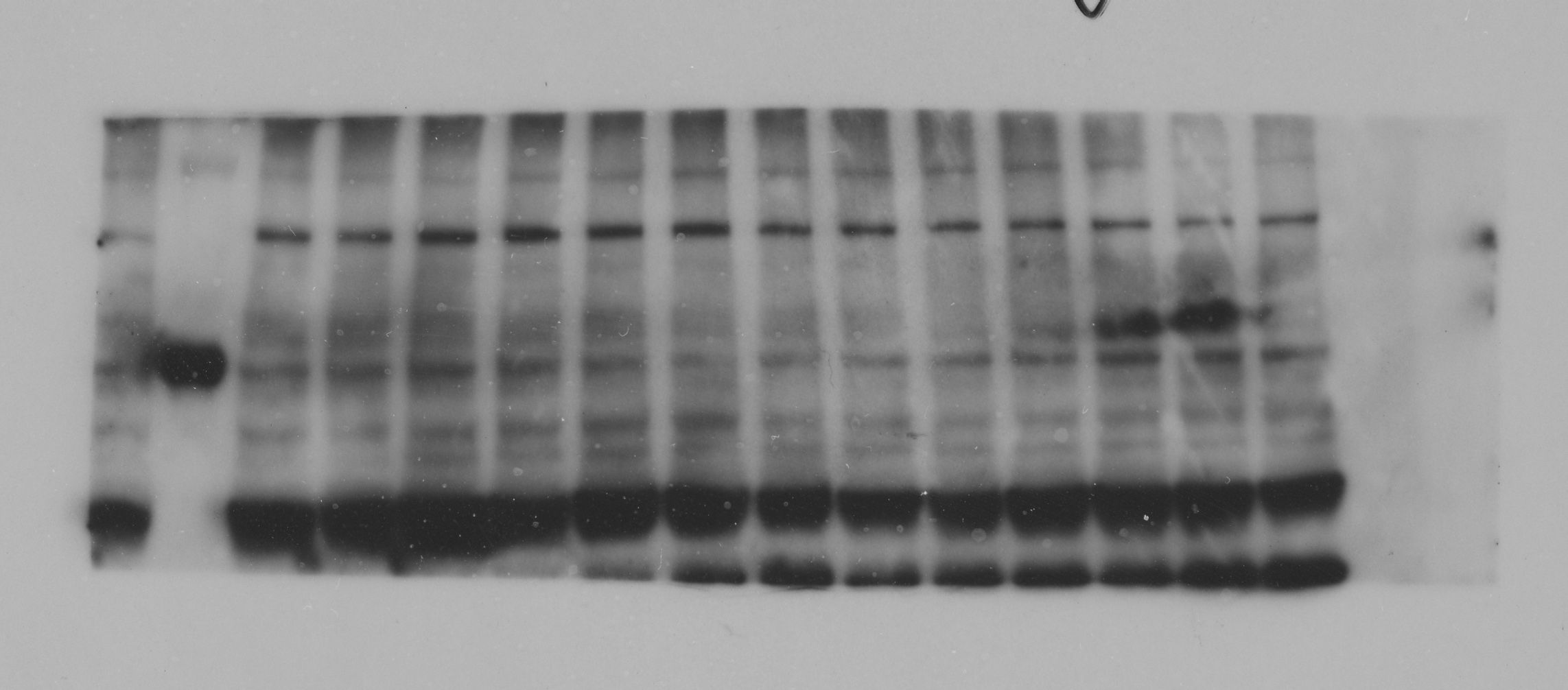

Supplement: Source data 1. [file elife-66582-data1.zip › Blots and Blot Figs/Blots/WT TRPM4/LV 2days TRPM4.jpg]
