# Supplementary figures and images for: The Ca2+-activated cation channel TRPM4 is a positive regulator of pressure overload-induced cardiac hypertrophy (part 1 of 2)
Source: eLife. 2021 Jun 30;10:e66582. doi: 10.7554/eLife.66582 (PMC8245133; doi:10.7554/eLife.66582)

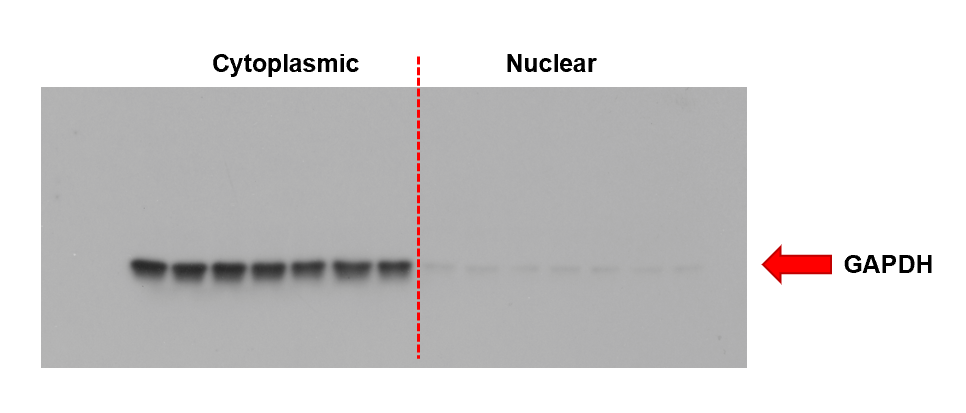

Supplement: Source data 1. [file elife-66582-data1.zip › Blots and Blot Figs/Blot Figs/Cyto_Nu fraction/GAPDH Fig.tif]

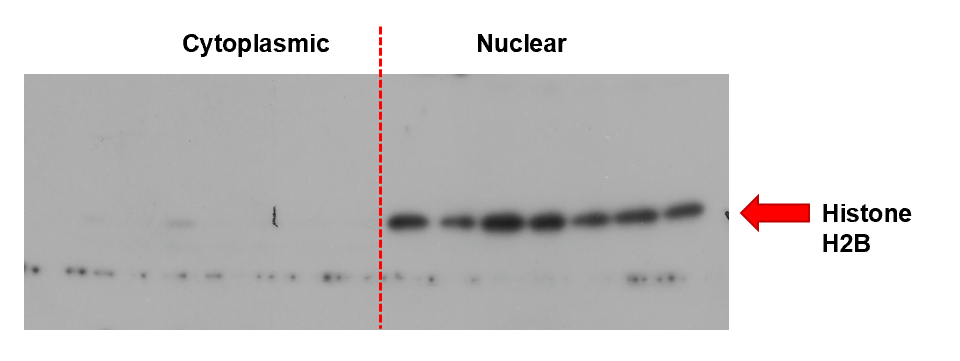

Supplement: Source data 1. [file elife-66582-data1.zip › Blots and Blot Figs/Blot Figs/Cyto_Nu fraction/Histone H2B Fig.tif]

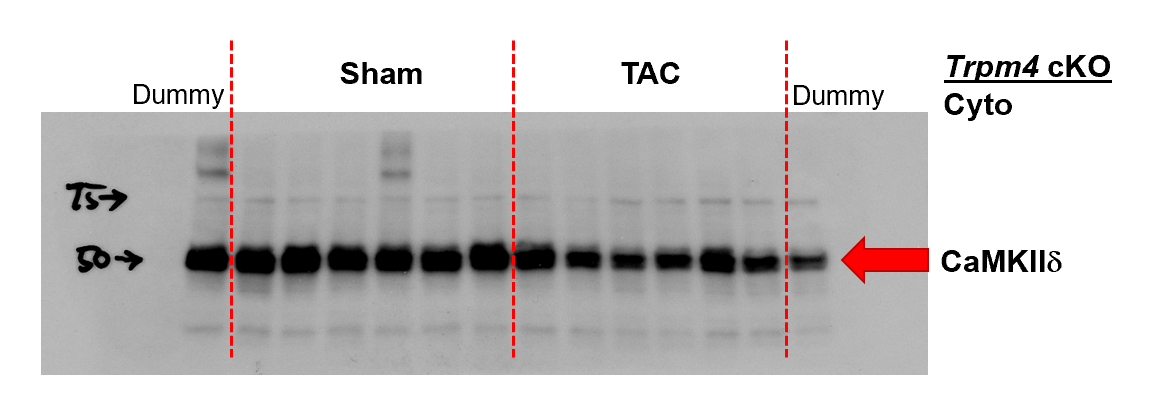

Supplement: Source data 1. [file elife-66582-data1.zip › Blots and Blot Figs/Blot Figs/KO Cyto/KO Cyto CaMK2 Fig.tif]

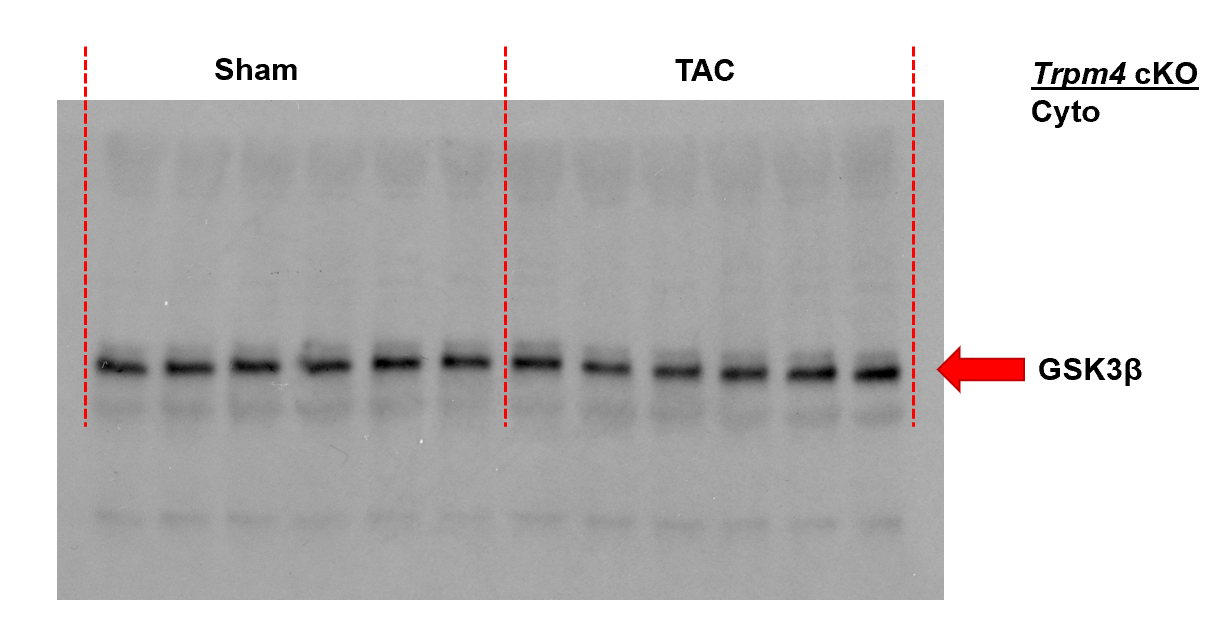

Supplement: Source data 1. [file elife-66582-data1.zip › Blots and Blot Figs/Blot Figs/KO Cyto/KO Cyto GSK3b Fig.tif]

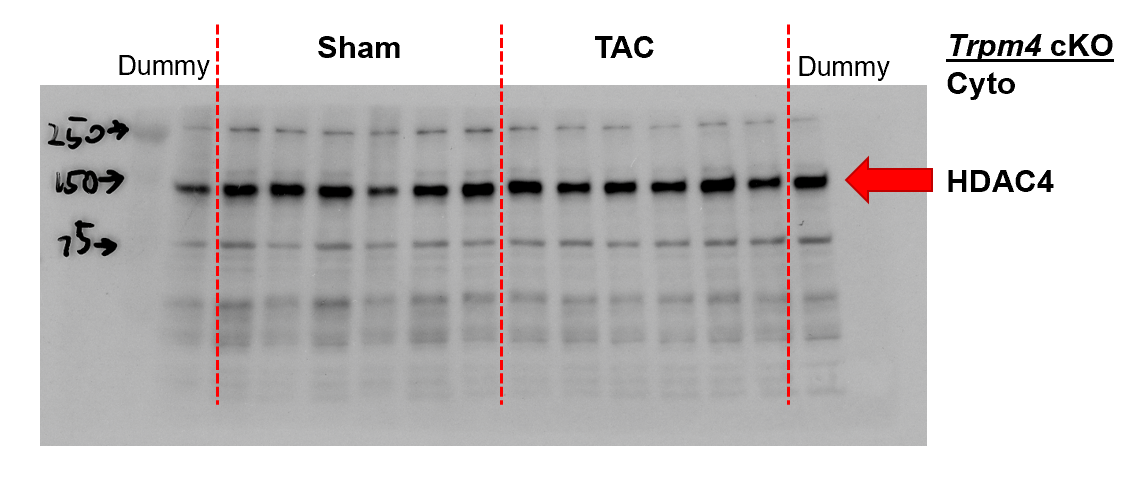

Supplement: Source data 1. [file elife-66582-data1.zip › Blots and Blot Figs/Blot Figs/KO Cyto/KO Cyto HDAC4 Fig.tif]

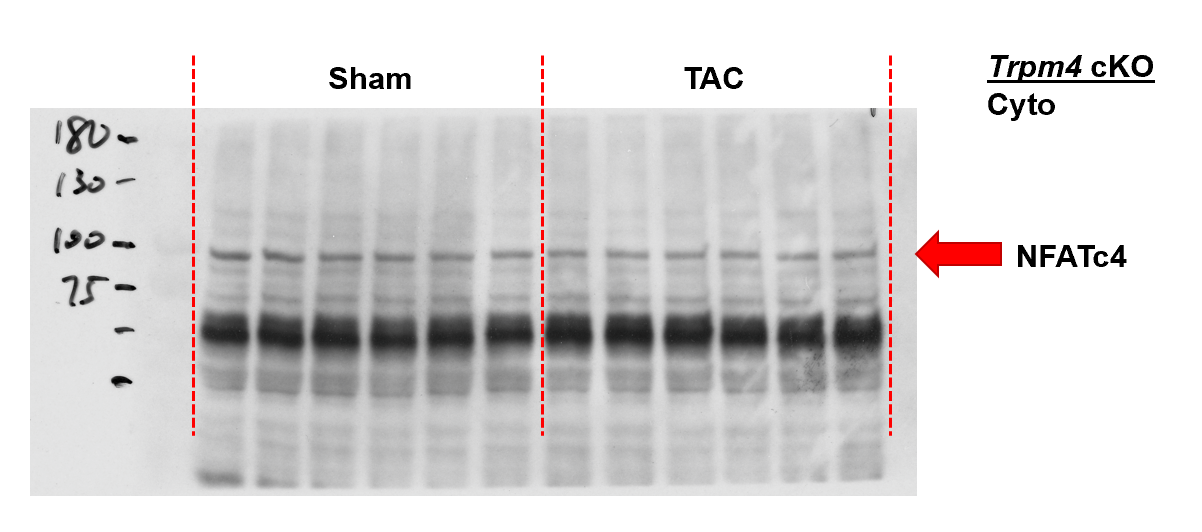

Supplement: Source data 1. [file elife-66582-data1.zip › Blots and Blot Figs/Blot Figs/KO Cyto/KO Cyto NFAT Fig.tif]

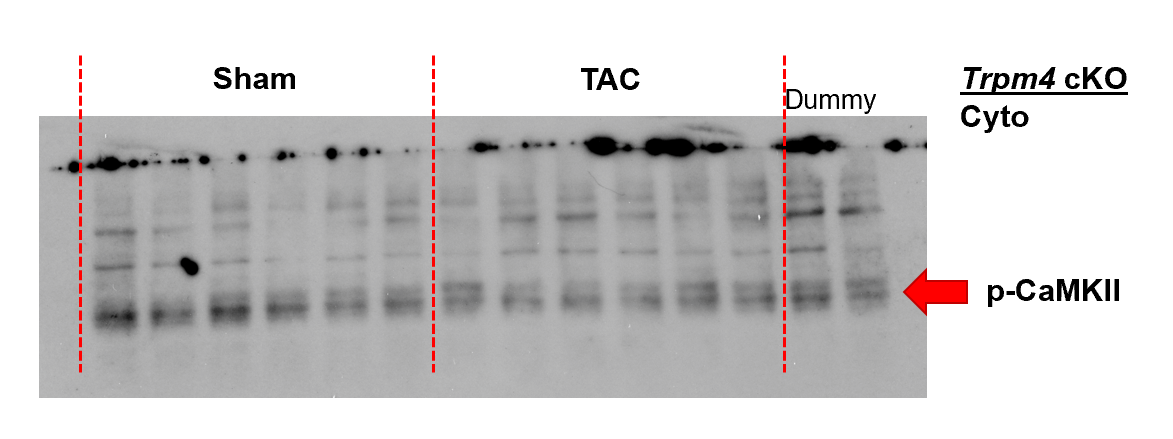

Supplement: Source data 1. [file elife-66582-data1.zip › Blots and Blot Figs/Blot Figs/KO Cyto/KO Cyto p-CaMK2 Fig.tif]

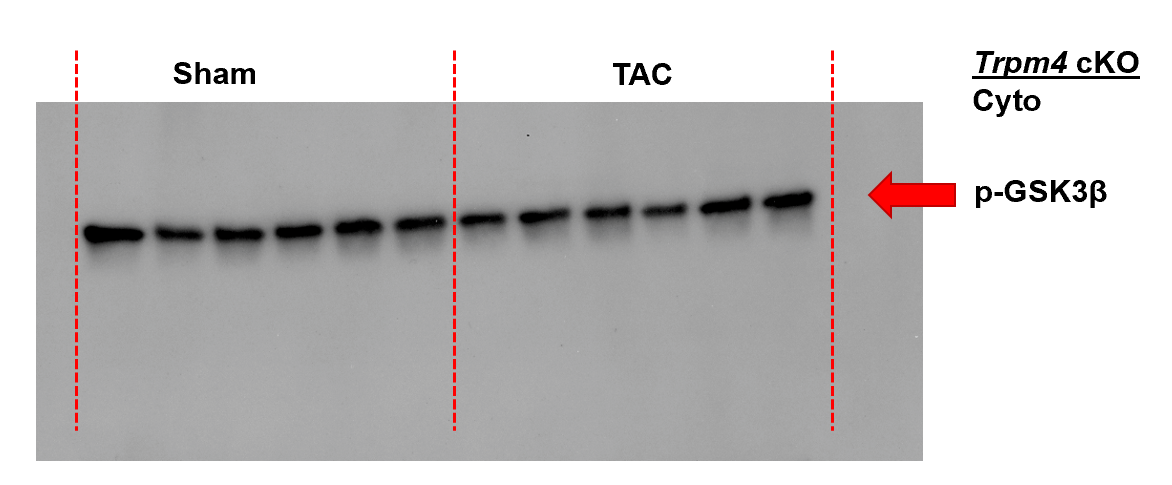

Supplement: Source data 1. [file elife-66582-data1.zip › Blots and Blot Figs/Blot Figs/KO Cyto/KO Cyto p-GSK3b Fig.tif]

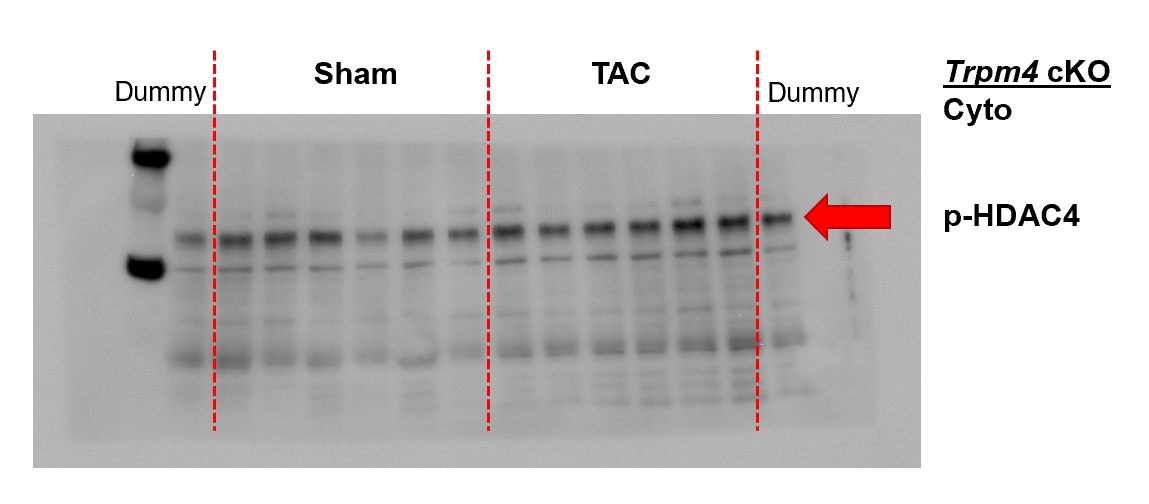

Supplement: Source data 1. [file elife-66582-data1.zip › Blots and Blot Figs/Blot Figs/KO Cyto/KO Cyto p-HDAC4 Fig.tif]

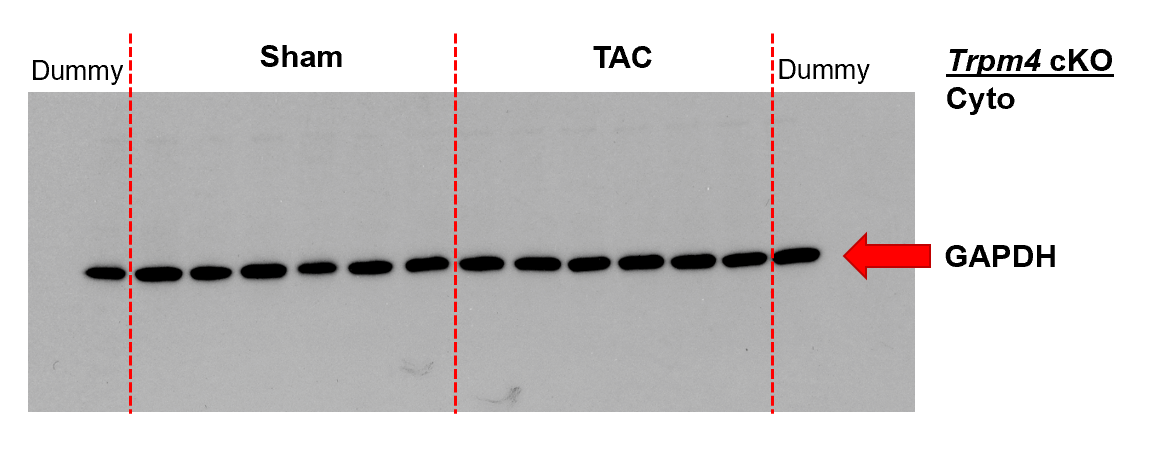

Supplement: Source data 1. [file elife-66582-data1.zip › Blots and Blot Figs/Blot Figs/KO Cyto/KO GAPDH1 Fig.tif]

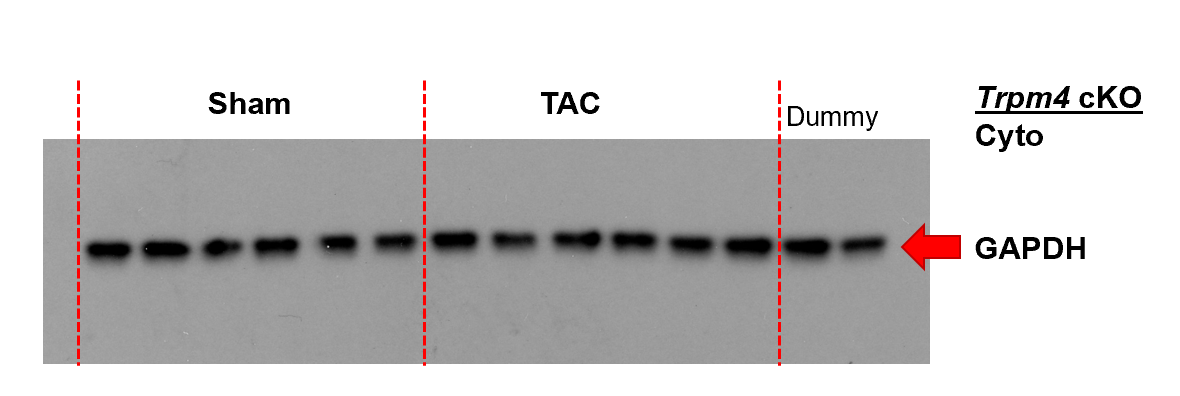

Supplement: Source data 1. [file elife-66582-data1.zip › Blots and Blot Figs/Blot Figs/KO Cyto/KO GAPDH2 Fig.tif]

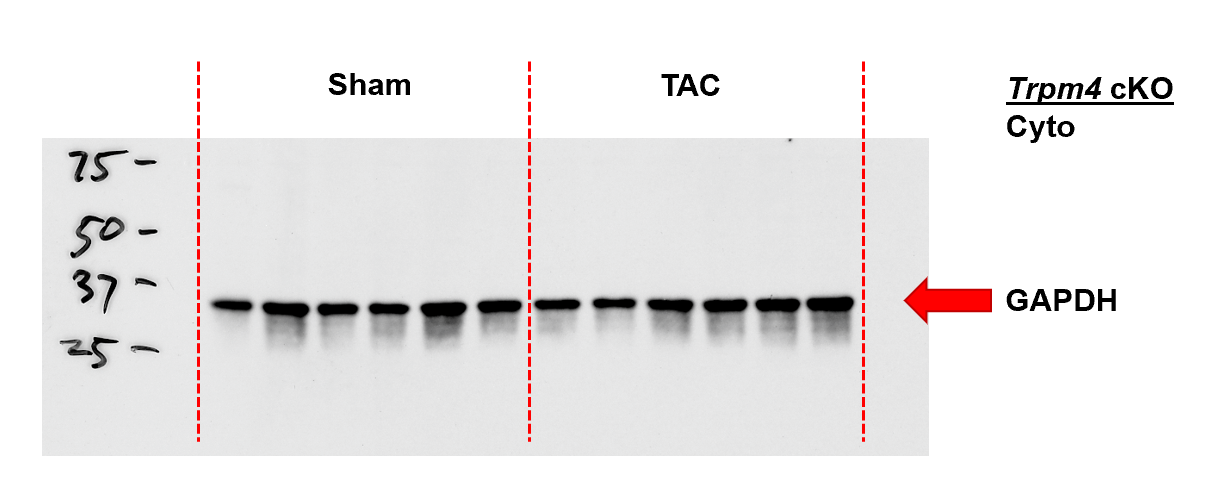

Supplement: Source data 1. [file elife-66582-data1.zip › Blots and Blot Figs/Blot Figs/KO Cyto/KO GAPDH3 Fig.tif]

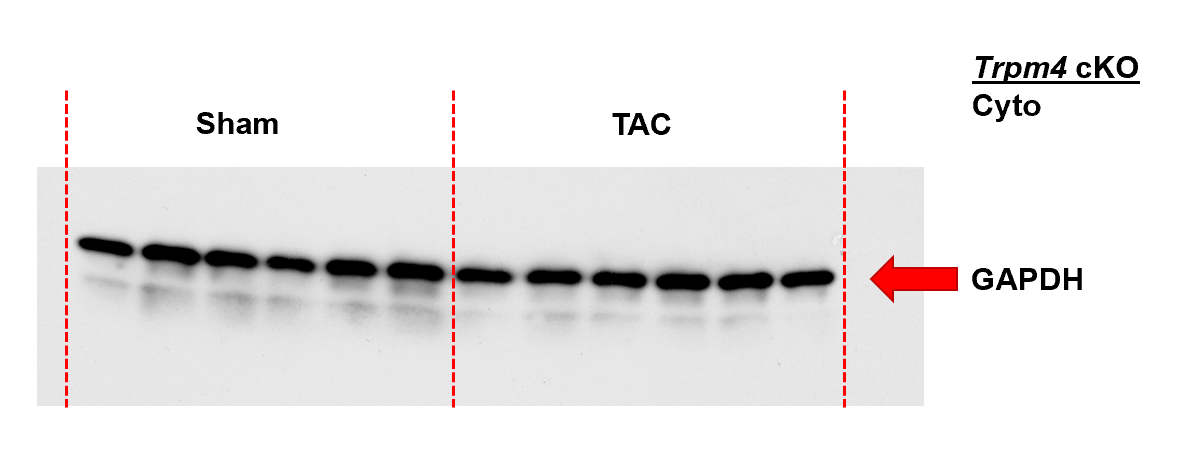

Supplement: Source data 1. [file elife-66582-data1.zip › Blots and Blot Figs/Blot Figs/KO Cyto/KO GAPDH4 Fig.tif]

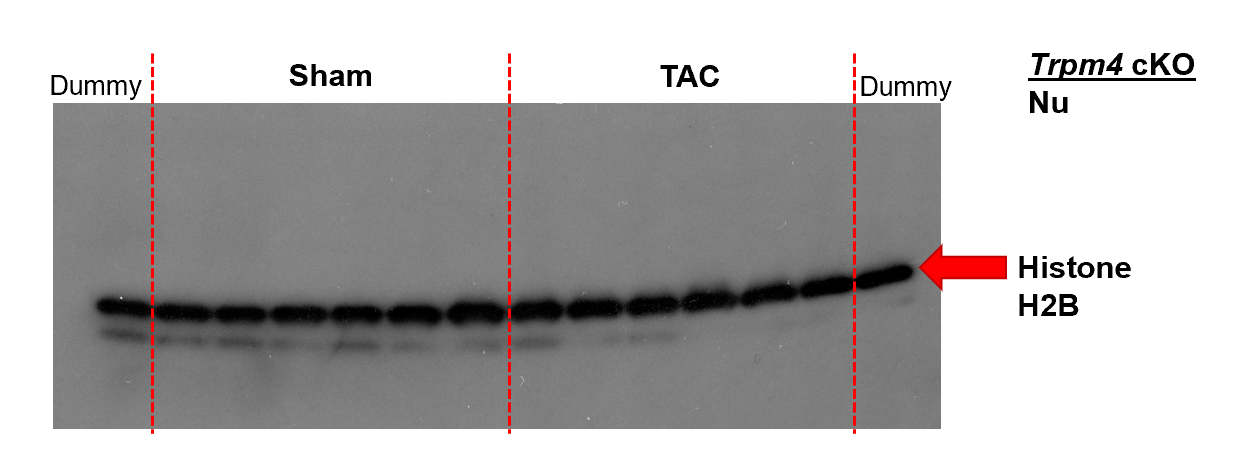

Supplement: Source data 1. [file elife-66582-data1.zip › Blots and Blot Figs/Blot Figs/KO Nu/KO Histone1 Fig.tif]

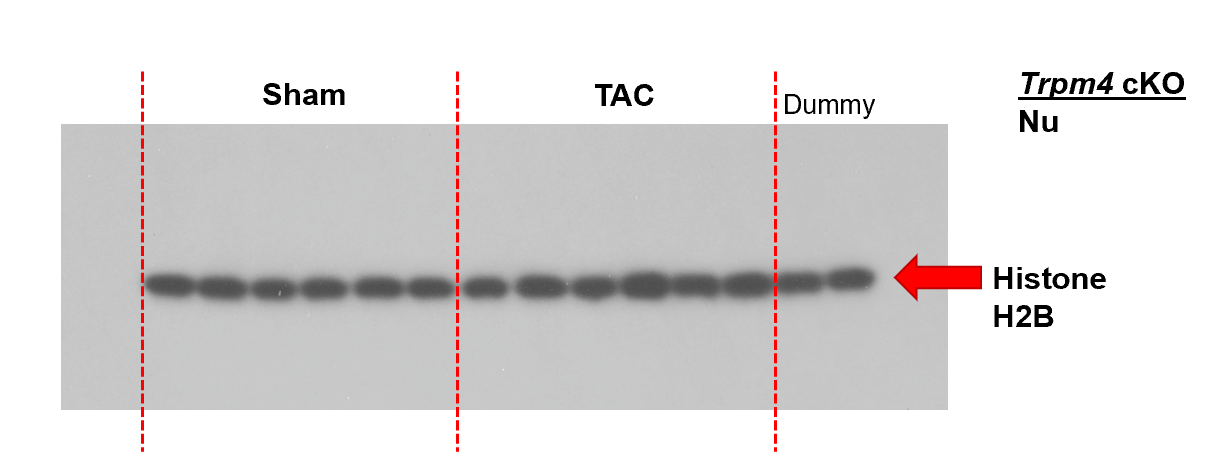

Supplement: Source data 1. [file elife-66582-data1.zip › Blots and Blot Figs/Blot Figs/KO Nu/KO Histone2 Fig.tif]

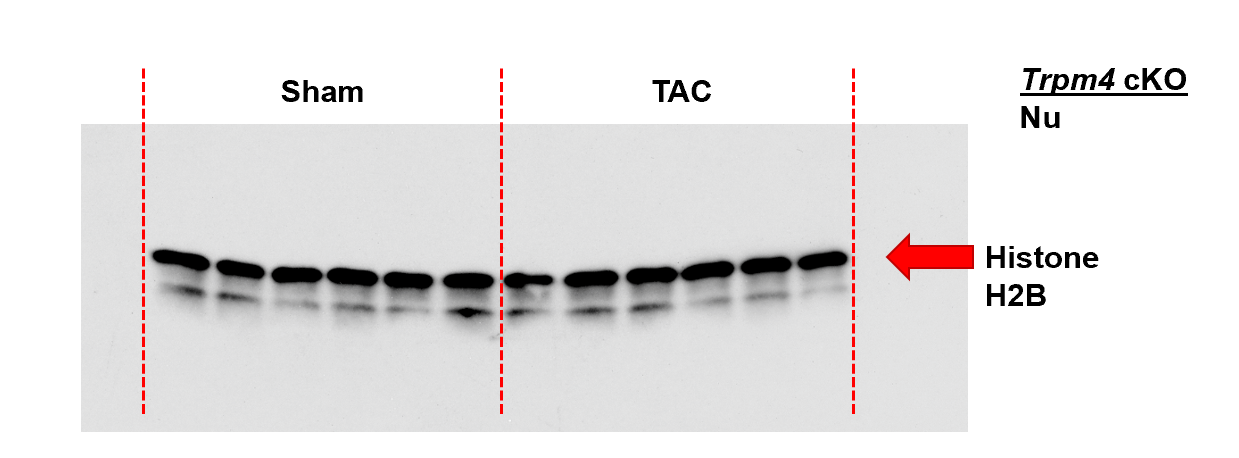

Supplement: Source data 1. [file elife-66582-data1.zip › Blots and Blot Figs/Blot Figs/KO Nu/KO Histone3 Fig.tif]

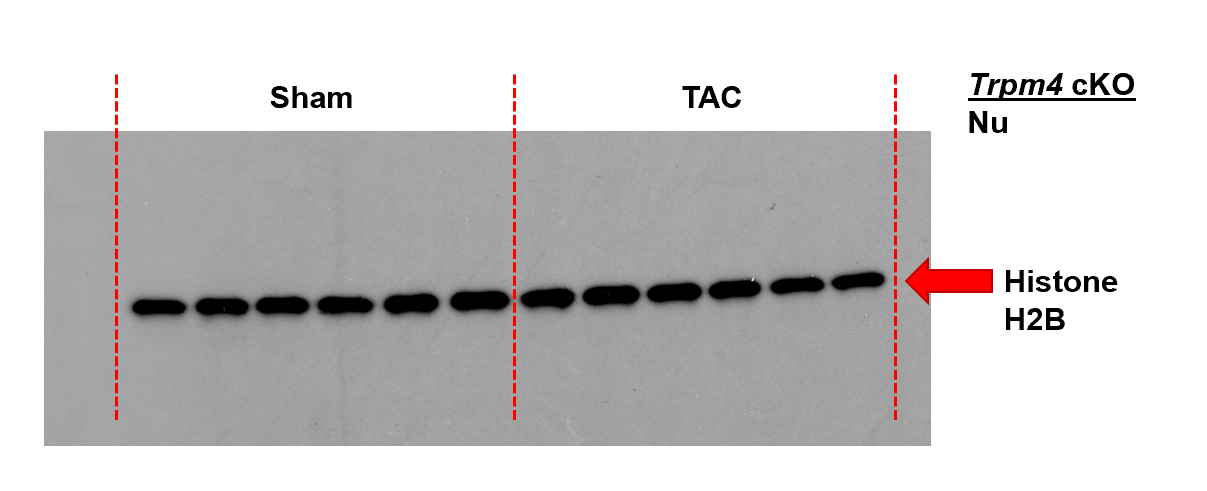

Supplement: Source data 1. [file elife-66582-data1.zip › Blots and Blot Figs/Blot Figs/KO Nu/KO Histone4 Fig.tif]

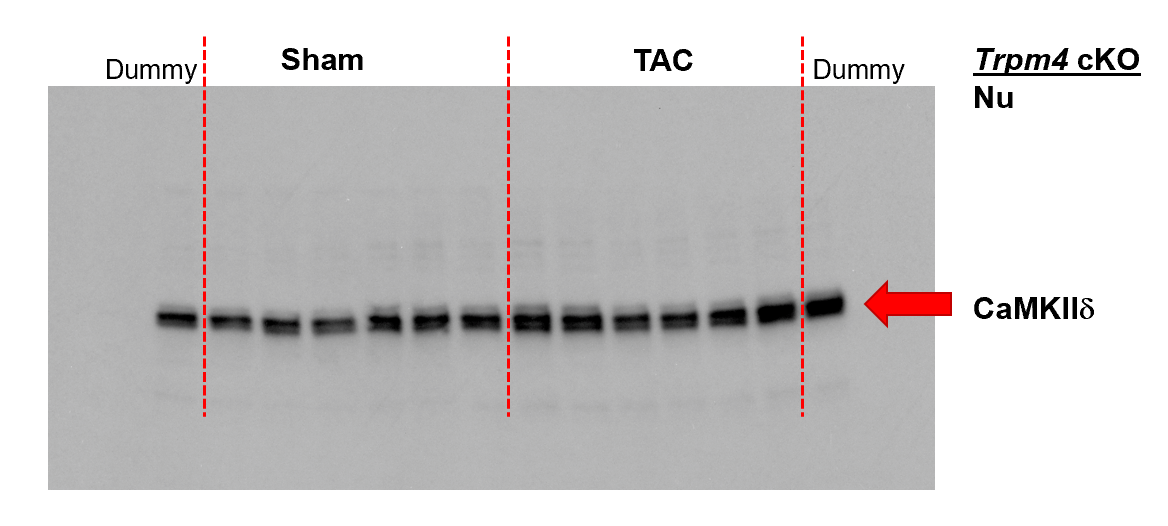

Supplement: Source data 1. [file elife-66582-data1.zip › Blots and Blot Figs/Blot Figs/KO Nu/KO Nu CaMK2 Fig.tif]

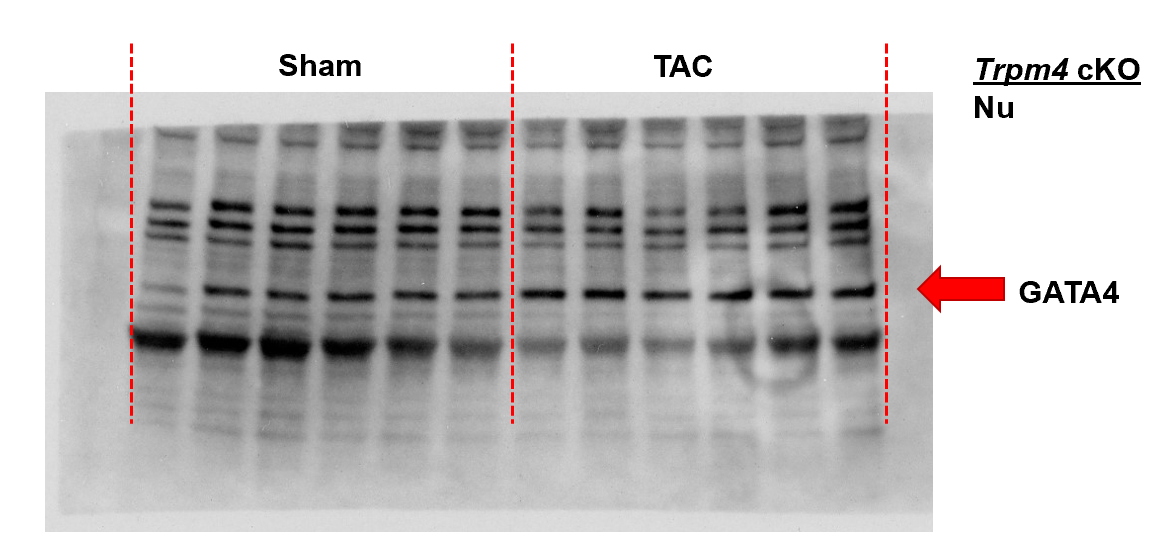

Supplement: Source data 1. [file elife-66582-data1.zip › Blots and Blot Figs/Blot Figs/KO Nu/KO Nu GATA4 Fig.tif]

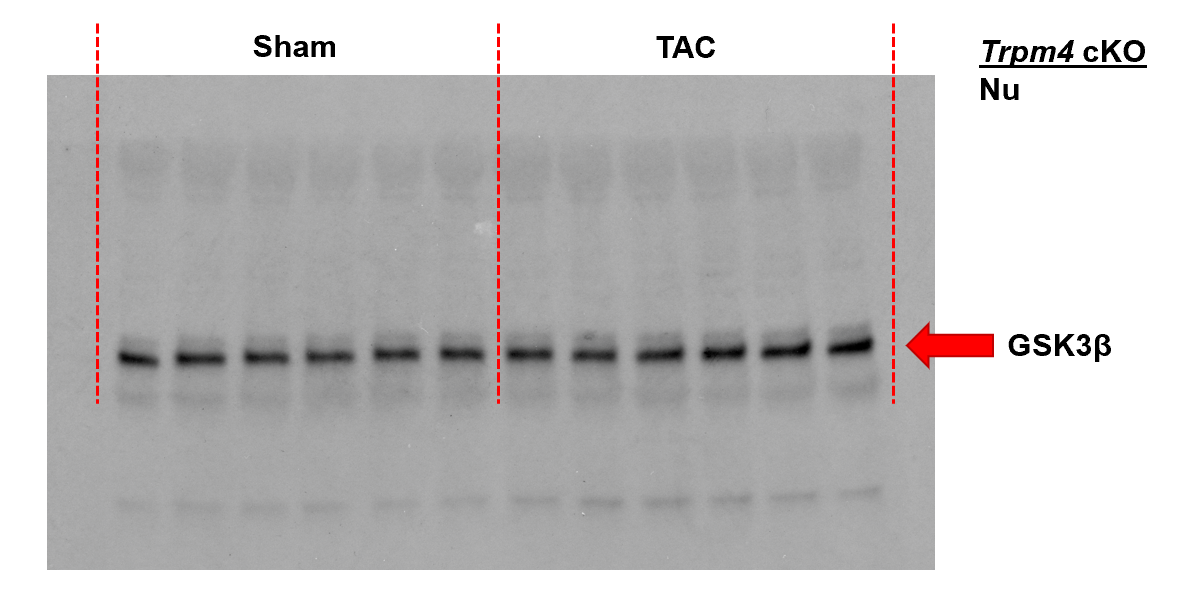

Supplement: Source data 1. [file elife-66582-data1.zip › Blots and Blot Figs/Blot Figs/KO Nu/KO Nu GSK3b Fig.tif]

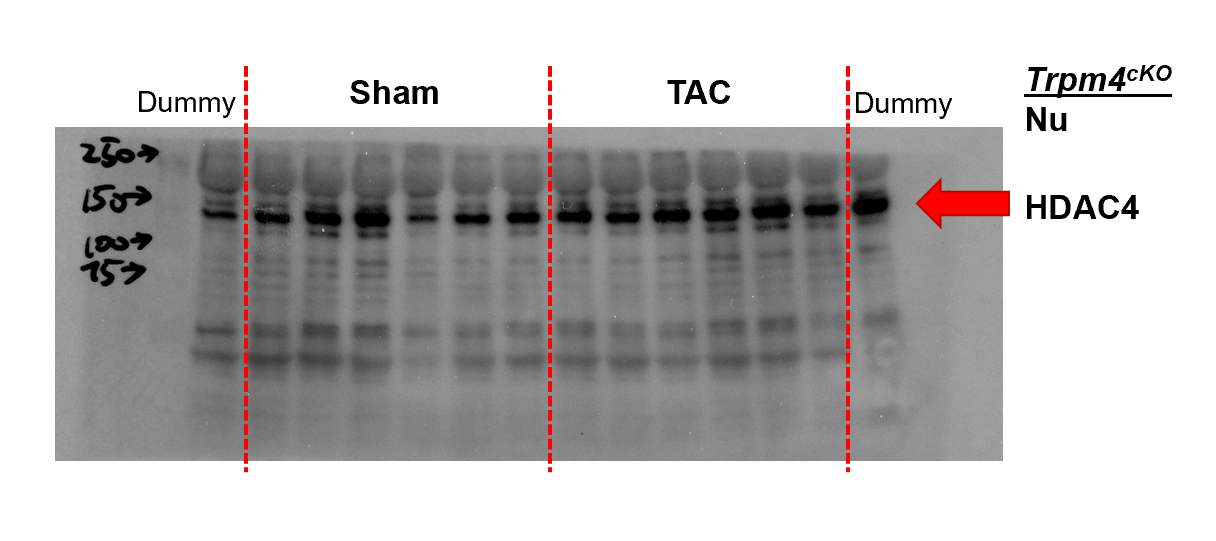

Supplement: Source data 1. [file elife-66582-data1.zip › Blots and Blot Figs/Blot Figs/KO Nu/KO Nu HDAC4 Fig.tif]

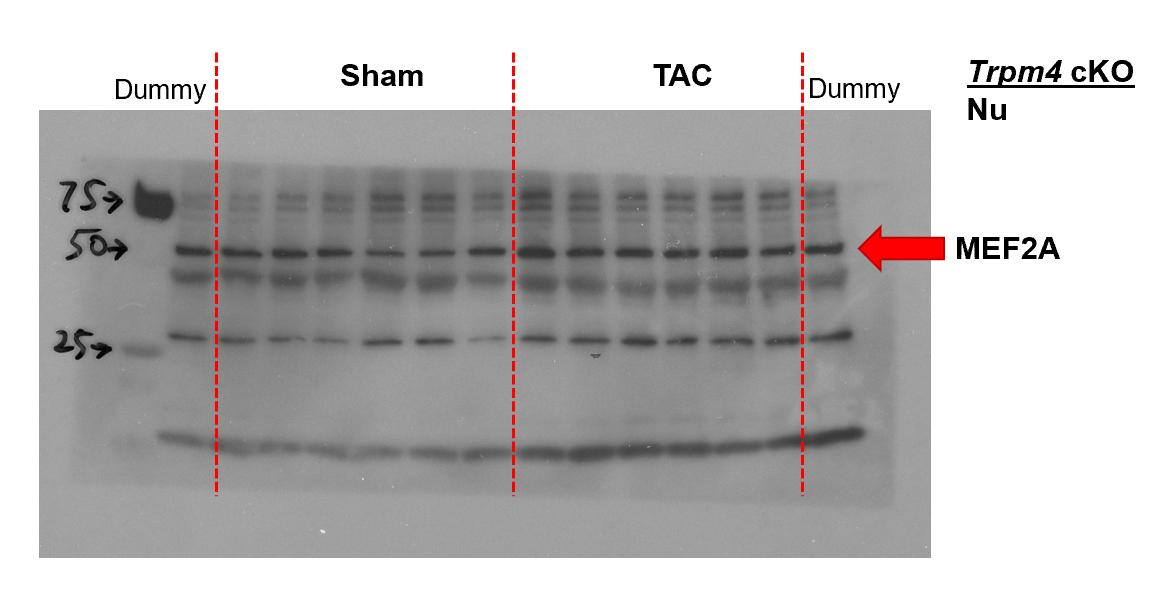

Supplement: Source data 1. [file elife-66582-data1.zip › Blots and Blot Figs/Blot Figs/KO Nu/KO Nu MEF2A Fig.tif]

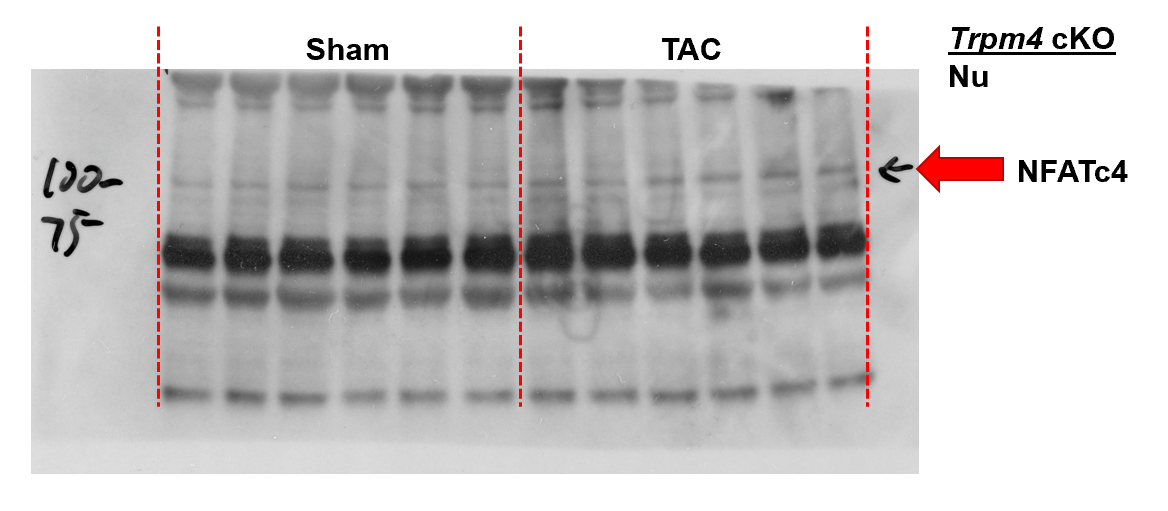

Supplement: Source data 1. [file elife-66582-data1.zip › Blots and Blot Figs/Blot Figs/KO Nu/KO Nu NFAT Fig.tif]

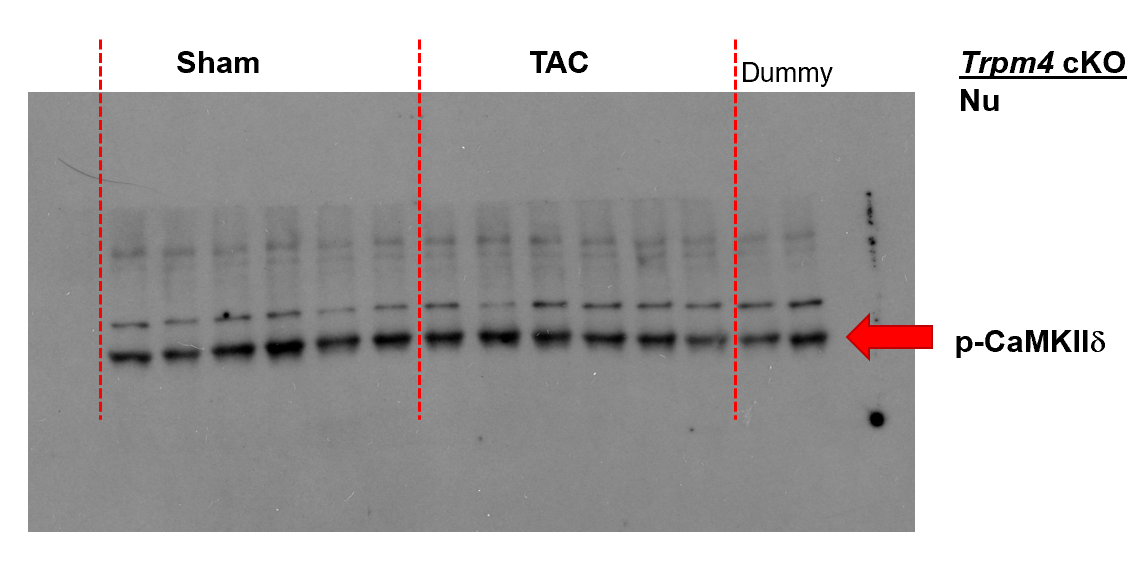

Supplement: Source data 1. [file elife-66582-data1.zip › Blots and Blot Figs/Blot Figs/KO Nu/KO Nu p-CaMK2 Fig.tif]

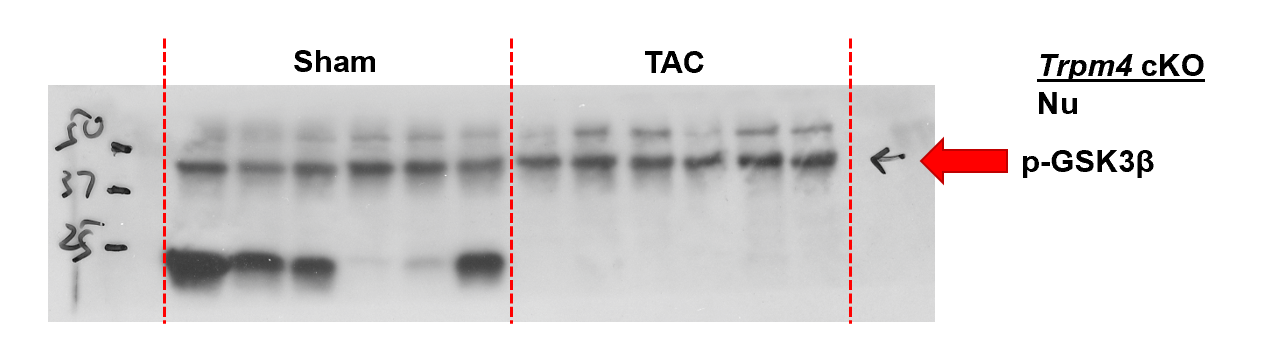

Supplement: Source data 1. [file elife-66582-data1.zip › Blots and Blot Figs/Blot Figs/KO Nu/KO Nu p-GSK3b Fig.tif]

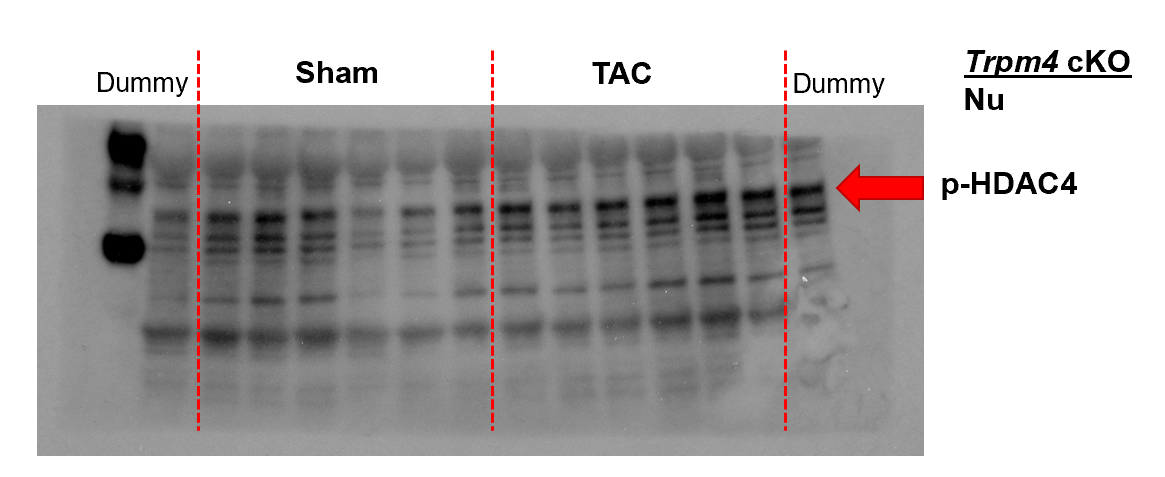

Supplement: Source data 1. [file elife-66582-data1.zip › Blots and Blot Figs/Blot Figs/KO Nu/KO Nu p-HDAC4 Fig.tif]

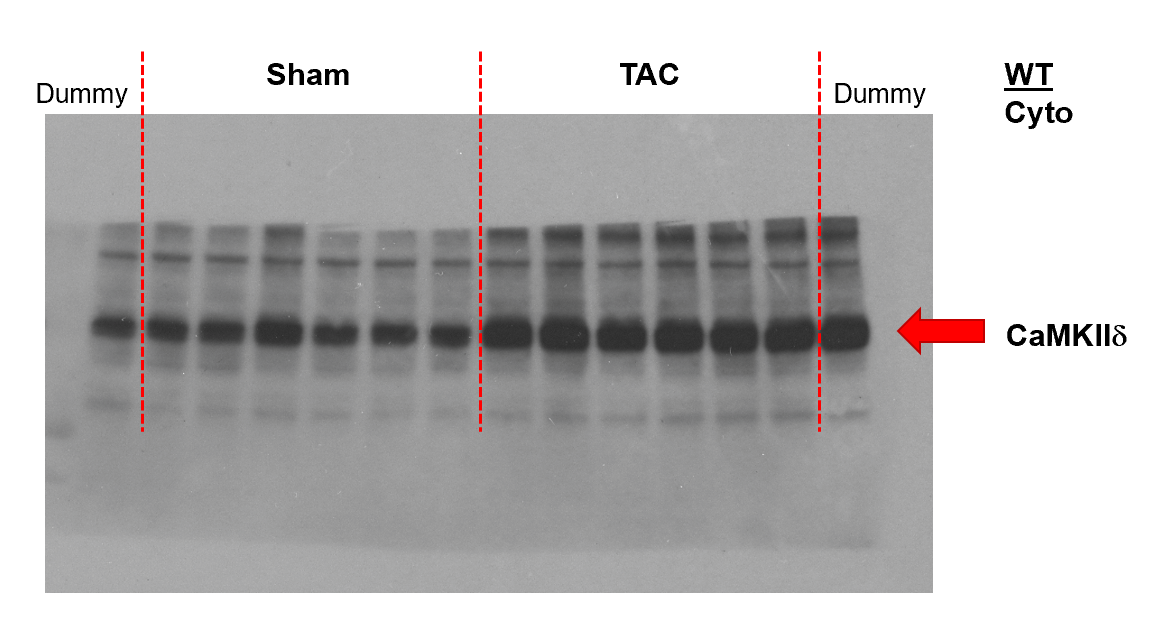

Supplement: Source data 1. [file elife-66582-data1.zip › Blots and Blot Figs/Blot Figs/WT Cyto/WT Cyto CaMK2 Fig.tif]

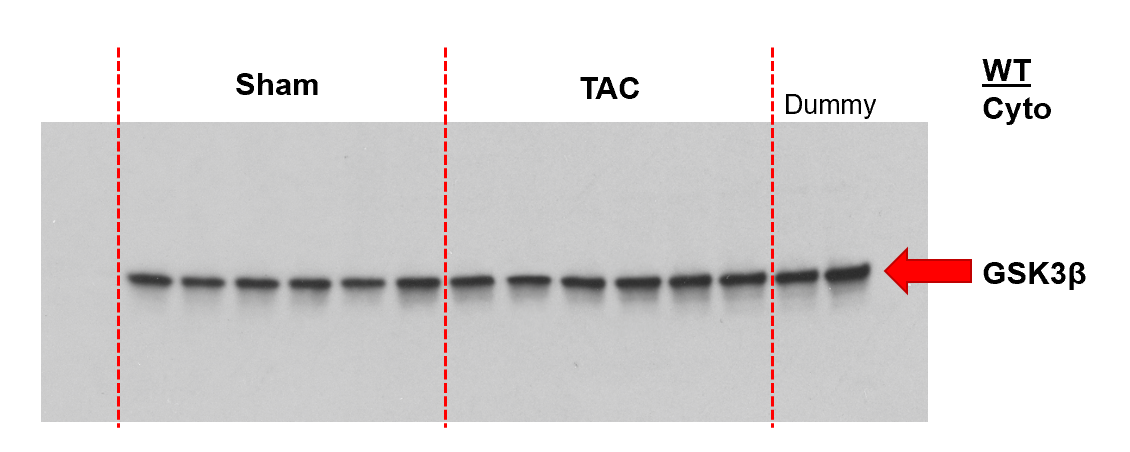

Supplement: Source data 1. [file elife-66582-data1.zip › Blots and Blot Figs/Blot Figs/WT Cyto/WT Cyto GSK3b Fig.tif]

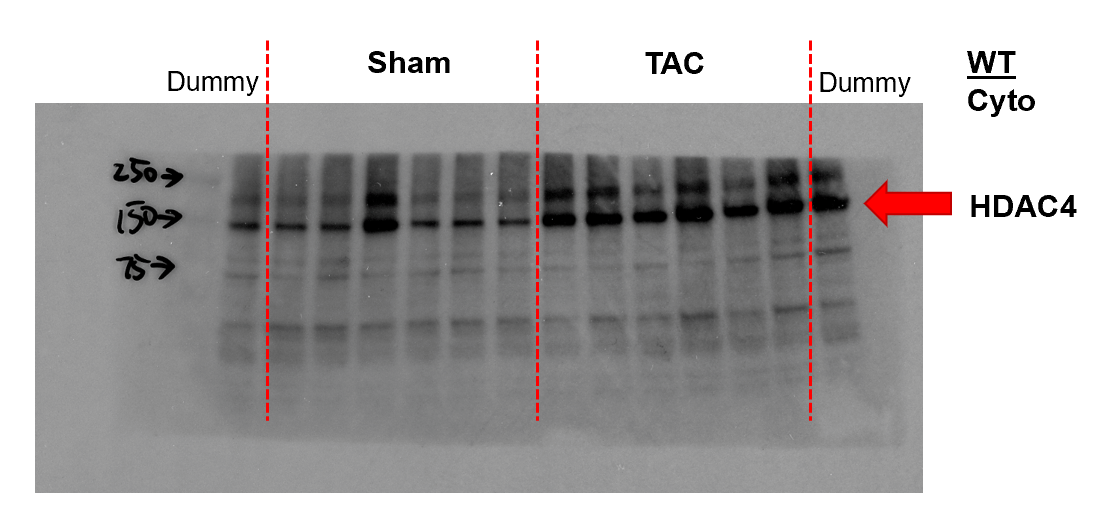

Supplement: Source data 1. [file elife-66582-data1.zip › Blots and Blot Figs/Blot Figs/WT Cyto/WT Cyto HDAC4 Fig.tif]

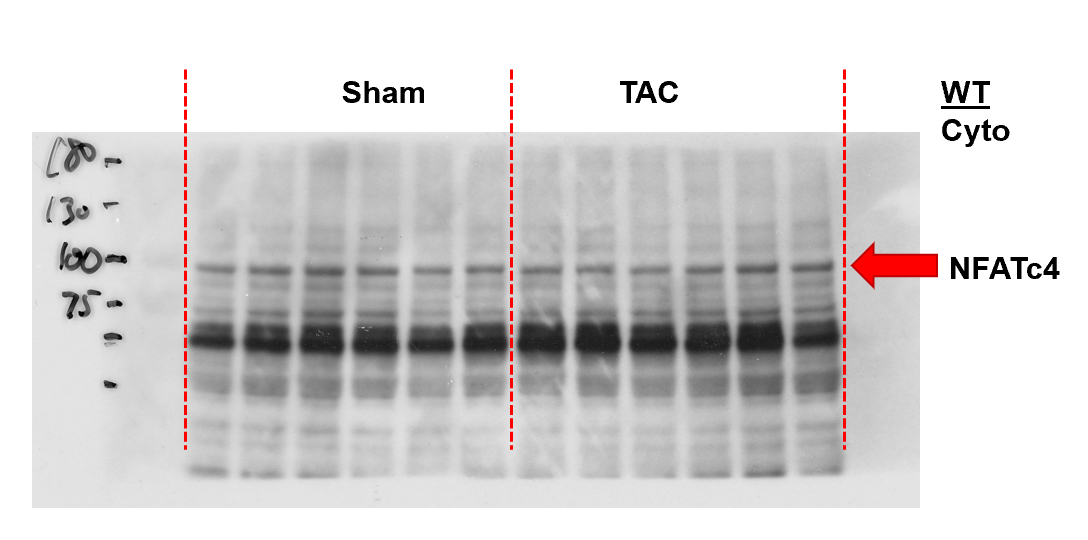

Supplement: Source data 1. [file elife-66582-data1.zip › Blots and Blot Figs/Blot Figs/WT Cyto/WT Cyto NFAT Fig.tif]

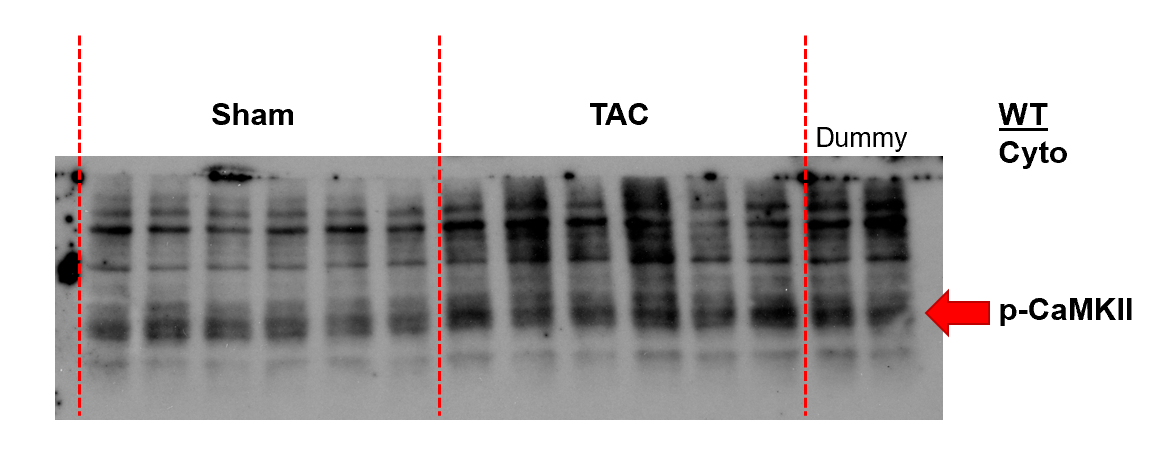

Supplement: Source data 1. [file elife-66582-data1.zip › Blots and Blot Figs/Blot Figs/WT Cyto/WT Cyto p-CaMK2 Fig.tif]

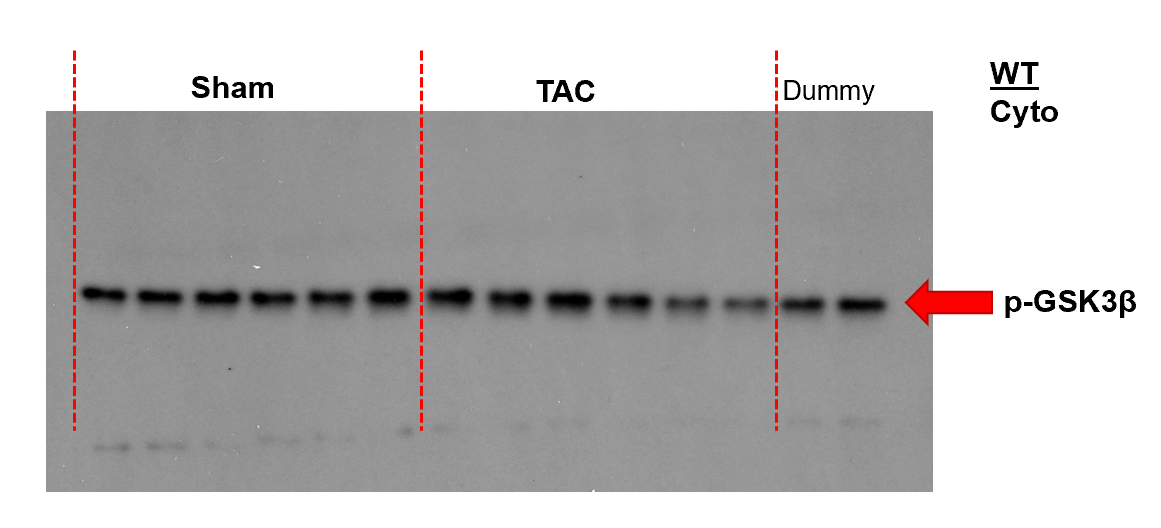

Supplement: Source data 1. [file elife-66582-data1.zip › Blots and Blot Figs/Blot Figs/WT Cyto/WT Cyto p-GSK3b Fig.tif]

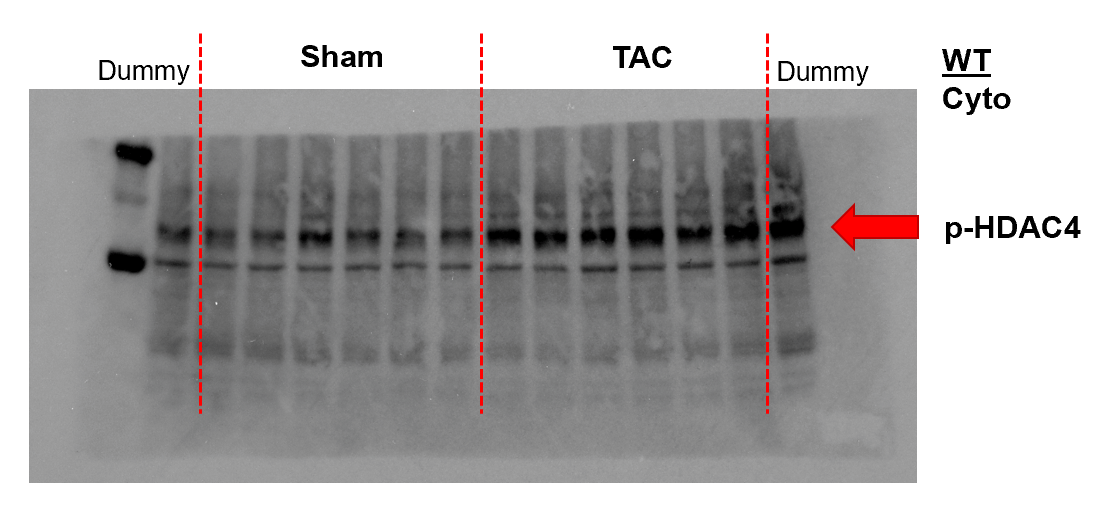

Supplement: Source data 1. [file elife-66582-data1.zip › Blots and Blot Figs/Blot Figs/WT Cyto/WT Cyto p-HDAC4 Fig.tif]

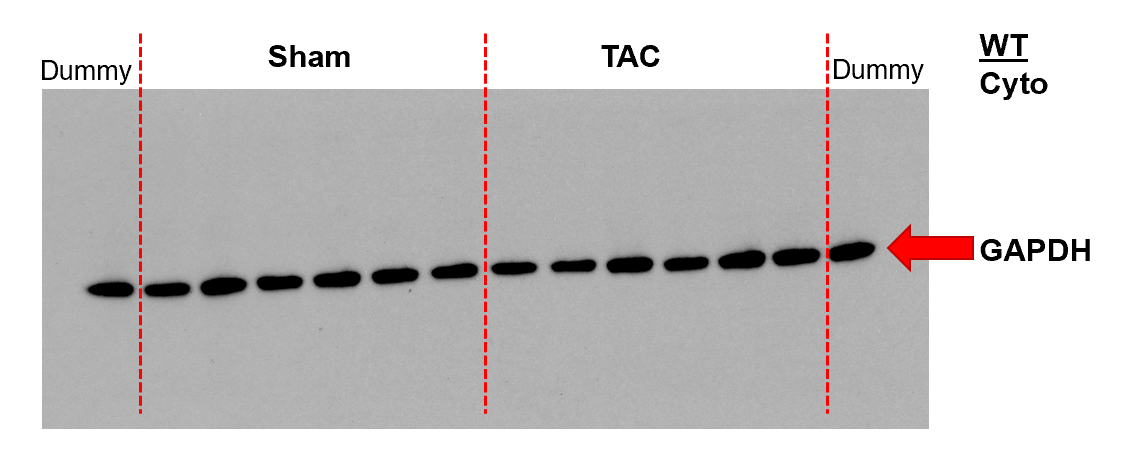

Supplement: Source data 1. [file elife-66582-data1.zip › Blots and Blot Figs/Blot Figs/WT Cyto/WT GAPDH1 Fig.tif]

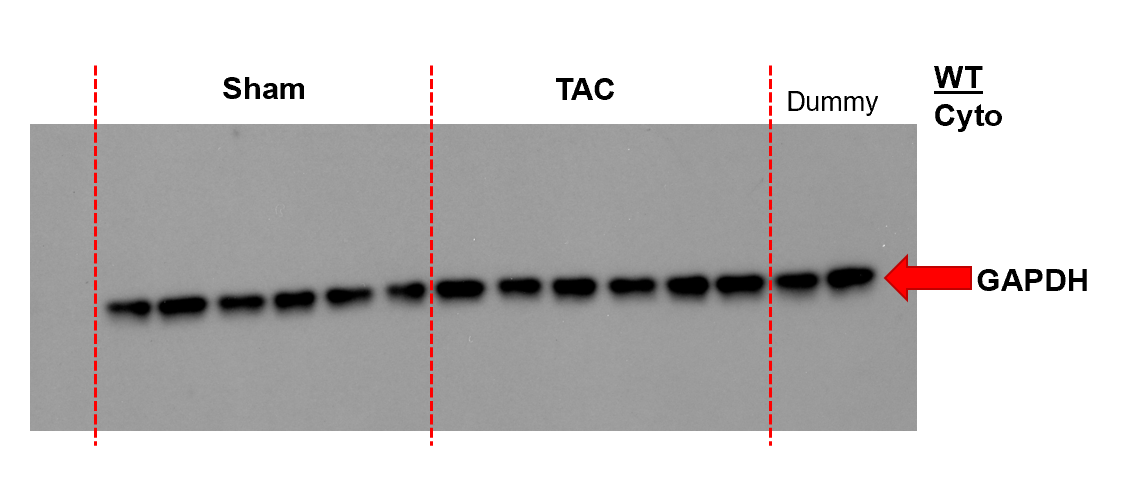

Supplement: Source data 1. [file elife-66582-data1.zip › Blots and Blot Figs/Blot Figs/WT Cyto/WT GAPDH2 Fig.tif]

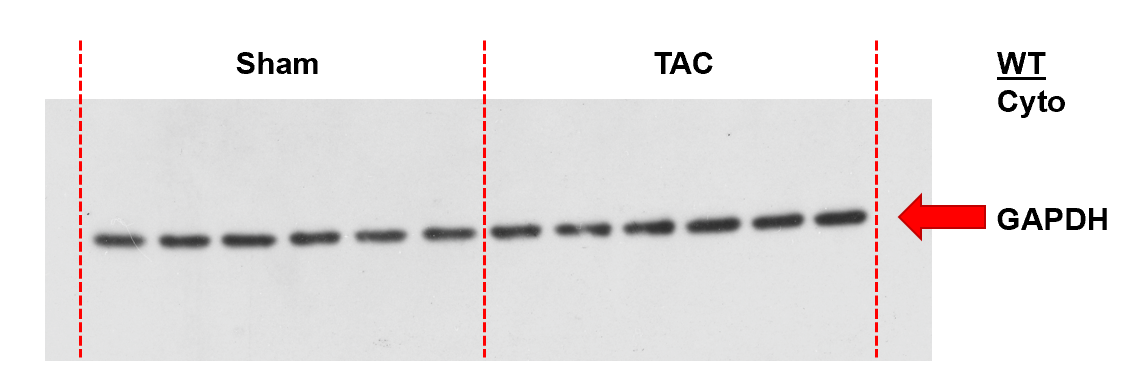

Supplement: Source data 1. [file elife-66582-data1.zip › Blots and Blot Figs/Blot Figs/WT Cyto/WT GAPDH3 Fig.tif]

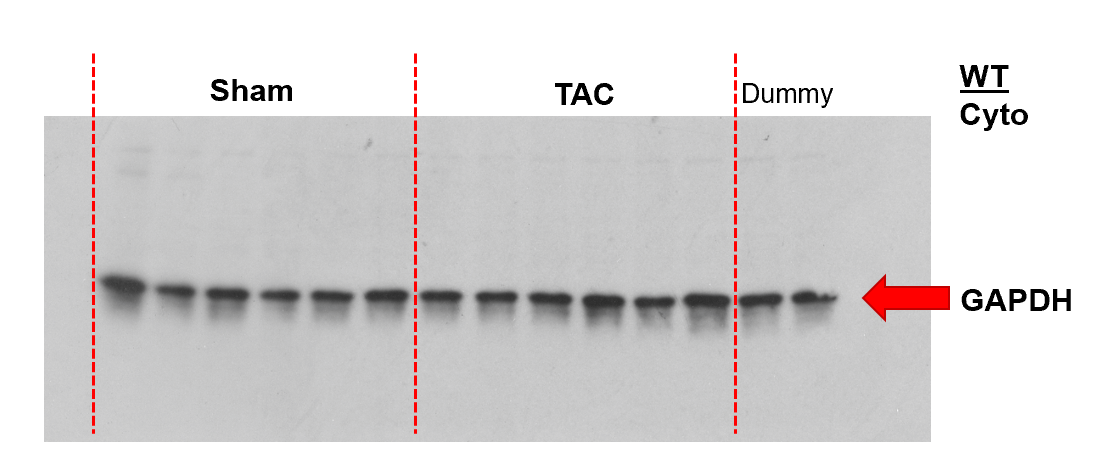

Supplement: Source data 1. [file elife-66582-data1.zip › Blots and Blot Figs/Blot Figs/WT Cyto/WT GAPDH4 Fig.tif]

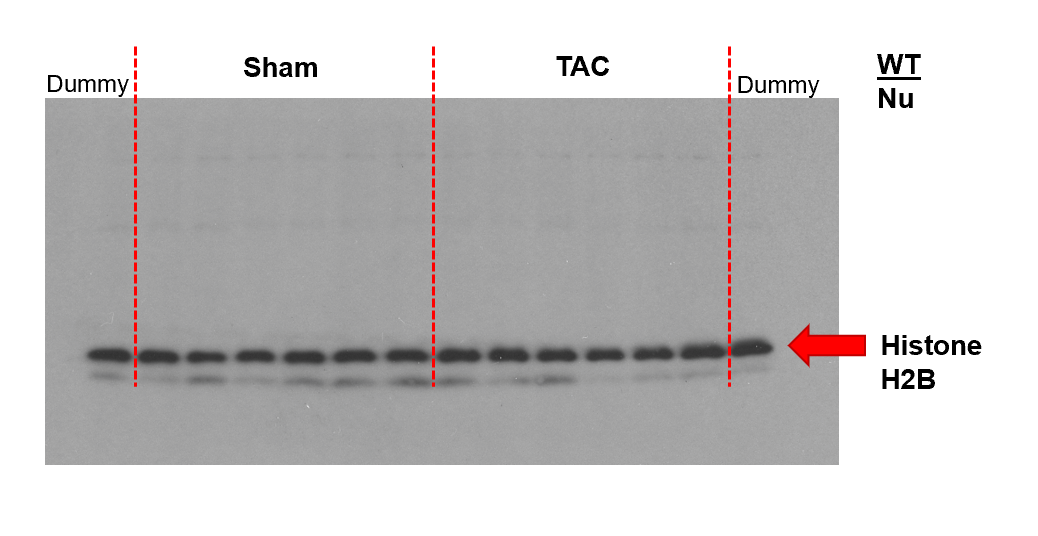

Supplement: Source data 1. [file elife-66582-data1.zip › Blots and Blot Figs/Blot Figs/WT Nu/WT Histone1 Fig.tif]

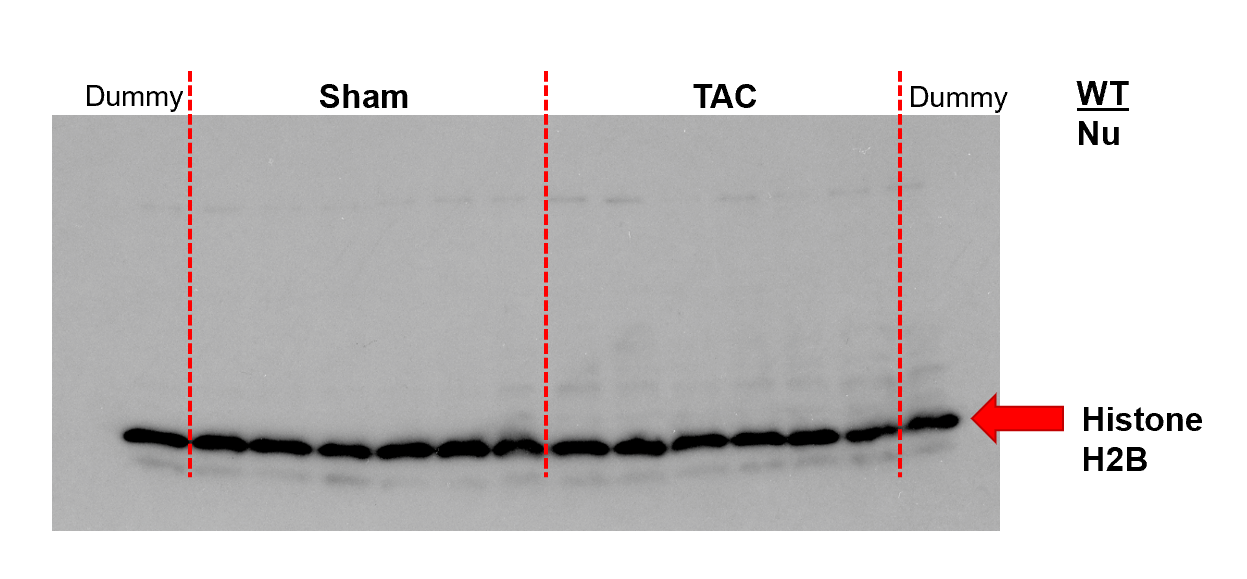

Supplement: Source data 1. [file elife-66582-data1.zip › Blots and Blot Figs/Blot Figs/WT Nu/WT Histone2 Fig.tif]

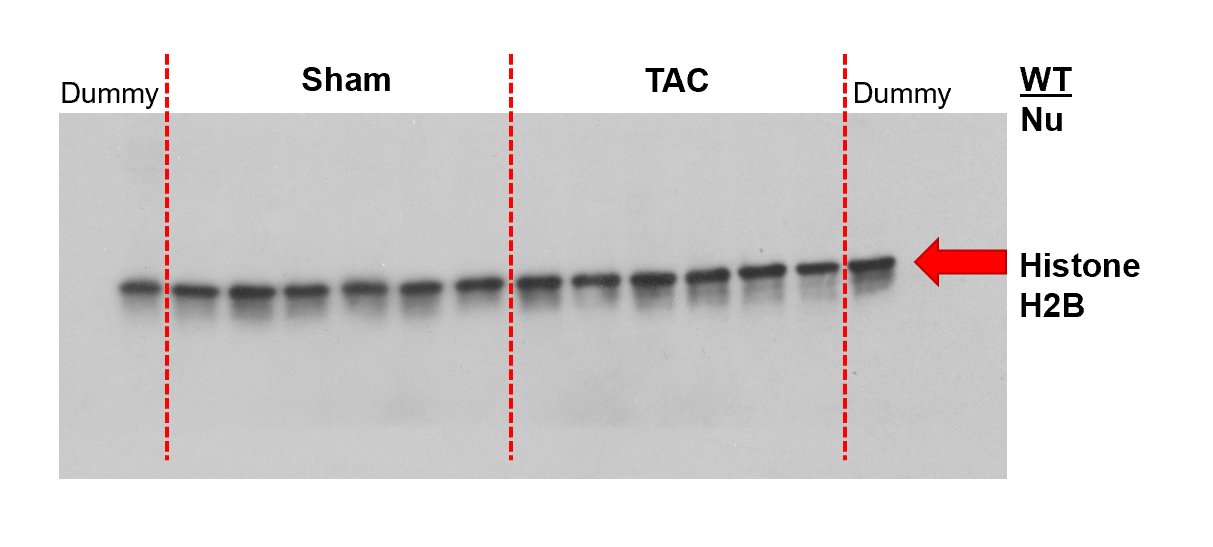

Supplement: Source data 1. [file elife-66582-data1.zip › Blots and Blot Figs/Blot Figs/WT Nu/WT Histone3 Fig.tif]

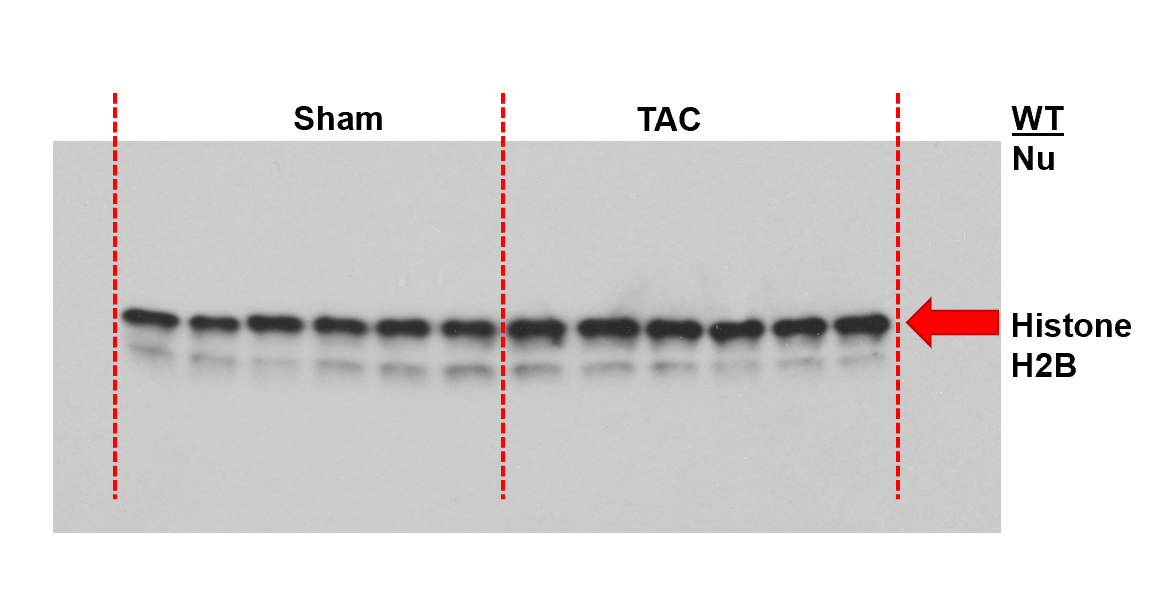

Supplement: Source data 1. [file elife-66582-data1.zip › Blots and Blot Figs/Blot Figs/WT Nu/WT Histone4 Fig.tif]

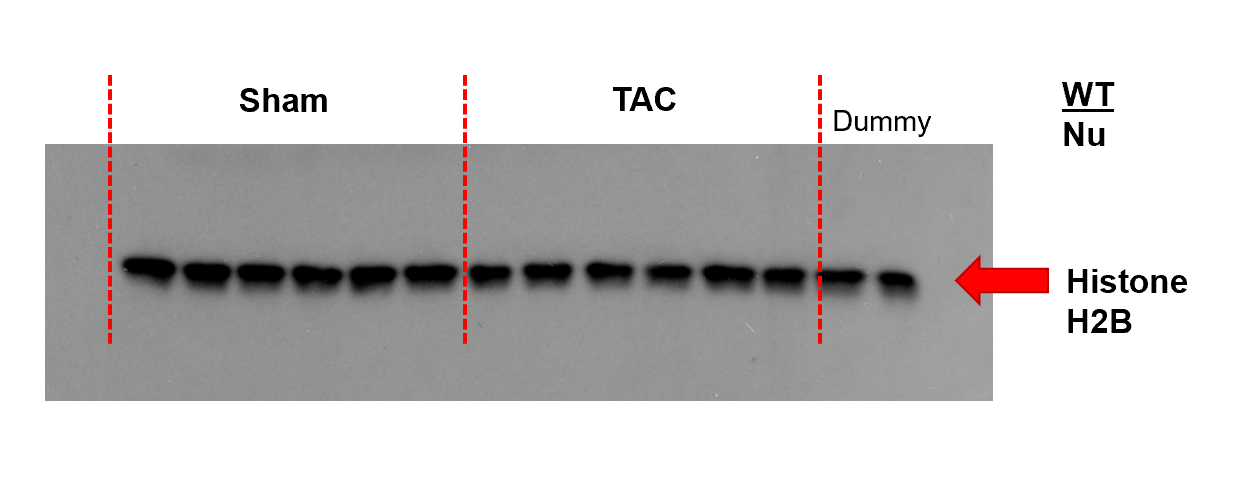

Supplement: Source data 1. [file elife-66582-data1.zip › Blots and Blot Figs/Blot Figs/WT Nu/WT Histone5 Fig.tif]

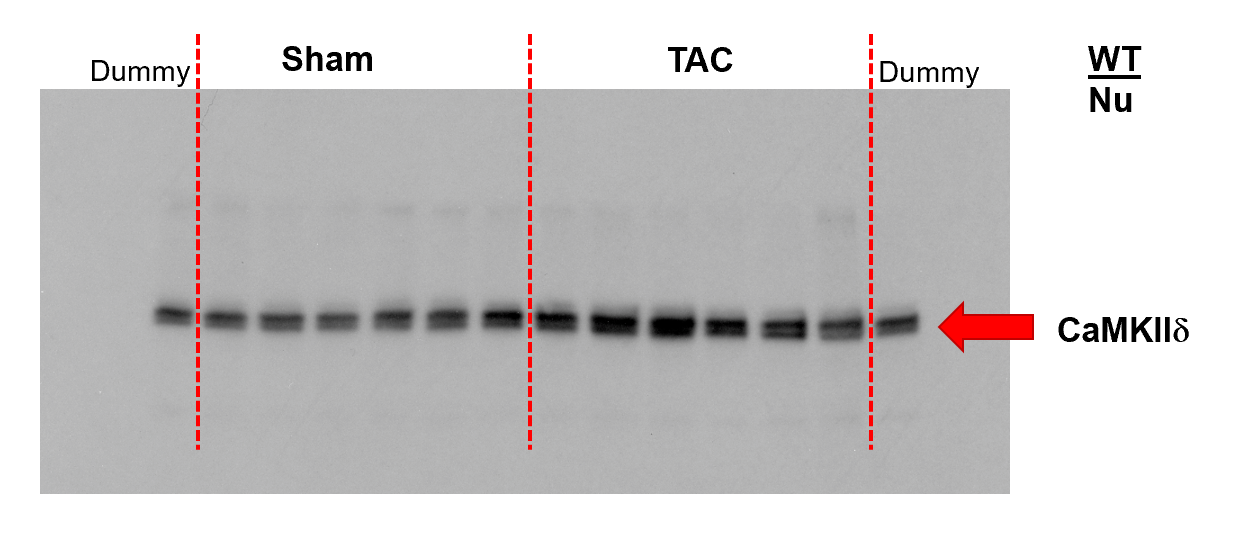

Supplement: Source data 1. [file elife-66582-data1.zip › Blots and Blot Figs/Blot Figs/WT Nu/WT Nu CaMK2 Fig.tif]

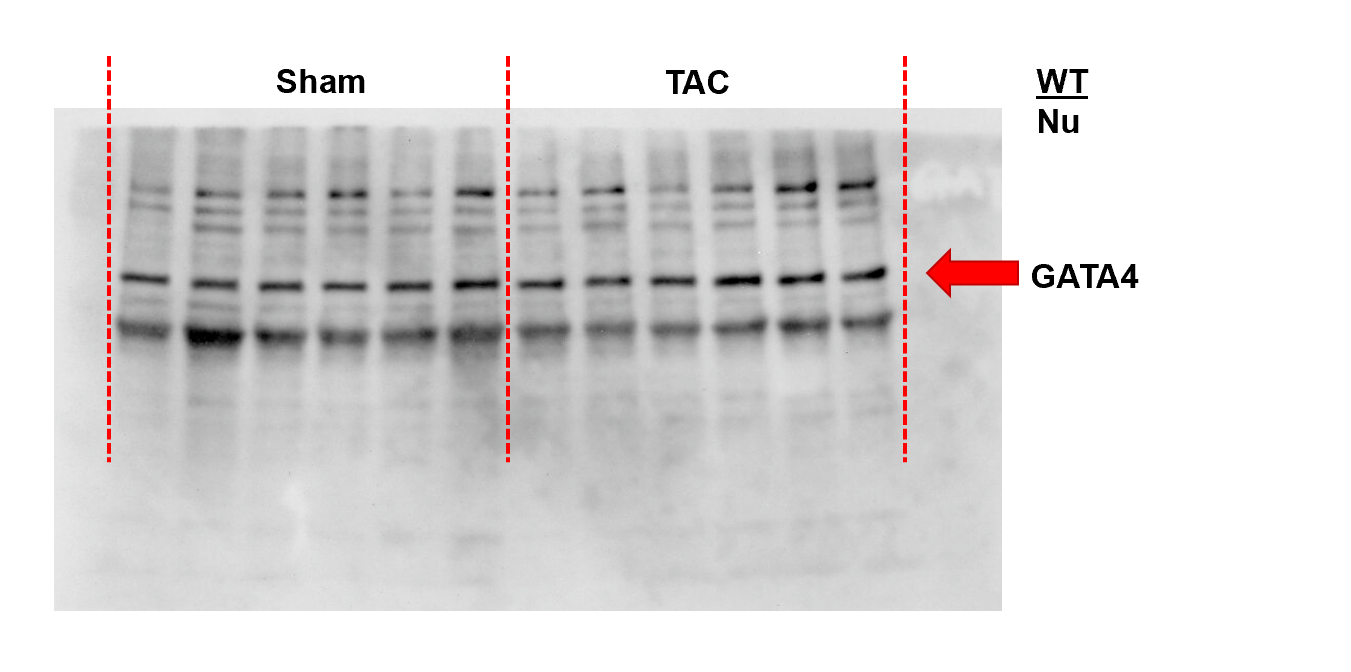

Supplement: Source data 1. [file elife-66582-data1.zip › Blots and Blot Figs/Blot Figs/WT Nu/WT Nu GATA4 Fig.tif]

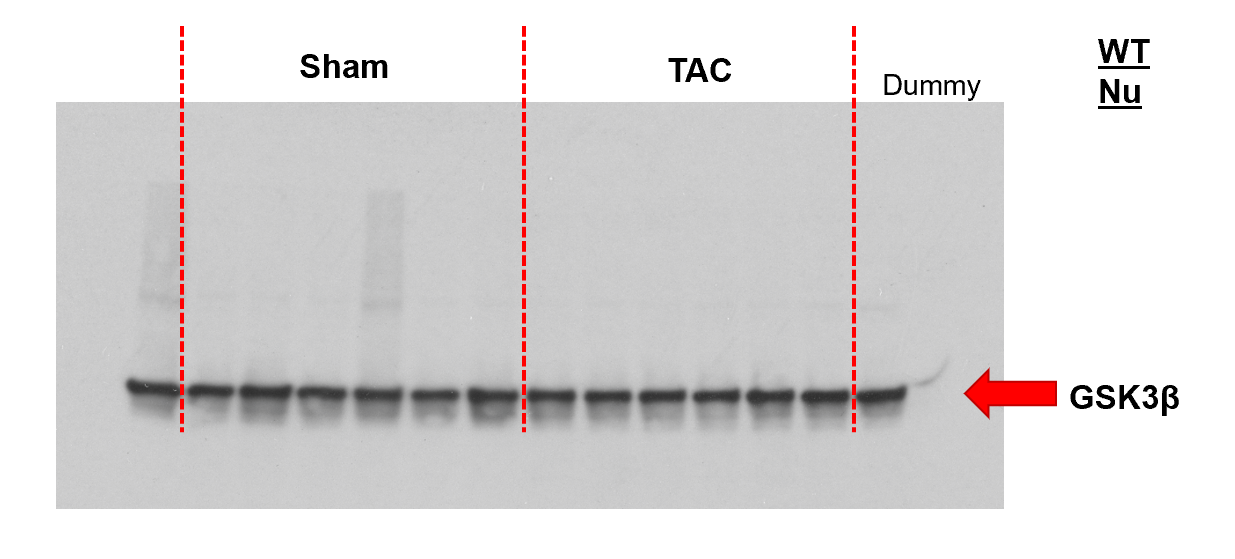

Supplement: Source data 1. [file elife-66582-data1.zip › Blots and Blot Figs/Blot Figs/WT Nu/WT Nu GSK3b Fig.tif]

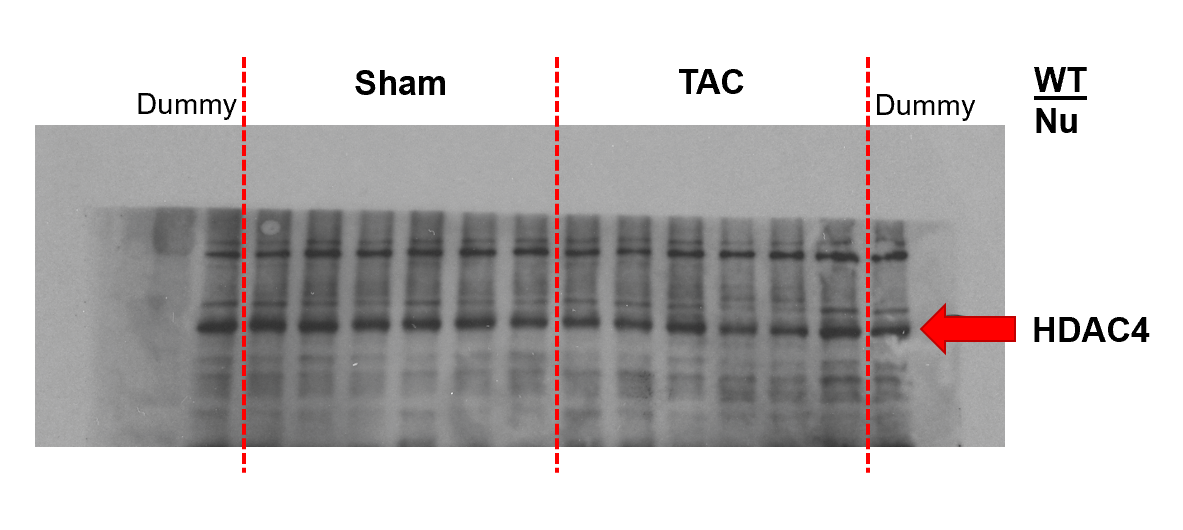

Supplement: Source data 1. [file elife-66582-data1.zip › Blots and Blot Figs/Blot Figs/WT Nu/WT Nu HDAC4 Fig.tif]

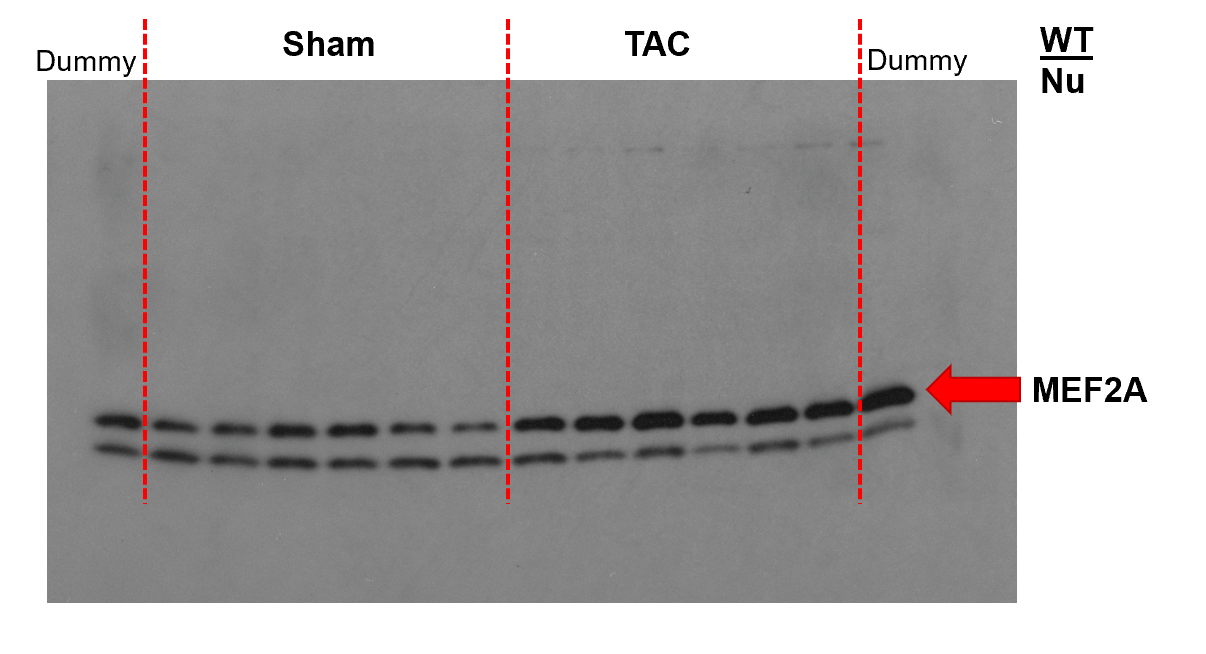

Supplement: Source data 1. [file elife-66582-data1.zip › Blots and Blot Figs/Blot Figs/WT Nu/WT Nu MEF2A Fig.tif]

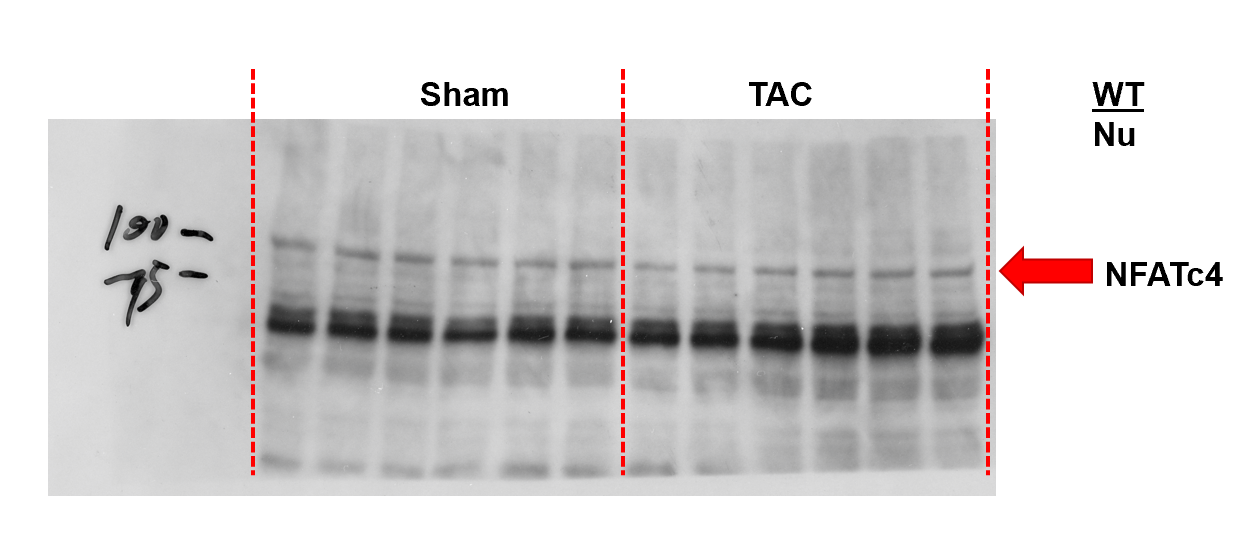

Supplement: Source data 1. [file elife-66582-data1.zip › Blots and Blot Figs/Blot Figs/WT Nu/WT Nu NFAT Fig.tif]

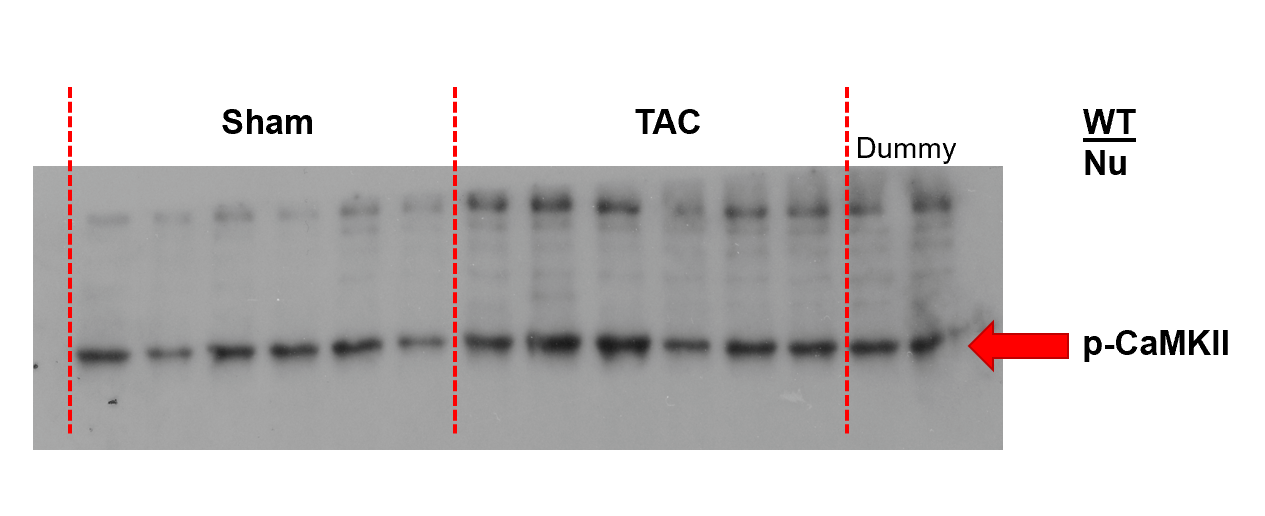

Supplement: Source data 1. [file elife-66582-data1.zip › Blots and Blot Figs/Blot Figs/WT Nu/WT Nu p-CaMK2 Fig.tif]

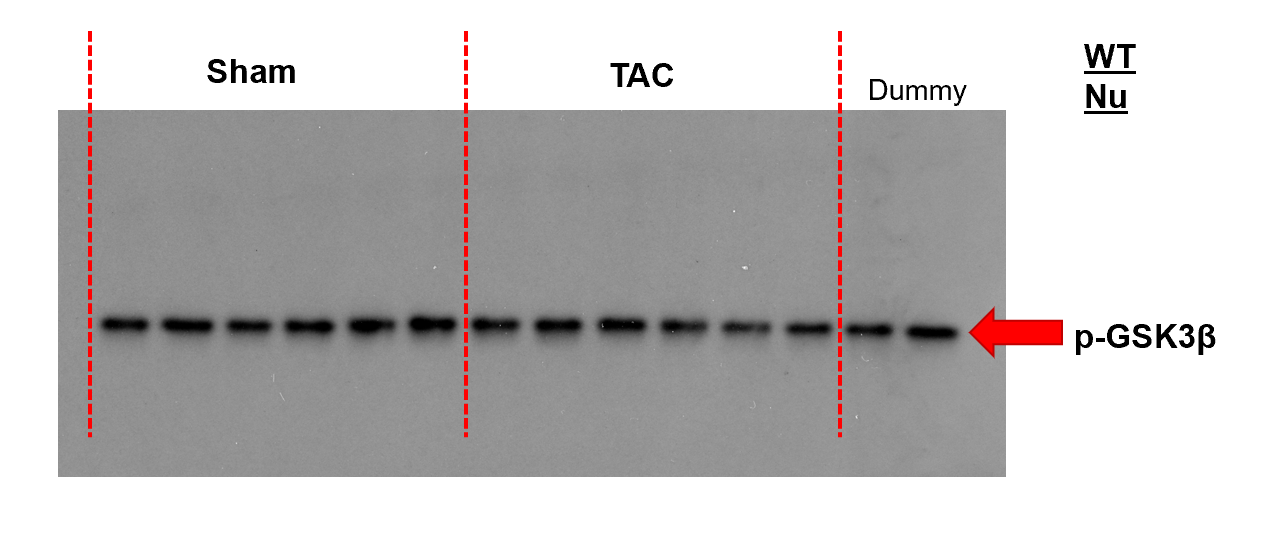

Supplement: Source data 1. [file elife-66582-data1.zip › Blots and Blot Figs/Blot Figs/WT Nu/WT Nu p-GSK3b Fig.tif]

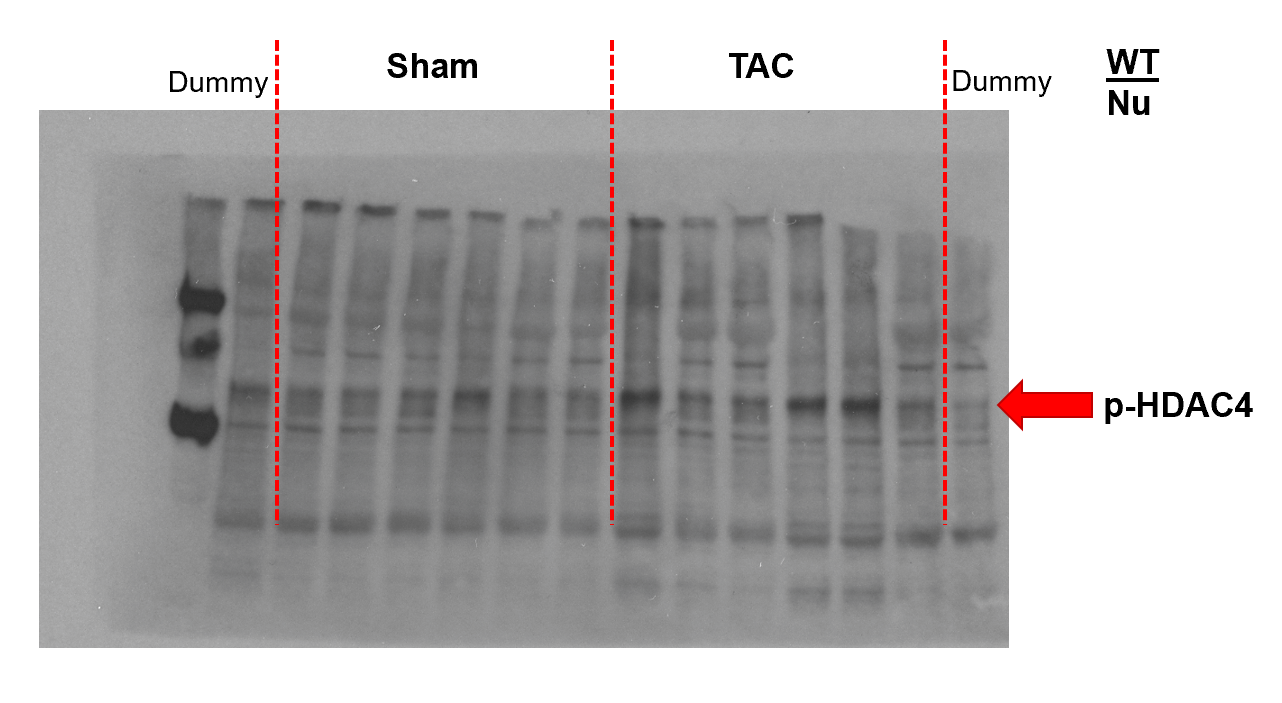

Supplement: Source data 1. [file elife-66582-data1.zip › Blots and Blot Figs/Blot Figs/WT Nu/WT Nu p-HDAC4 Fig.tif]

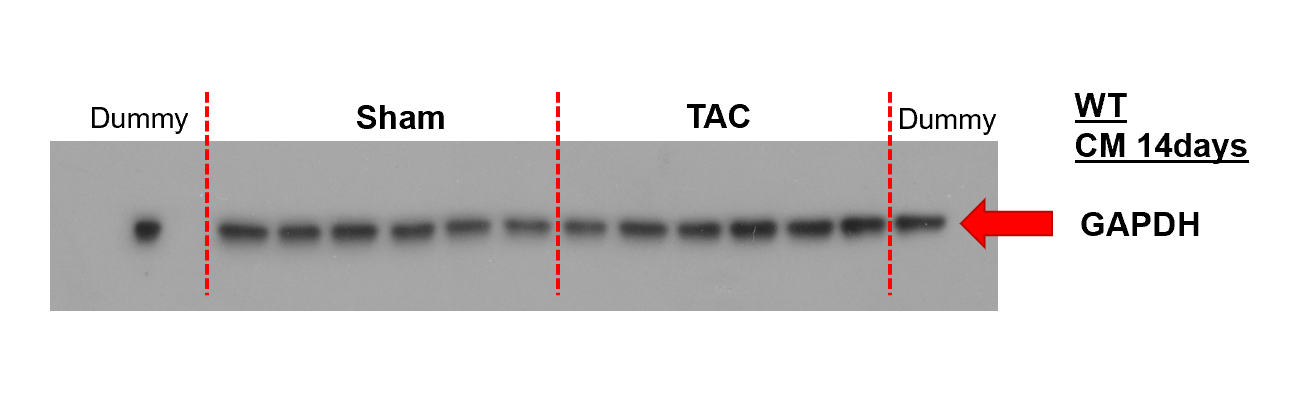

Supplement: Source data 1. [file elife-66582-data1.zip › Blots and Blot Figs/Blot Figs/WT TRPM4/WT CM 14days GAPDH Fig.tif]

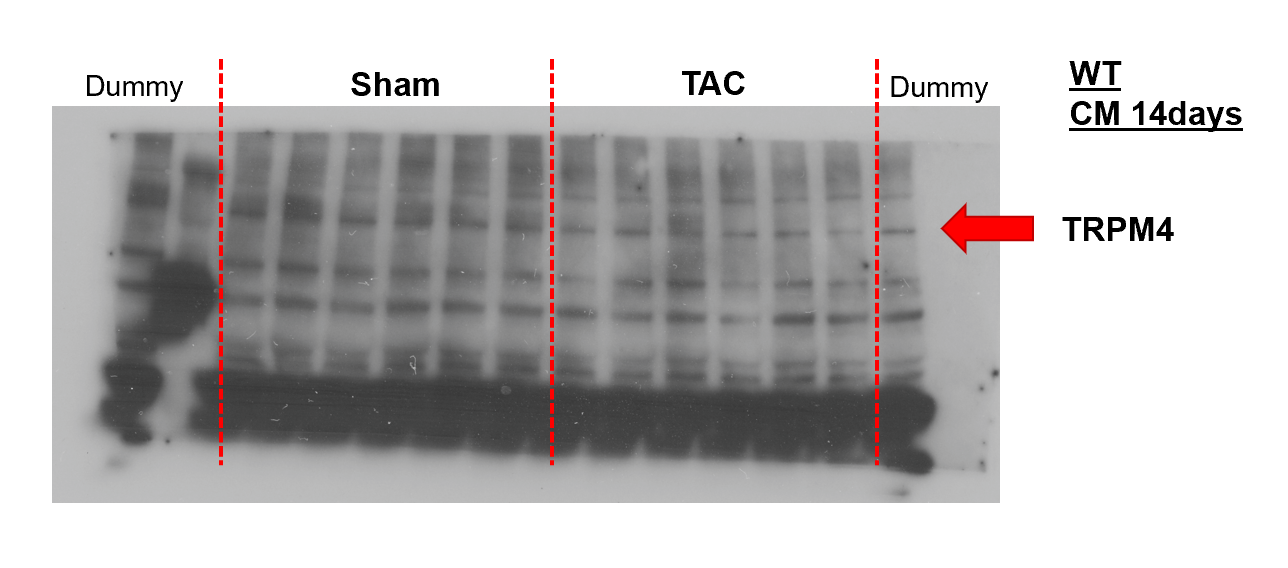

Supplement: Source data 1. [file elife-66582-data1.zip › Blots and Blot Figs/Blot Figs/WT TRPM4/WT CM 14days TRPM4 Fig.tif]

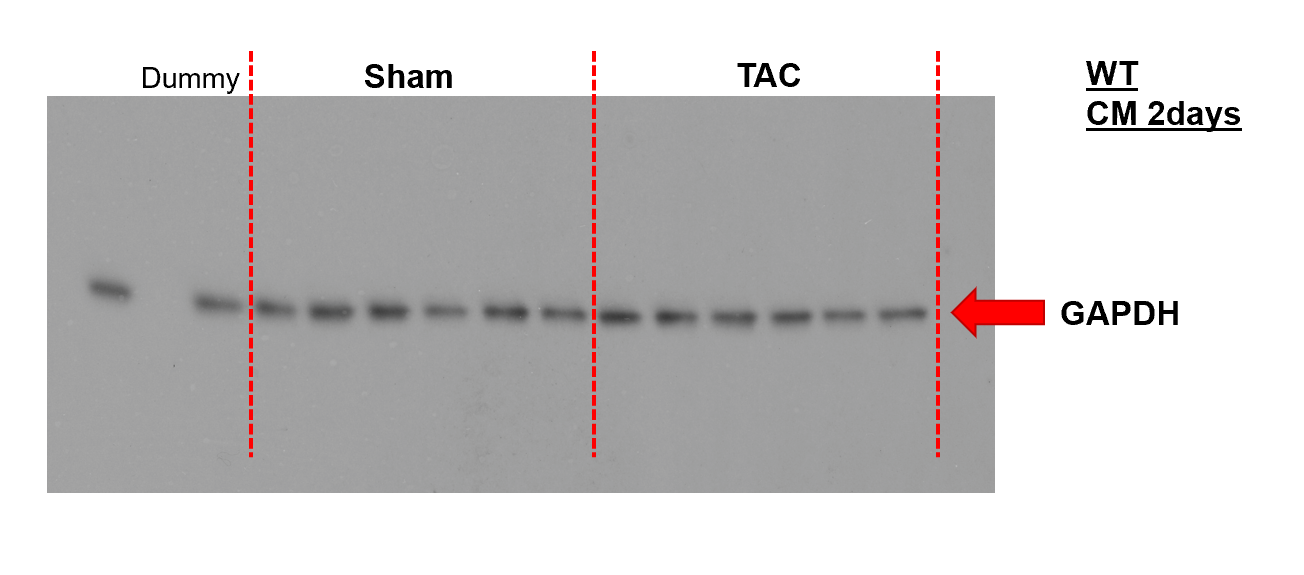

Supplement: Source data 1. [file elife-66582-data1.zip › Blots and Blot Figs/Blot Figs/WT TRPM4/WT CM 2days GAPDH Fig.tif]

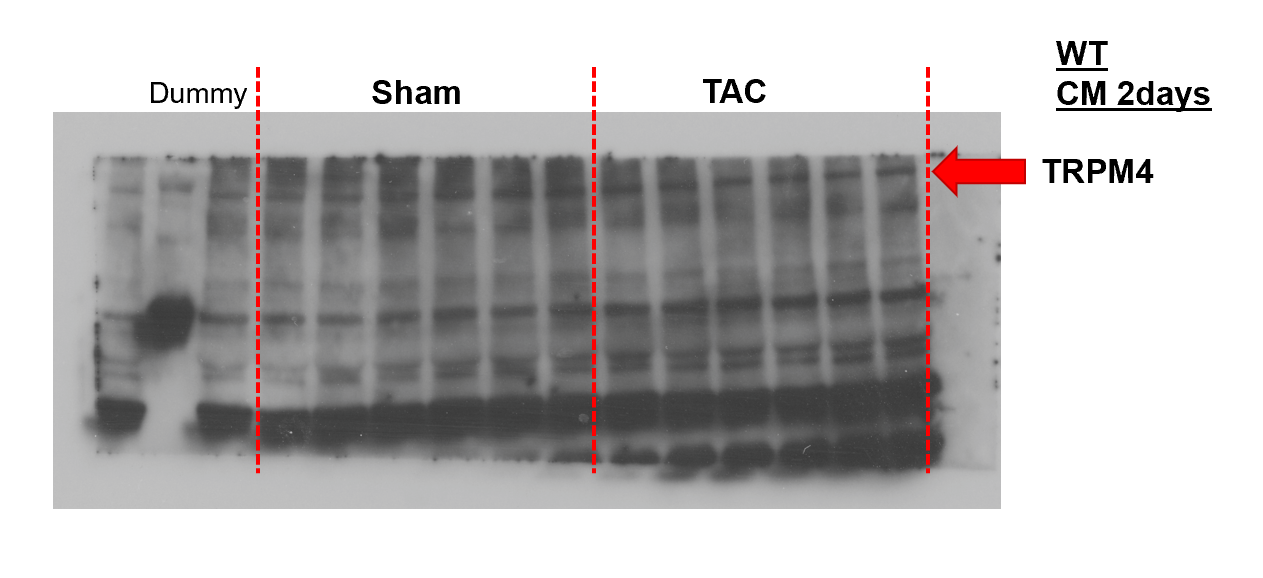

Supplement: Source data 1. [file elife-66582-data1.zip › Blots and Blot Figs/Blot Figs/WT TRPM4/WT CM 2days TRPM4 Fig.tif]

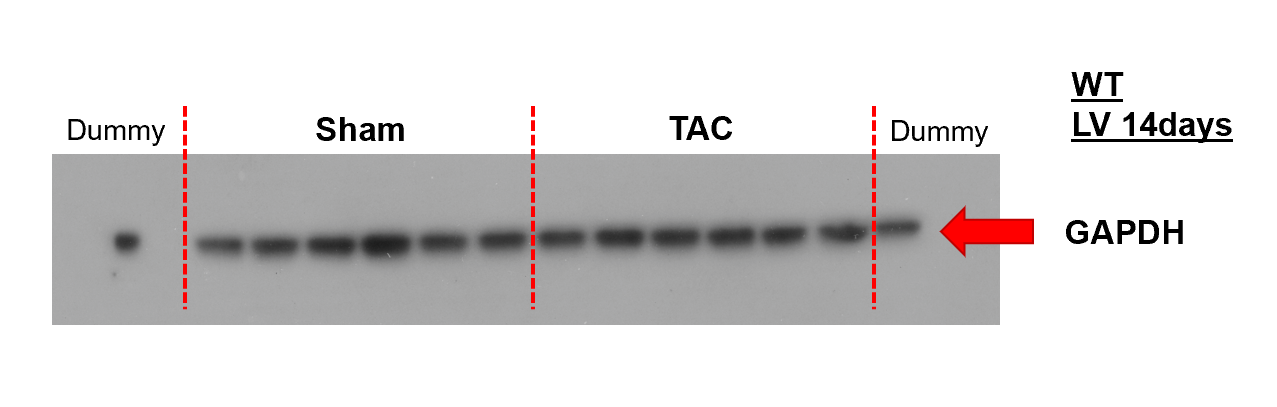

Supplement: Source data 1. [file elife-66582-data1.zip › Blots and Blot Figs/Blot Figs/WT TRPM4/WT LV 14days GAPDH Fig.tif]

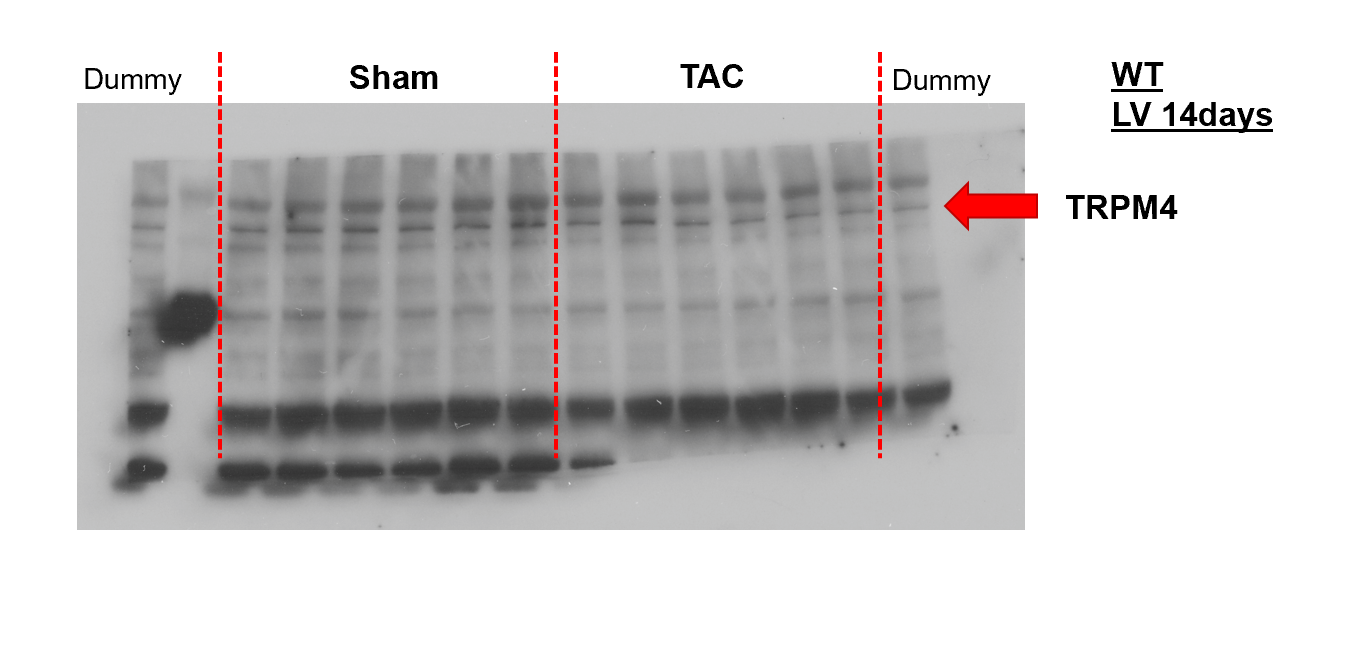

Supplement: Source data 1. [file elife-66582-data1.zip › Blots and Blot Figs/Blot Figs/WT TRPM4/WT LV 14days TRPM4 Fig.tif]

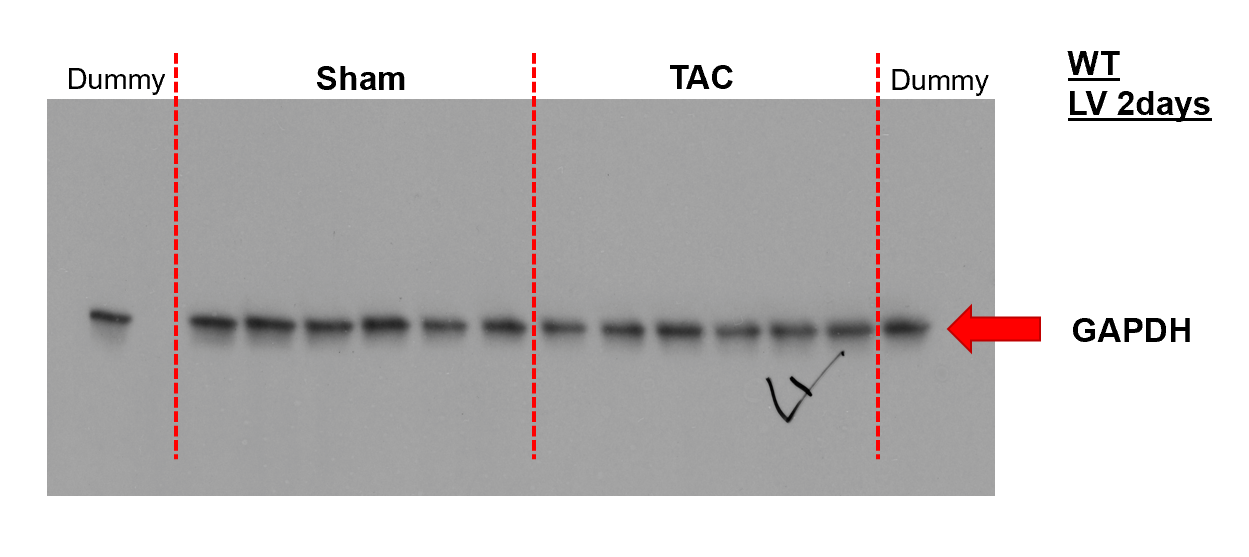

Supplement: Source data 1. [file elife-66582-data1.zip › Blots and Blot Figs/Blot Figs/WT TRPM4/WT LV 2days GAPDH Fig.tif]

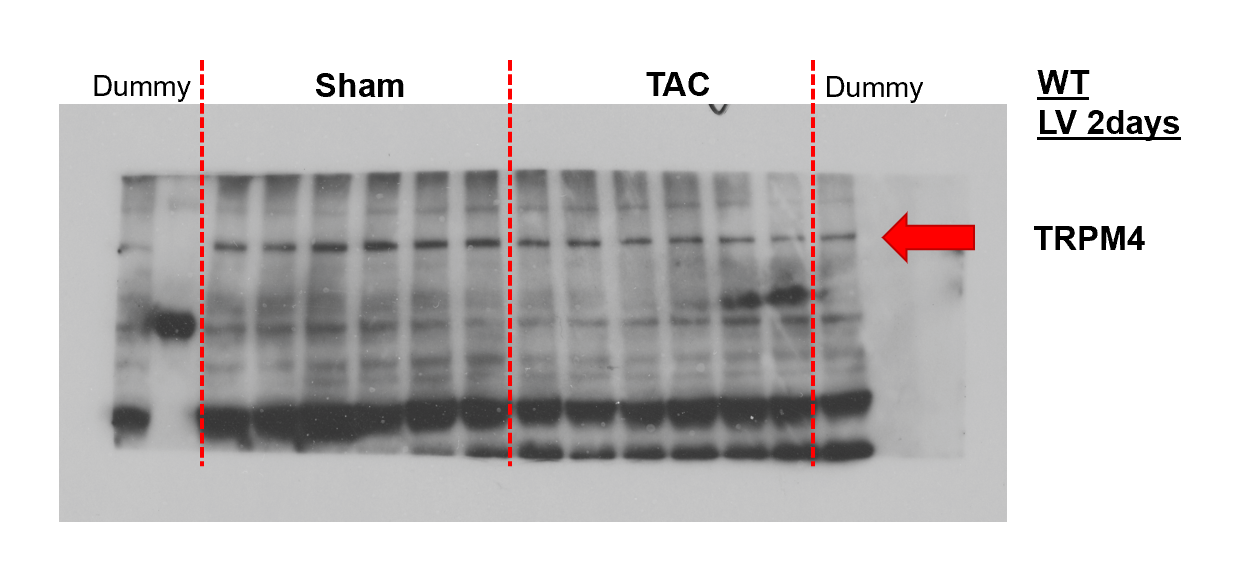

Supplement: Source data 1. [file elife-66582-data1.zip › Blots and Blot Figs/Blot Figs/WT TRPM4/WT LV 2days TRPM4 Fig.tif]

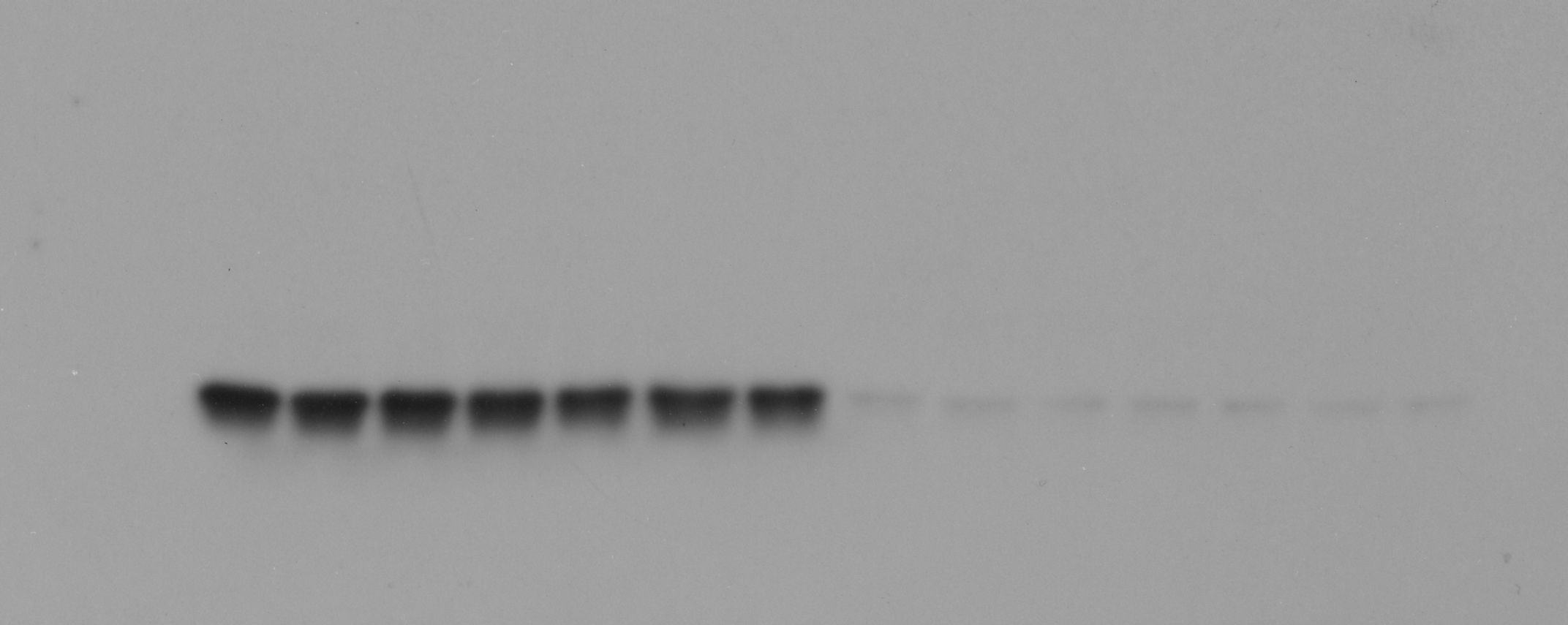

Supplement: Source data 1. [file elife-66582-data1.zip › Blots and Blot Figs/Blots/Cyto_Nu fraction/GAPDH.jpg]

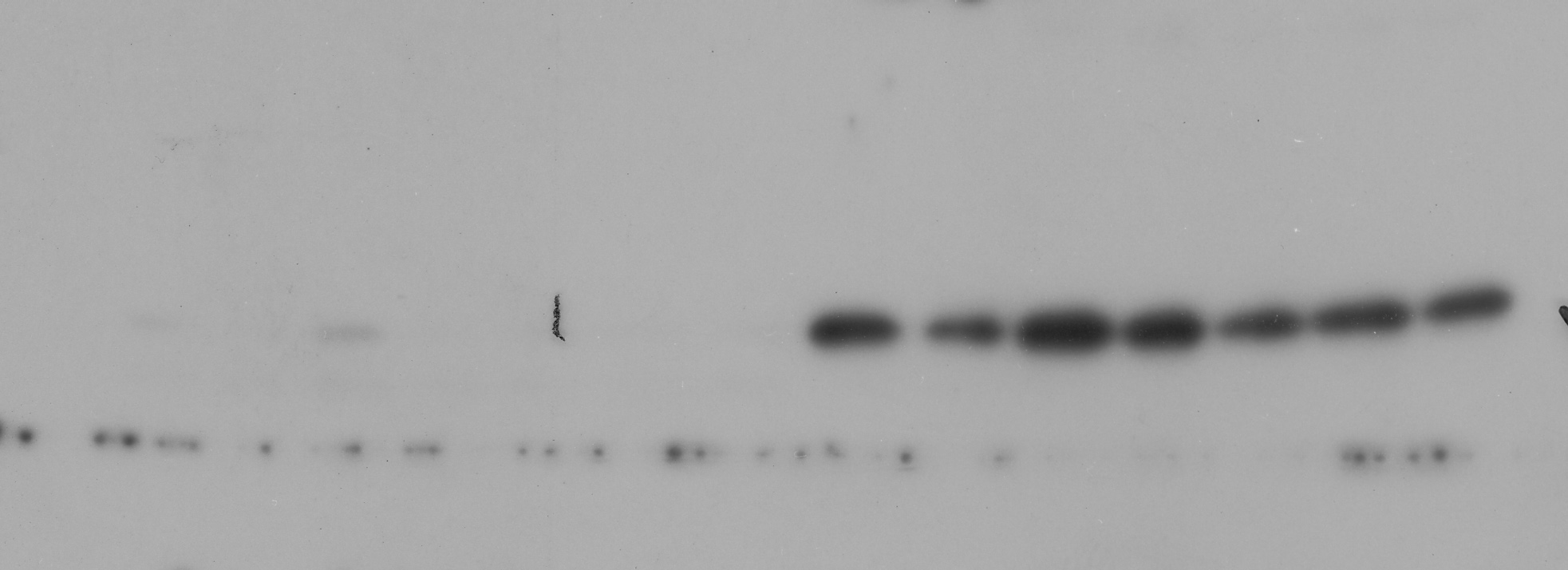

Supplement: Source data 1. [file elife-66582-data1.zip › Blots and Blot Figs/Blots/Cyto_Nu fraction/Histone.jpg]

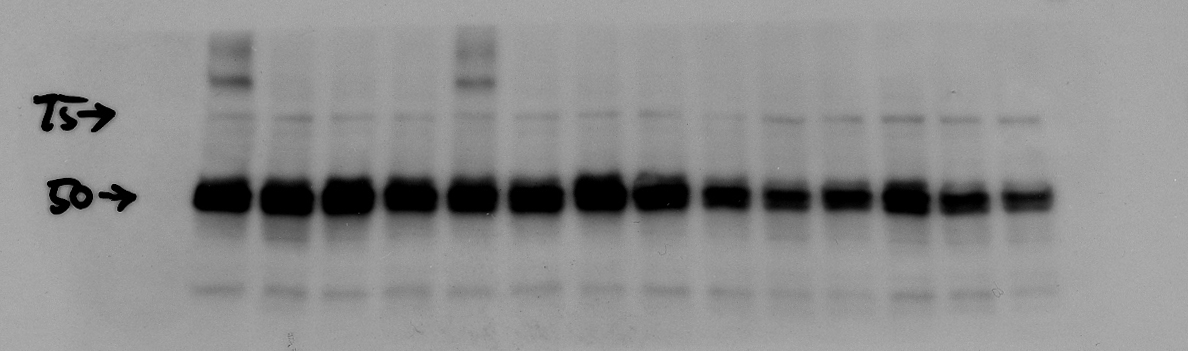

Supplement: Source data 1. [file elife-66582-data1.zip › Blots and Blot Figs/Blots/KO Cyto/KO Cyto CaMK2.JPG]

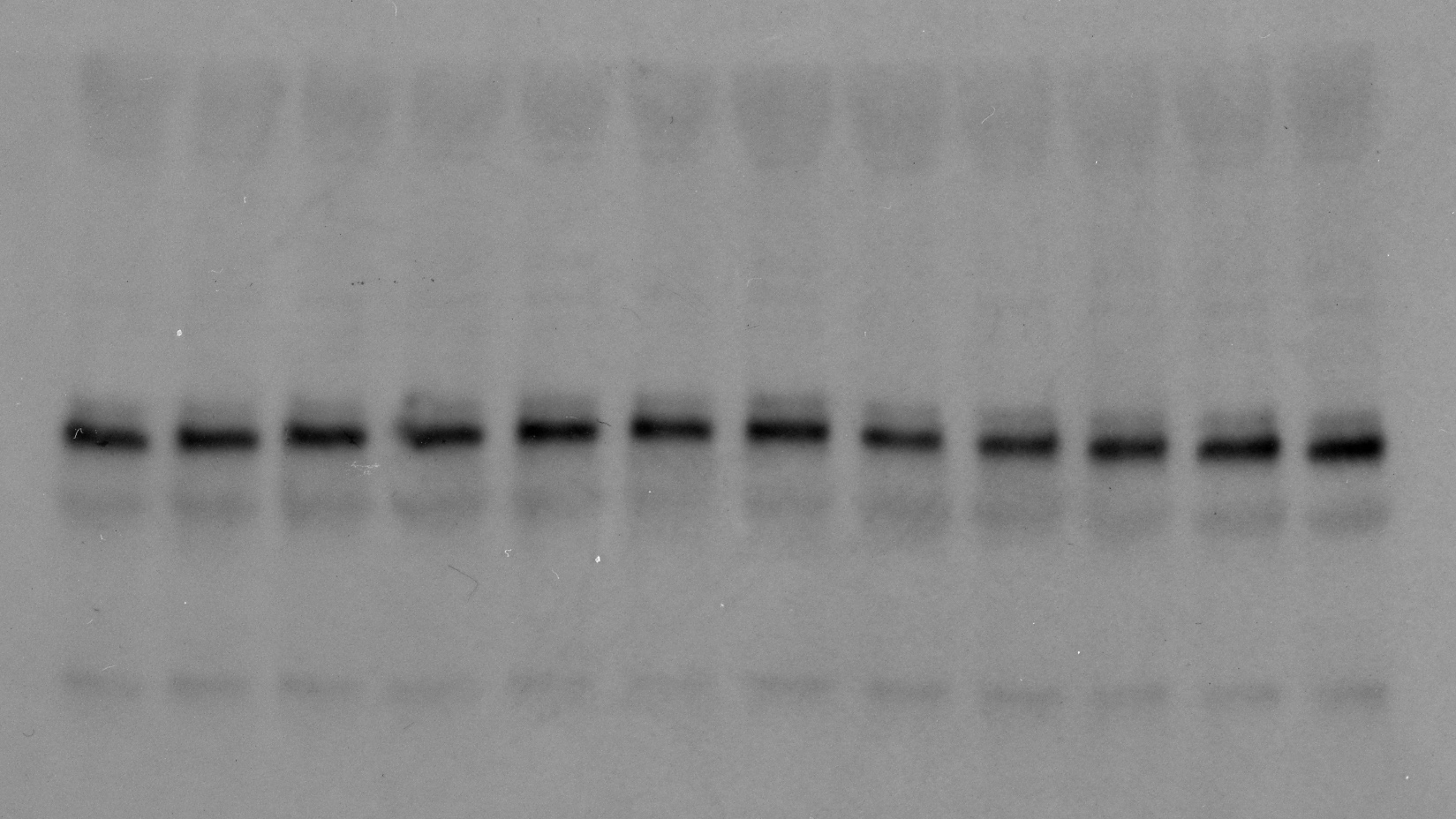

Supplement: Source data 1. [file elife-66582-data1.zip › Blots and Blot Figs/Blots/KO Cyto/KO Cyto GSK3b.jpg]

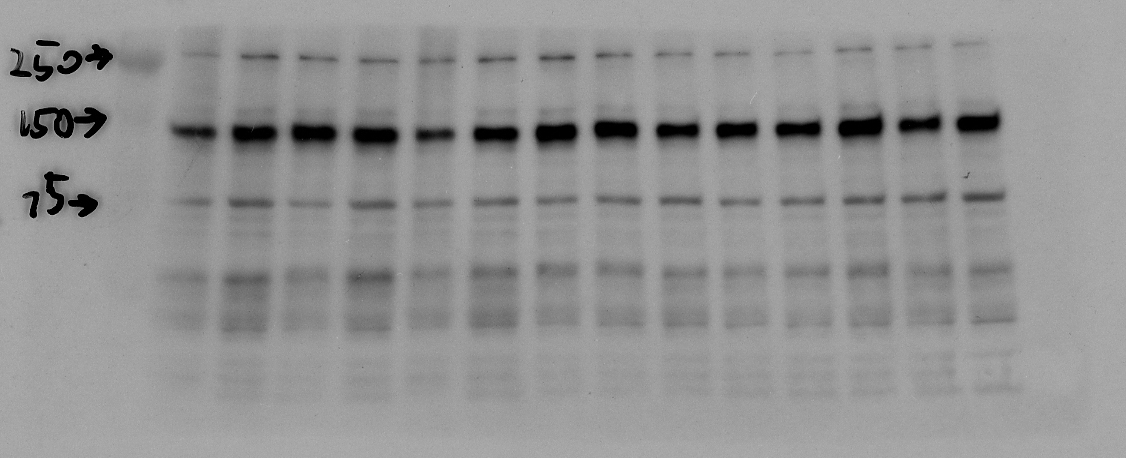

Supplement: Source data 1. [file elife-66582-data1.zip › Blots and Blot Figs/Blots/KO Cyto/KO Cyto HDAC4.jpg]

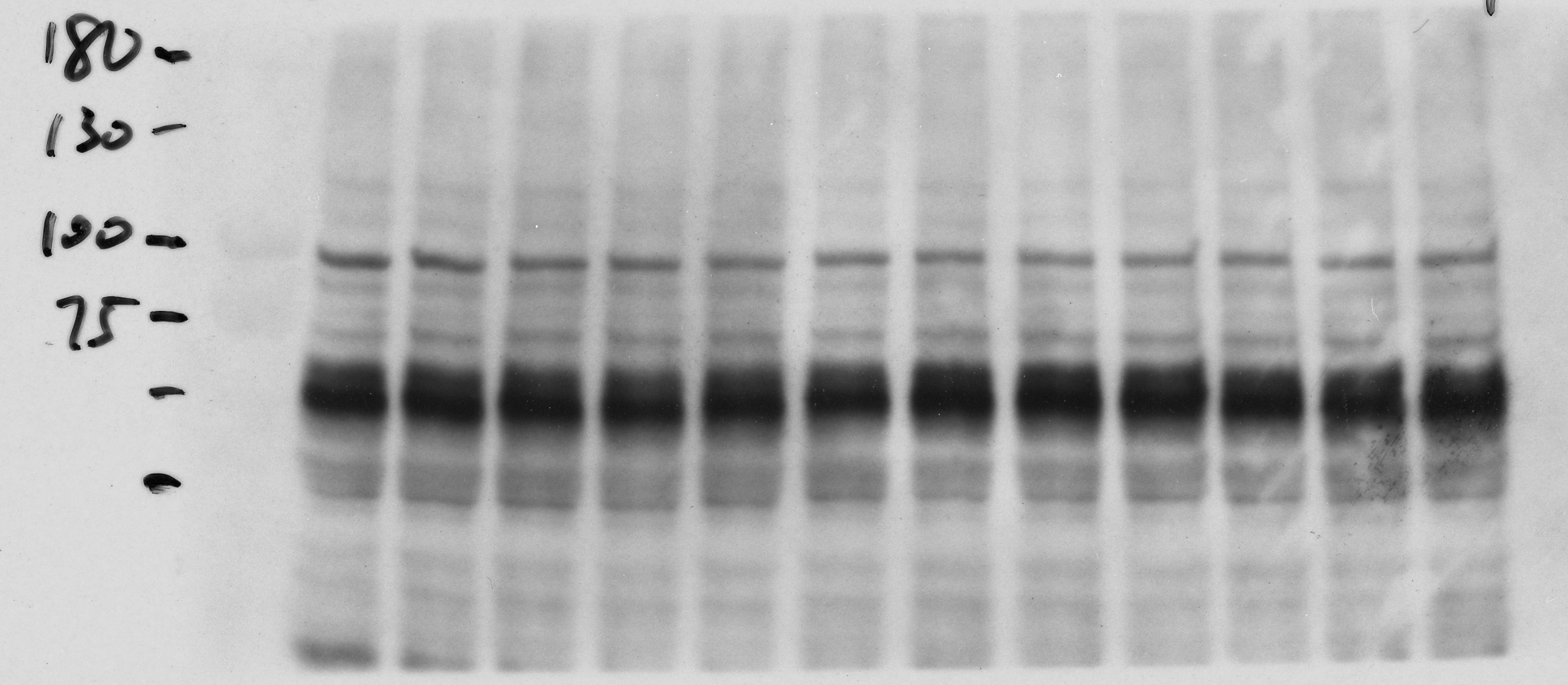

Supplement: Source data 1. [file elife-66582-data1.zip › Blots and Blot Figs/Blots/KO Cyto/KO Cyto NFAT.jpg]

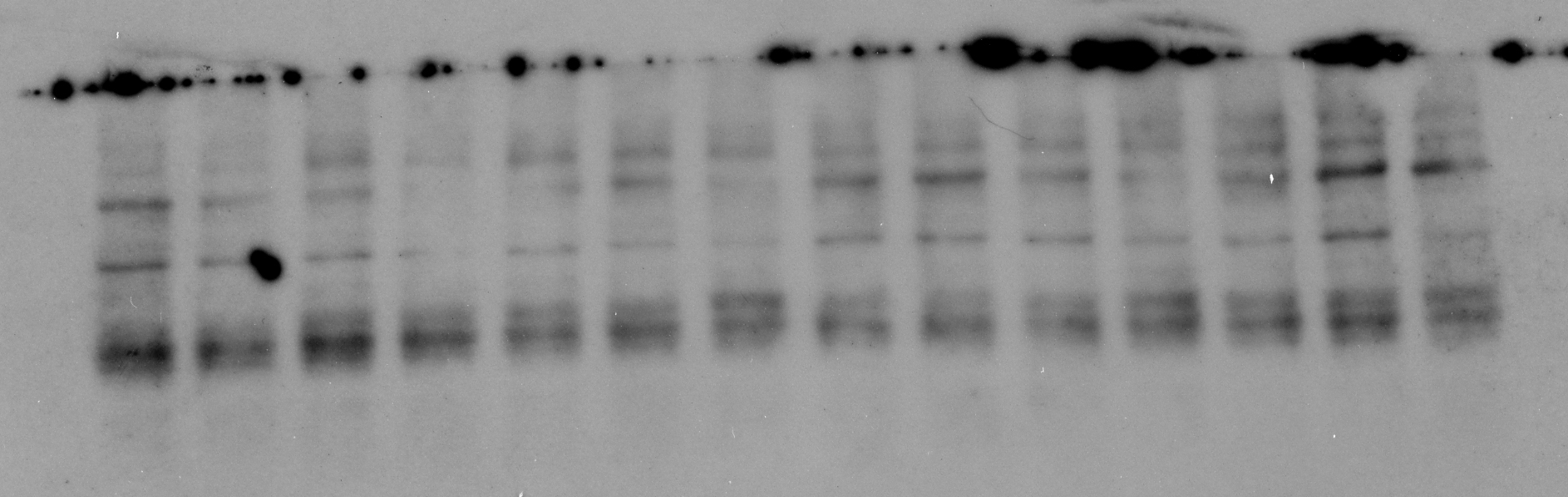

Supplement: Source data 1. [file elife-66582-data1.zip › Blots and Blot Figs/Blots/KO Cyto/KO Cyto p-CaMK2.jpg]

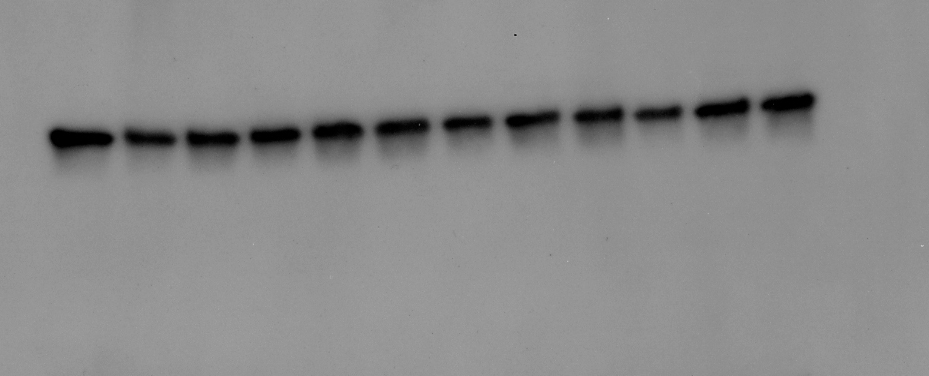

Supplement: Source data 1. [file elife-66582-data1.zip › Blots and Blot Figs/Blots/KO Cyto/KO Cyto p-GSK3b.jpg]

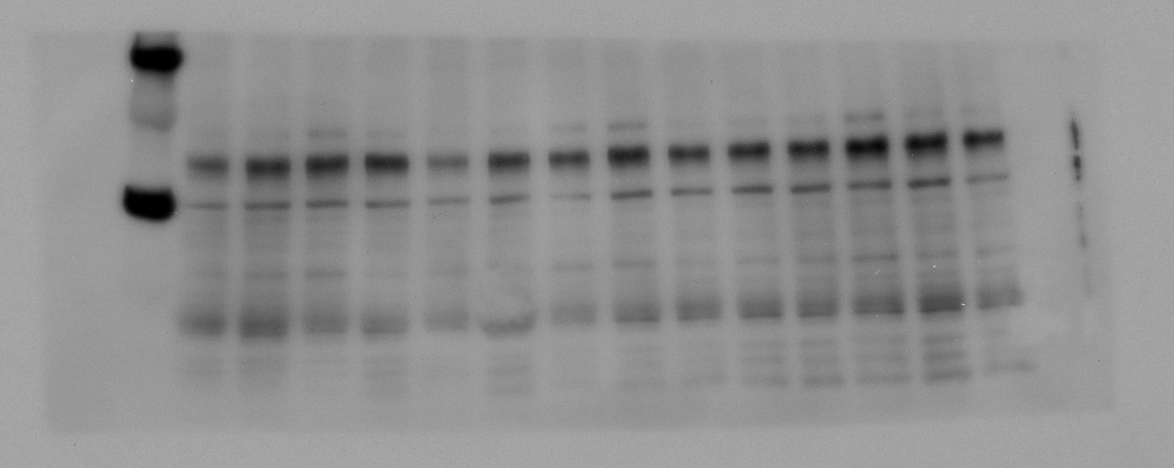

Supplement: Source data 1. [file elife-66582-data1.zip › Blots and Blot Figs/Blots/KO Cyto/KO Cyto p-HDAC4.jpg]

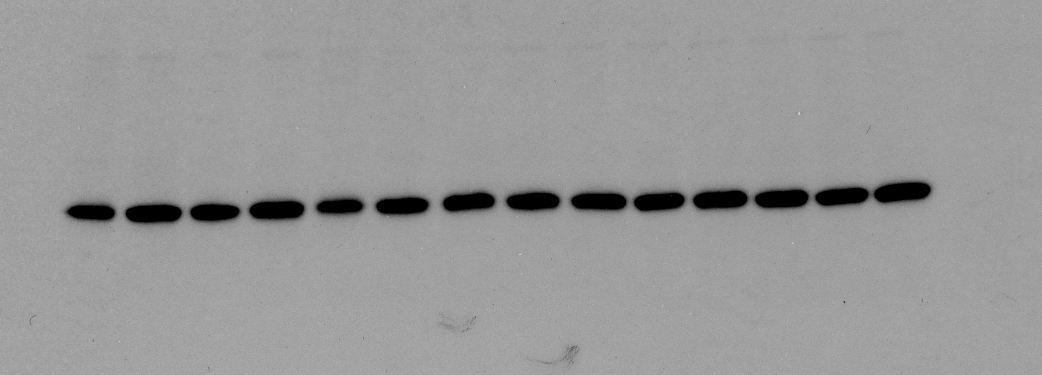

Supplement: Source data 1. [file elife-66582-data1.zip › Blots and Blot Figs/Blots/KO Cyto/KO GAPDH1.jpg]

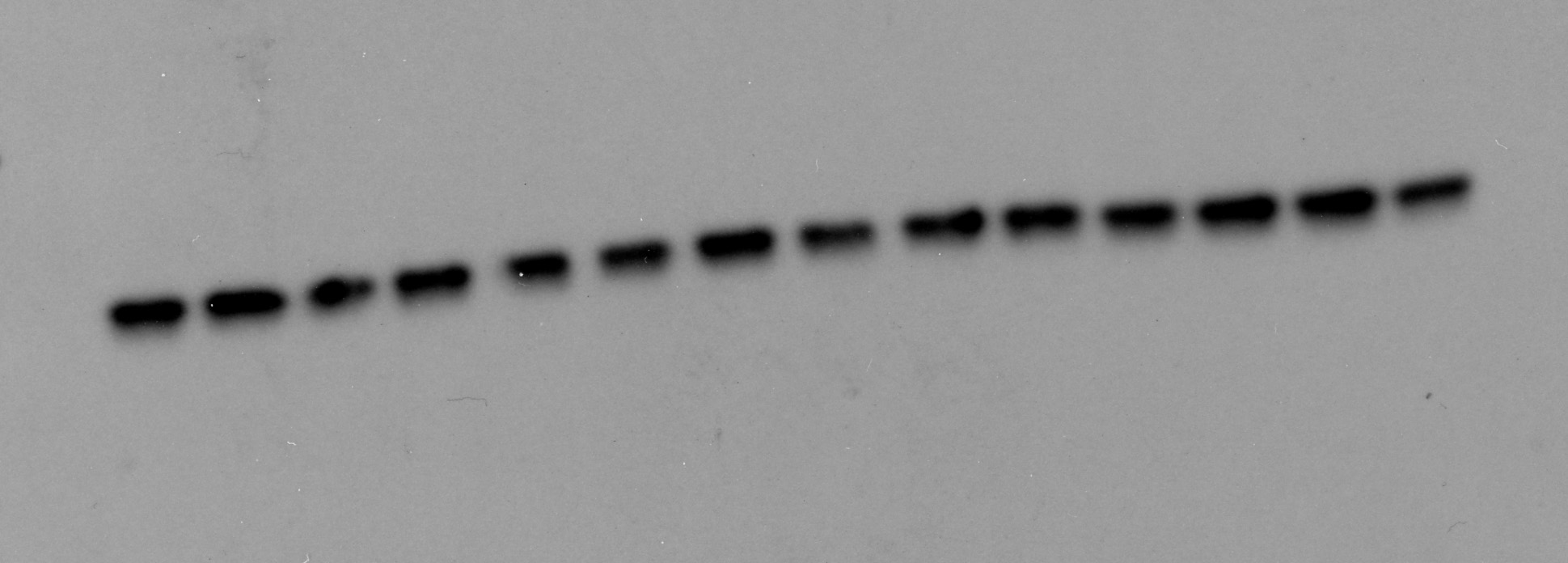

Supplement: Source data 1. [file elife-66582-data1.zip › Blots and Blot Figs/Blots/KO Cyto/KO GAPDH2.jpg]

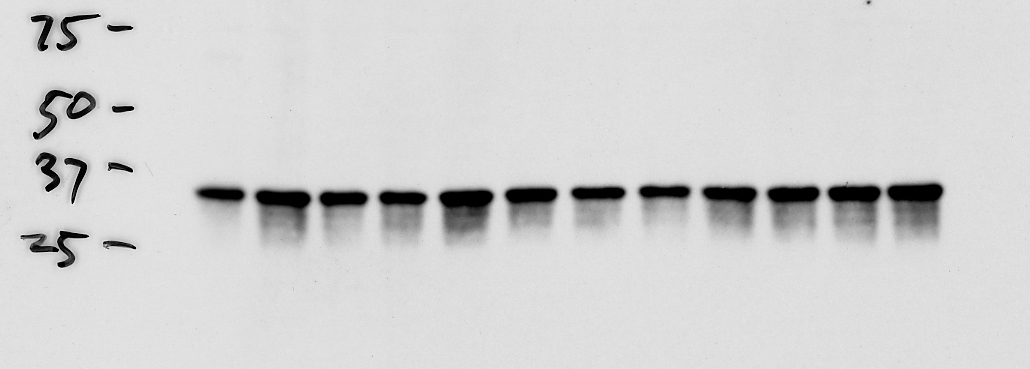

Supplement: Source data 1. [file elife-66582-data1.zip › Blots and Blot Figs/Blots/KO Cyto/KO GAPDH3.jpg]

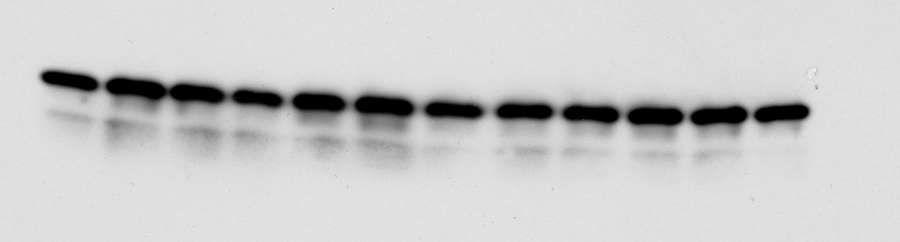

Supplement: Source data 1. [file elife-66582-data1.zip › Blots and Blot Figs/Blots/KO Cyto/KO GAPDH4.jpg]

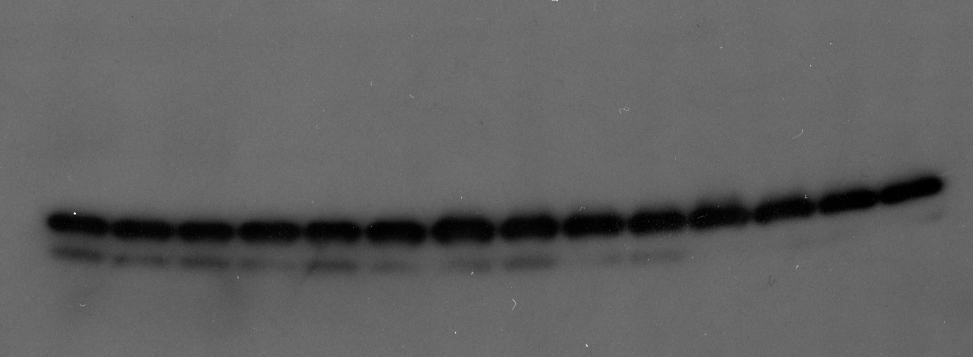

Supplement: Source data 1. [file elife-66582-data1.zip › Blots and Blot Figs/Blots/KO Nu/KO Histone H2B1.jpg]

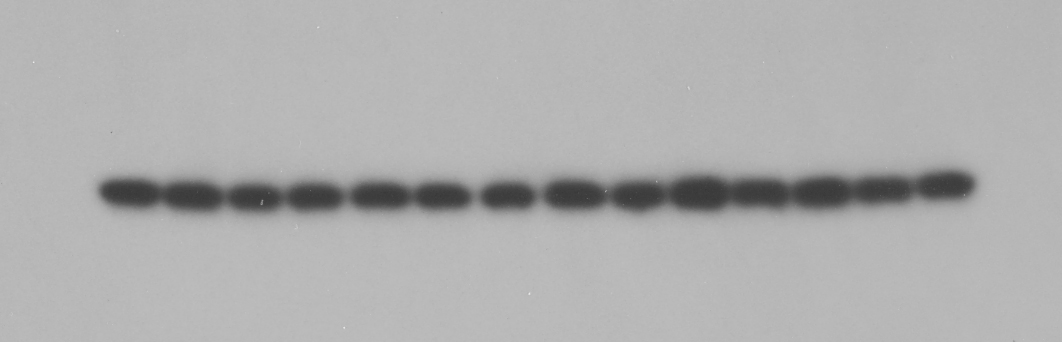

Supplement: Source data 1. [file elife-66582-data1.zip › Blots and Blot Figs/Blots/KO Nu/KO Histone H2B2.jpg]

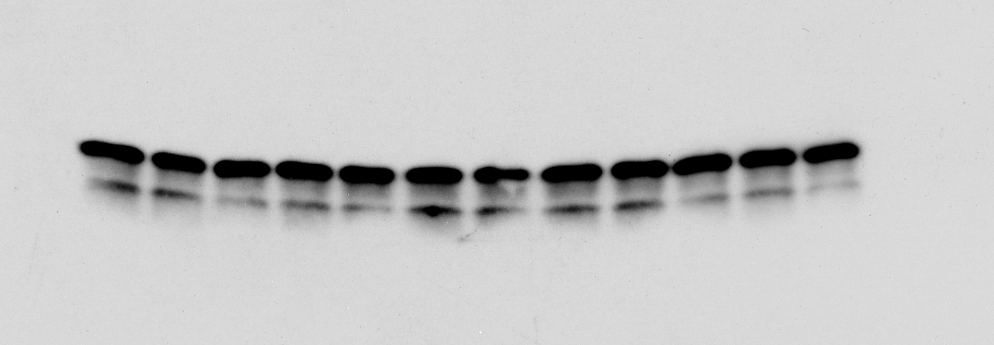

Supplement: Source data 1. [file elife-66582-data1.zip › Blots and Blot Figs/Blots/KO Nu/KO Histone H2B3.jpg]

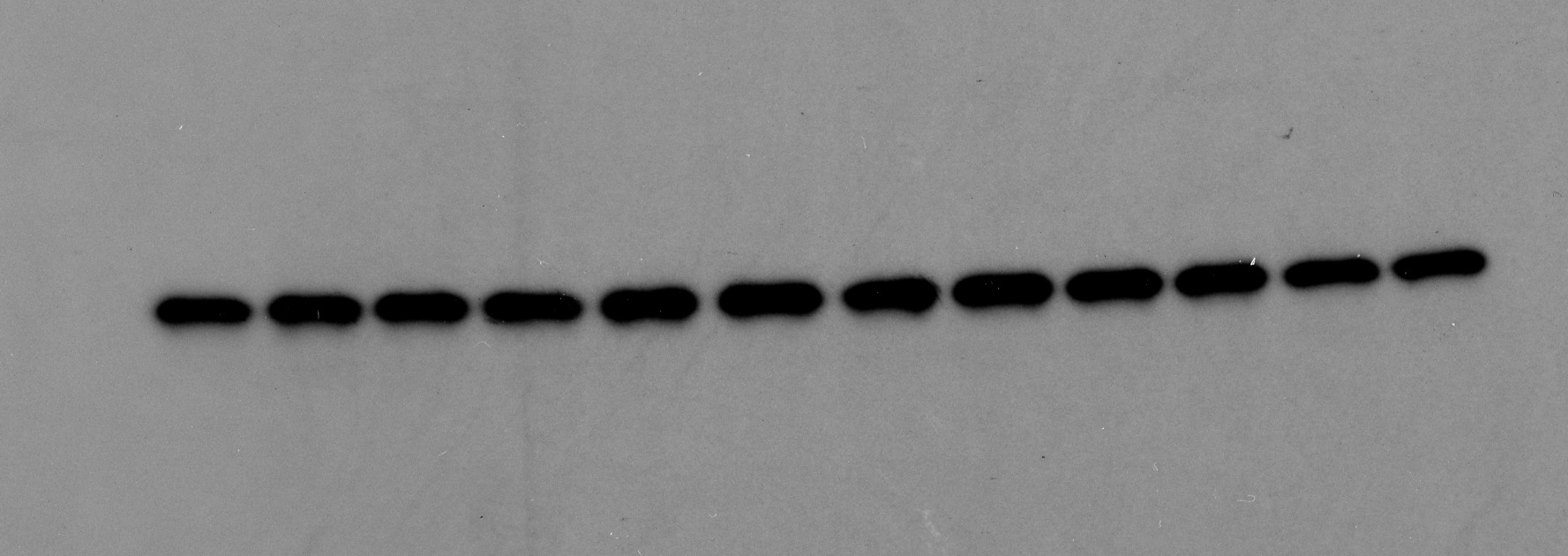

Supplement: Source data 1. [file elife-66582-data1.zip › Blots and Blot Figs/Blots/KO Nu/KO Histone H2B4.jpg]

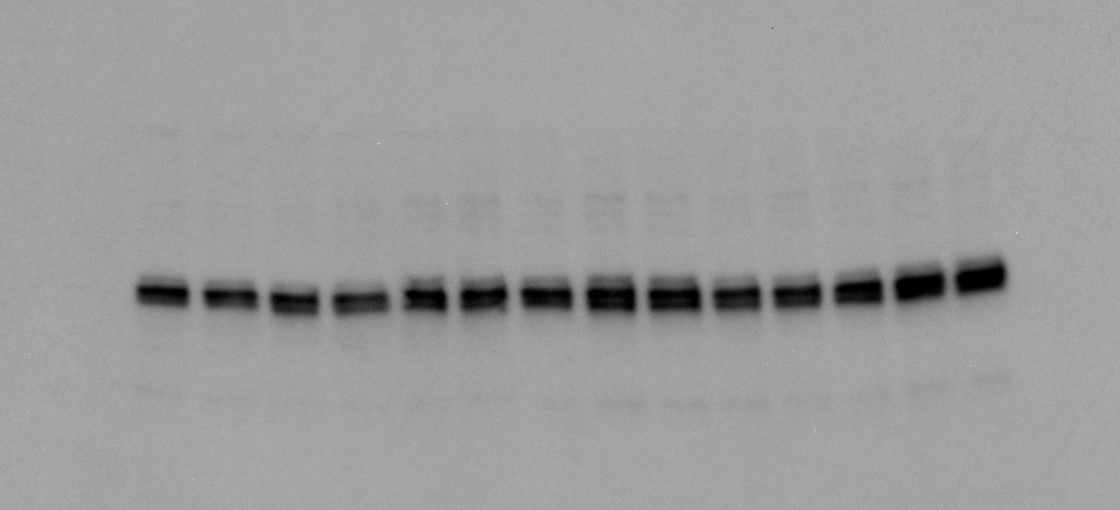

Supplement: Source data 1. [file elife-66582-data1.zip › Blots and Blot Figs/Blots/KO Nu/KO Nu CaMK2.jpg]

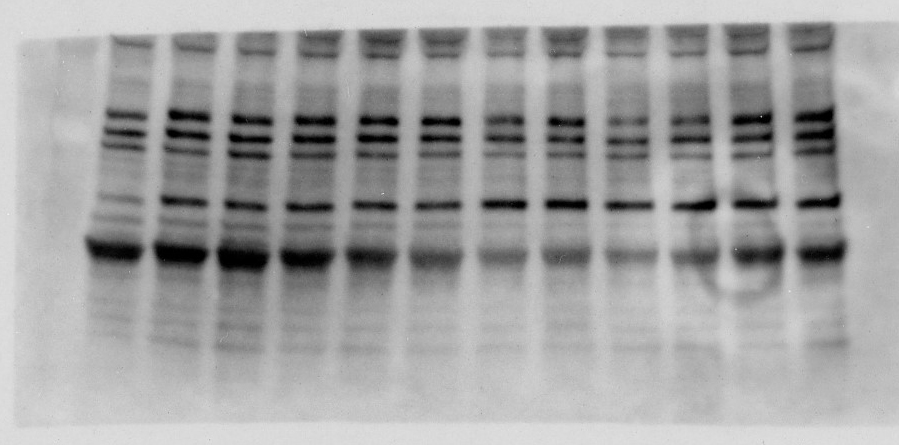

Supplement: Source data 1. [file elife-66582-data1.zip › Blots and Blot Figs/Blots/KO Nu/KO Nu GATA4.jpg]

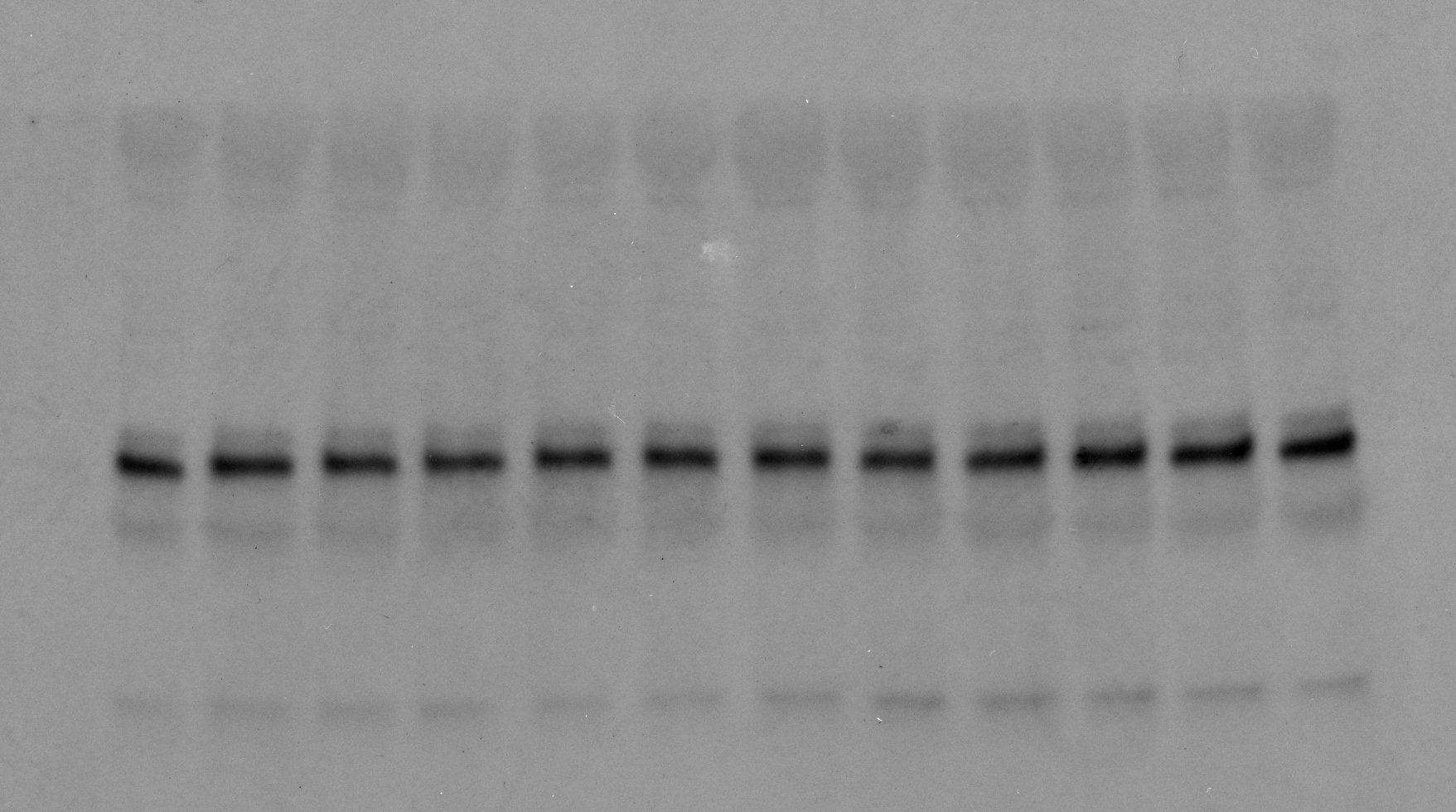

Supplement: Source data 1. [file elife-66582-data1.zip › Blots and Blot Figs/Blots/KO Nu/KO Nu GSK3b.jpg]

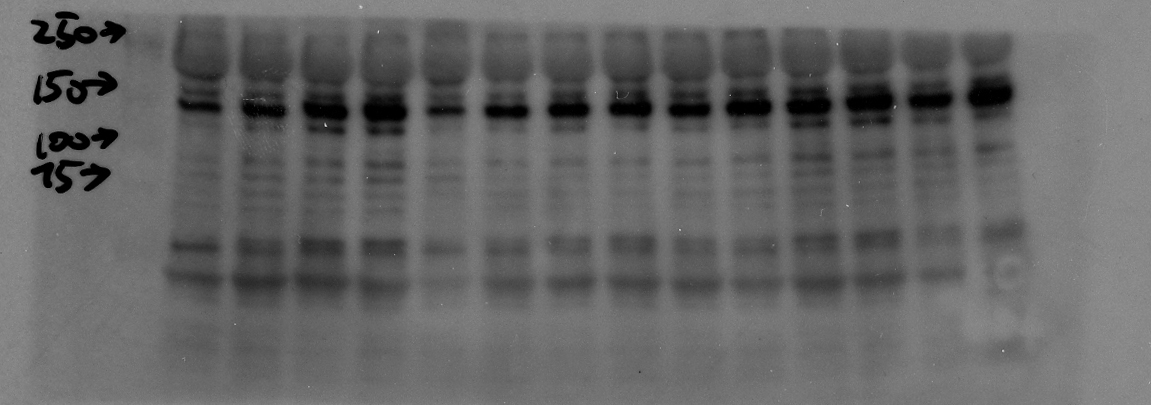

Supplement: Source data 1. [file elife-66582-data1.zip › Blots and Blot Figs/Blots/KO Nu/KO Nu HDAC4.jpg]

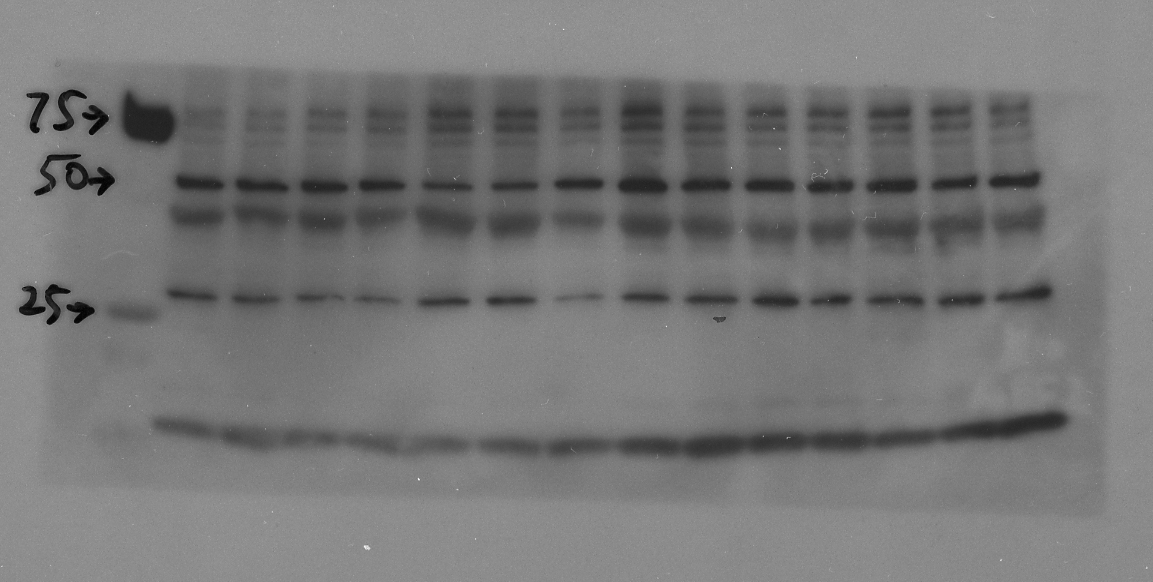

Supplement: Source data 1. [file elife-66582-data1.zip › Blots and Blot Figs/Blots/KO Nu/KO Nu MEF2.jpg]

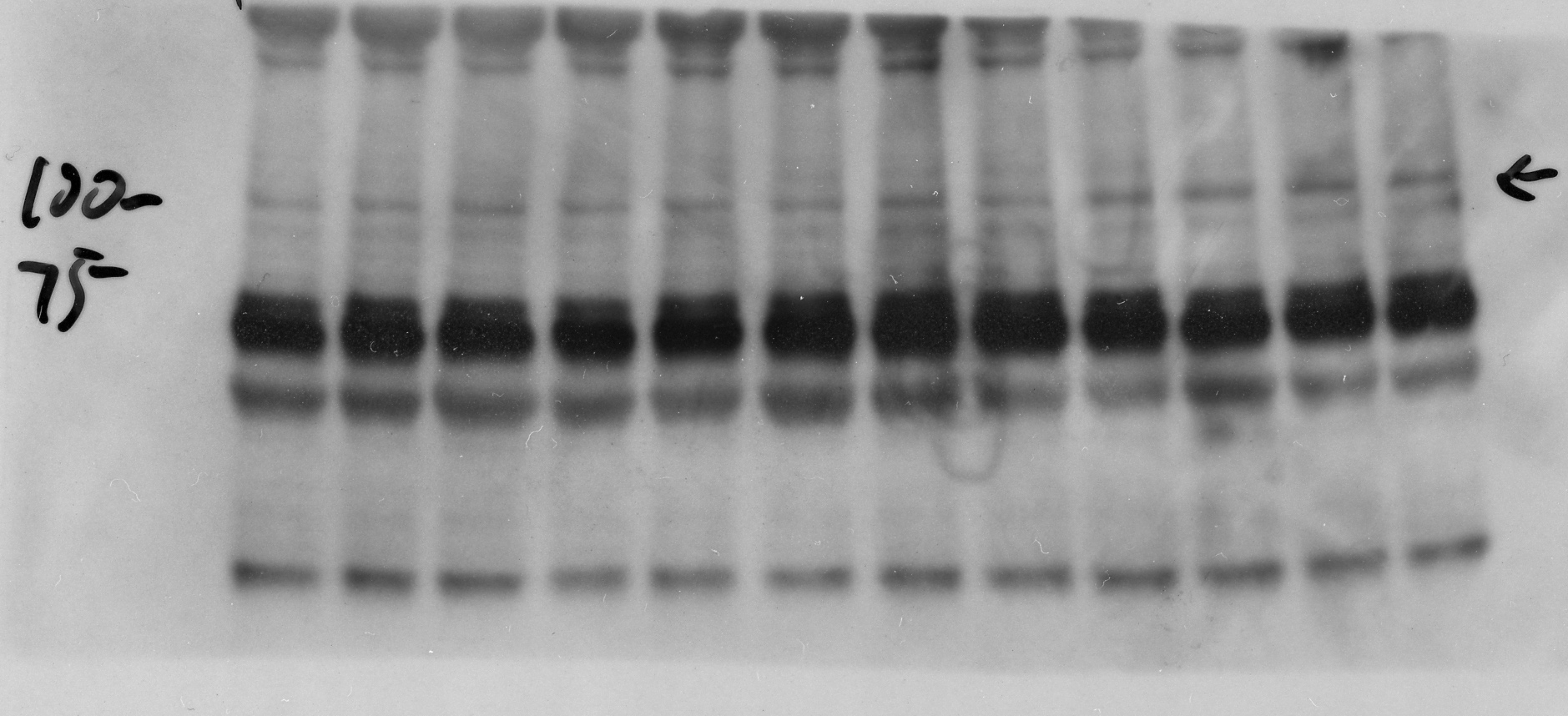

Supplement: Source data 1. [file elife-66582-data1.zip › Blots and Blot Figs/Blots/KO Nu/KO Nu NFAT.jpg]

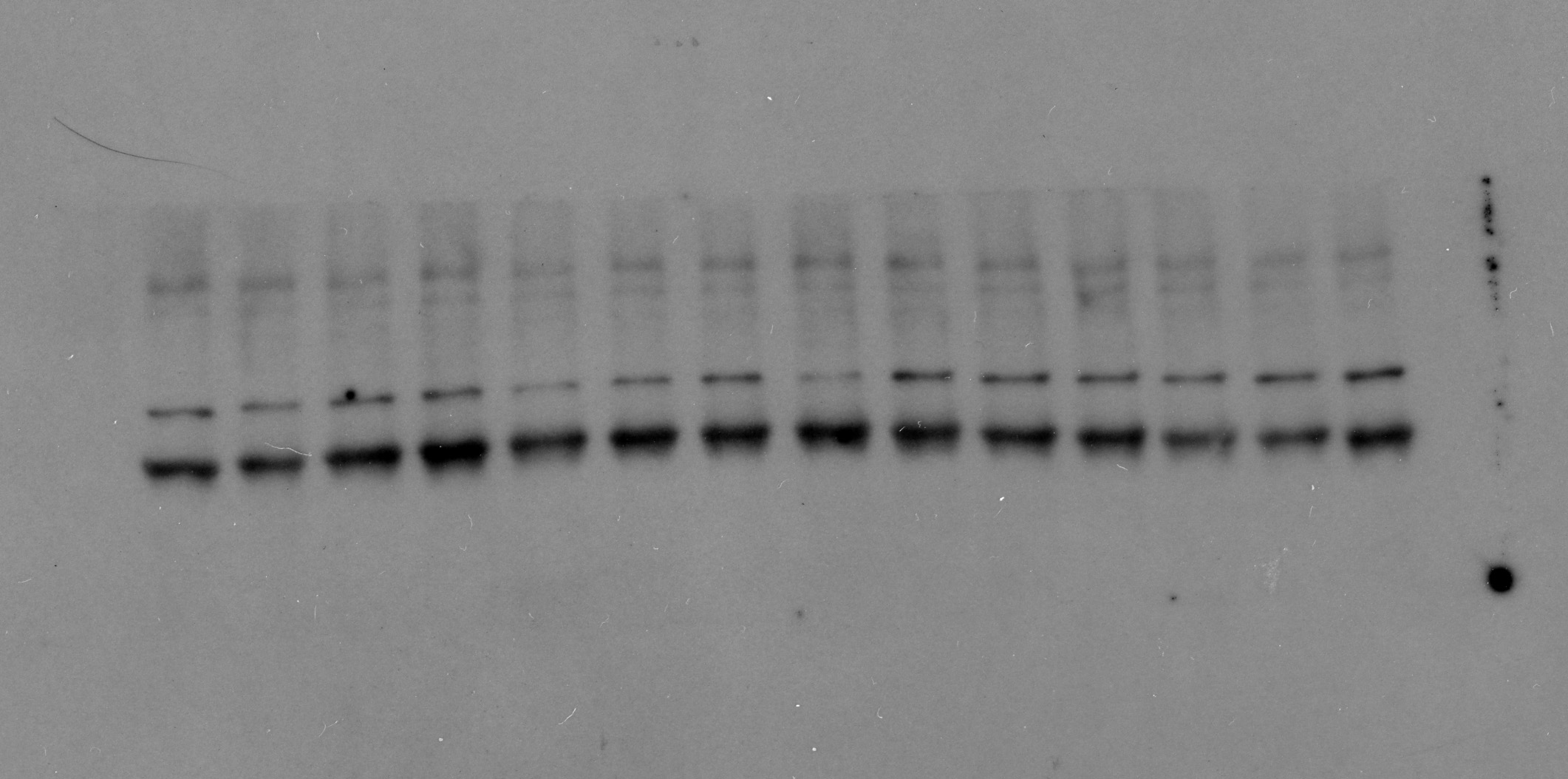

Supplement: Source data 1. [file elife-66582-data1.zip › Blots and Blot Figs/Blots/KO Nu/KO Nu p-CaMK2.jpg]

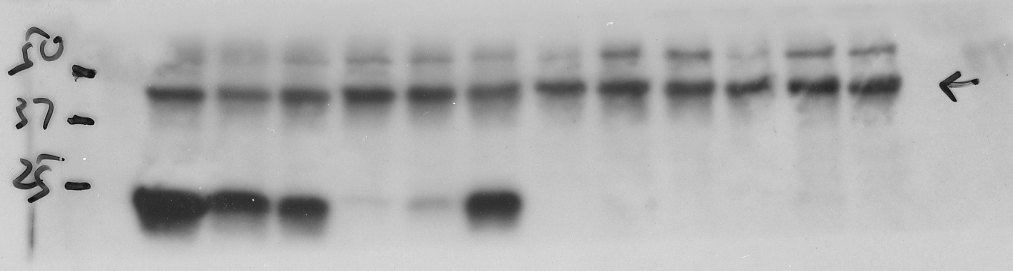

Supplement: Source data 1. [file elife-66582-data1.zip › Blots and Blot Figs/Blots/KO Nu/KO Nu p-GSK3b.jpg]

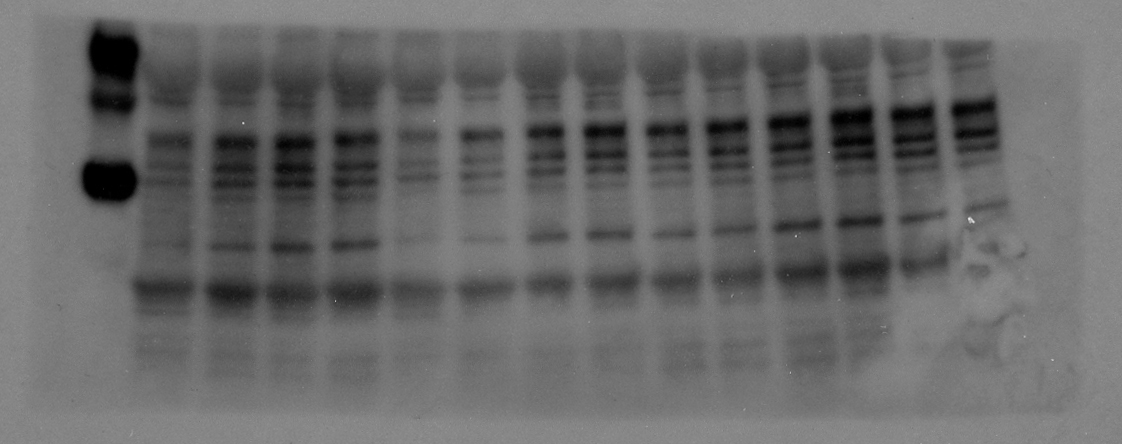

Supplement: Source data 1. [file elife-66582-data1.zip › Blots and Blot Figs/Blots/KO Nu/KO Nu p-HDAC4.jpg]

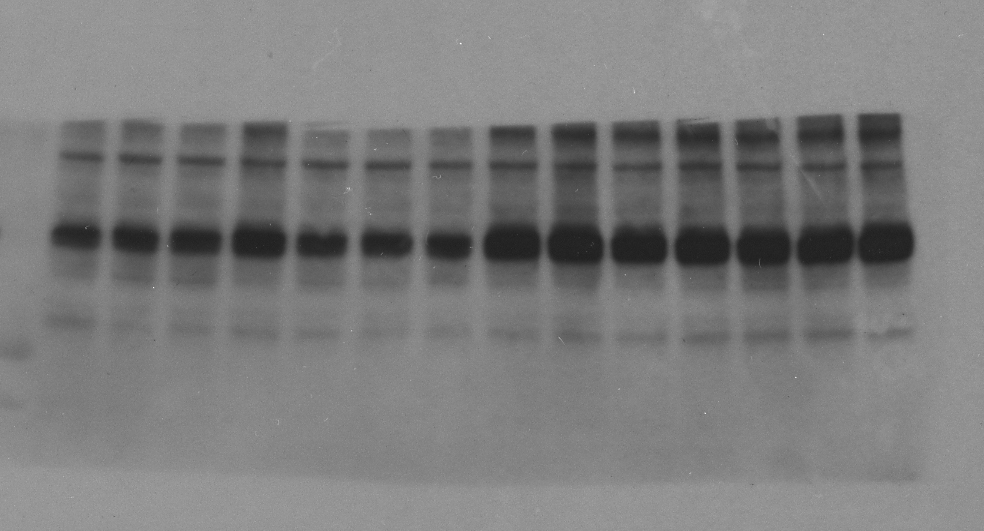

Supplement: Source data 1. [file elife-66582-data1.zip › Blots and Blot Figs/Blots/WT Cyto/WT Cyto CaMK2.jpg]

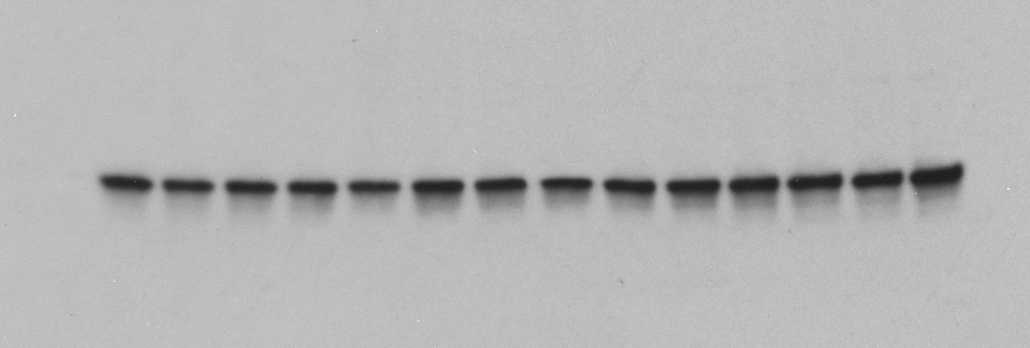

Supplement: Source data 1. [file elife-66582-data1.zip › Blots and Blot Figs/Blots/WT Cyto/WT Cyto GSK3b.jpg]

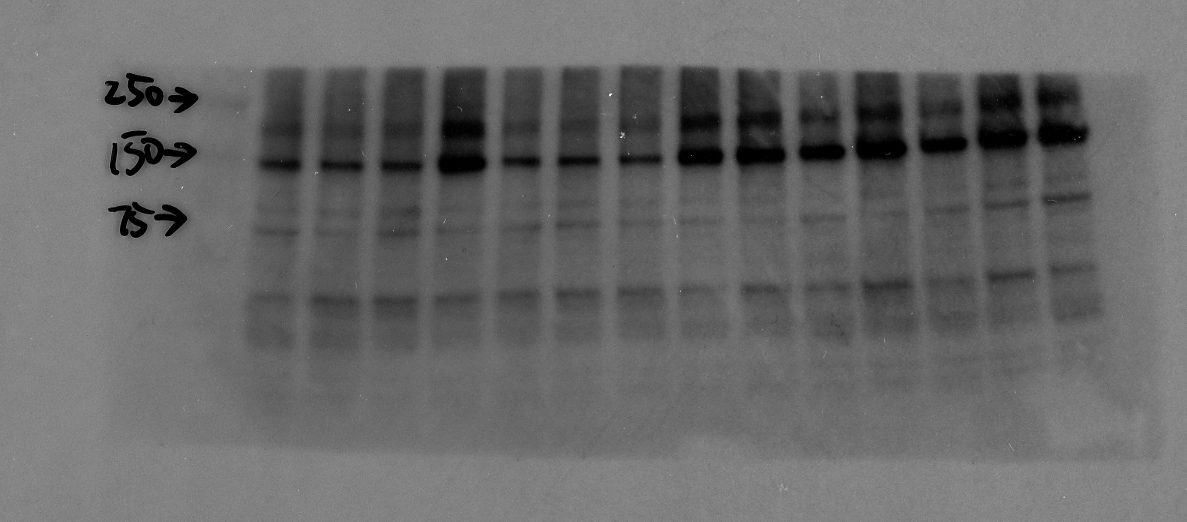

Supplement: Source data 1. [file elife-66582-data1.zip › Blots and Blot Figs/Blots/WT Cyto/WT Cyto HDAC4.jpg]

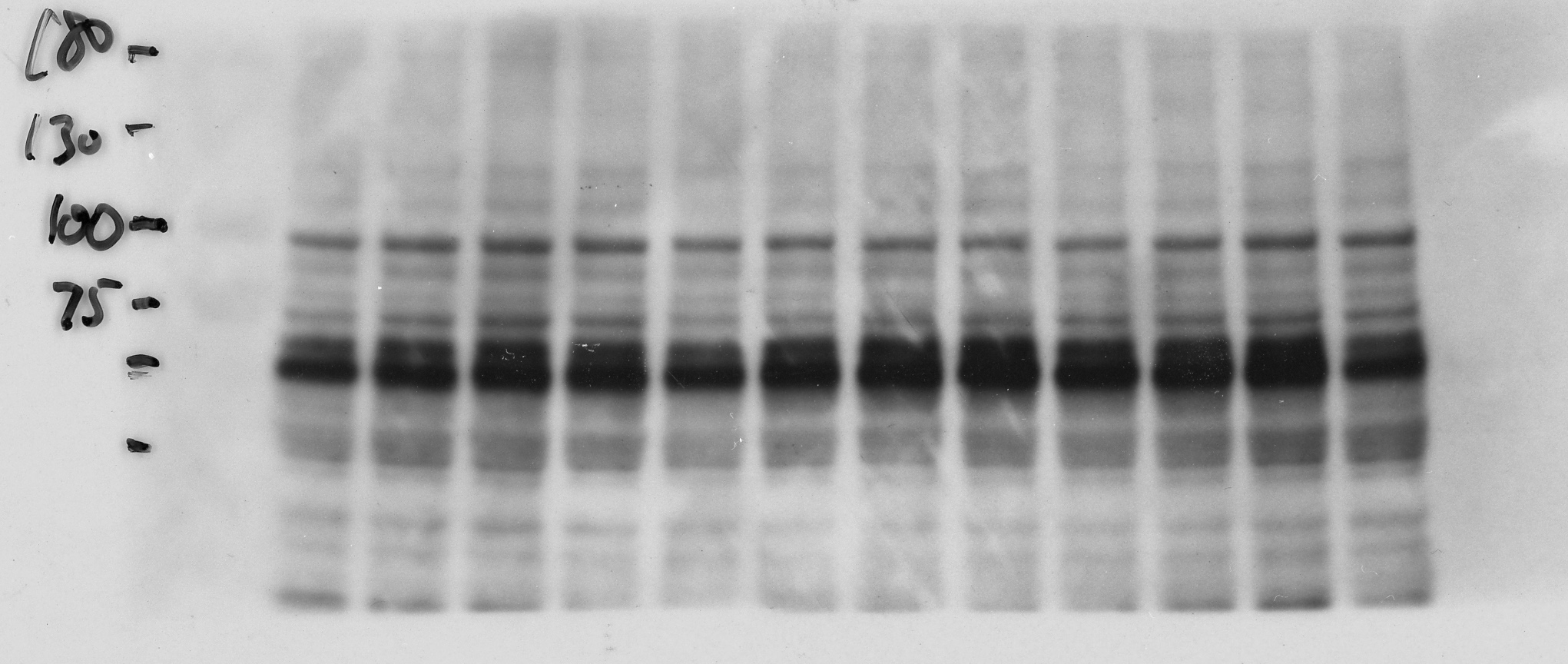

Supplement: Source data 1. [file elife-66582-data1.zip › Blots and Blot Figs/Blots/WT Cyto/WT Cyto NFAT.jpg]

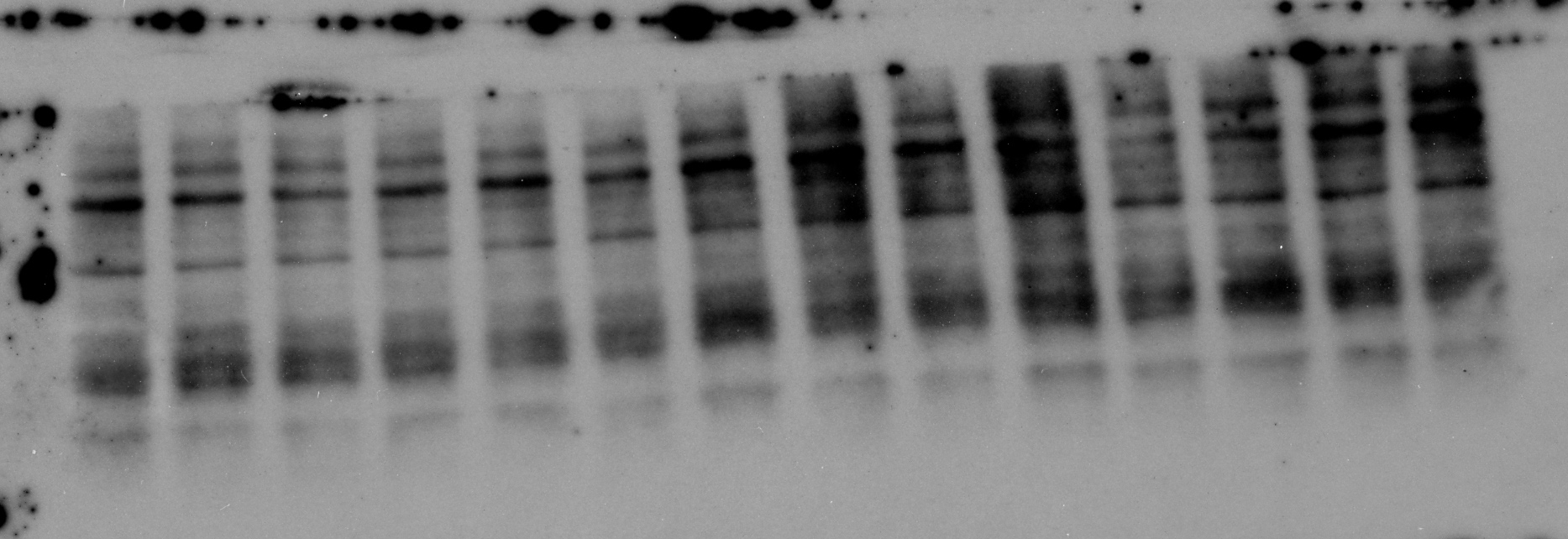

Supplement: Source data 1. [file elife-66582-data1.zip › Blots and Blot Figs/Blots/WT Cyto/WT Cyto p-CaMK2.jpg]

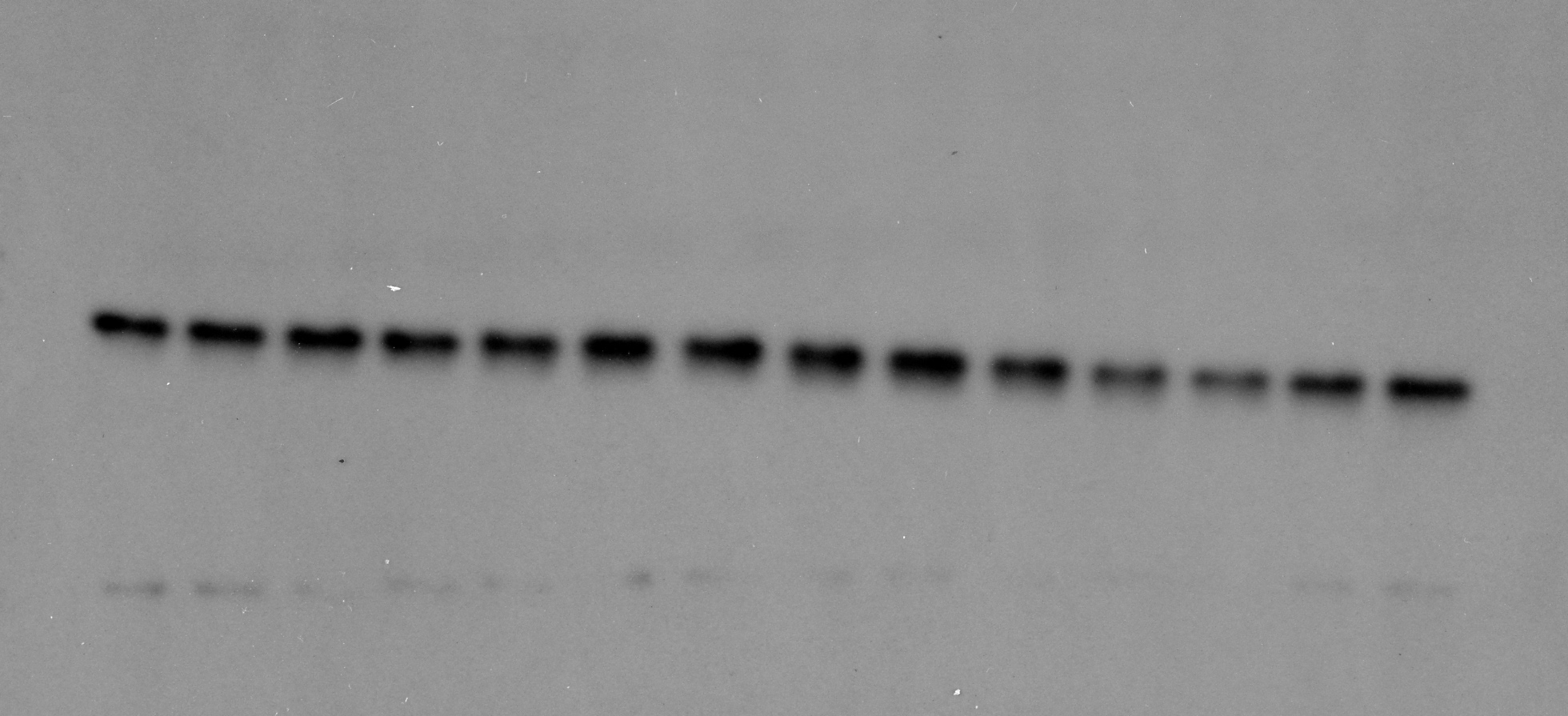

Supplement: Source data 1. [file elife-66582-data1.zip › Blots and Blot Figs/Blots/WT Cyto/WT Cyto p-GSK3b.jpg]

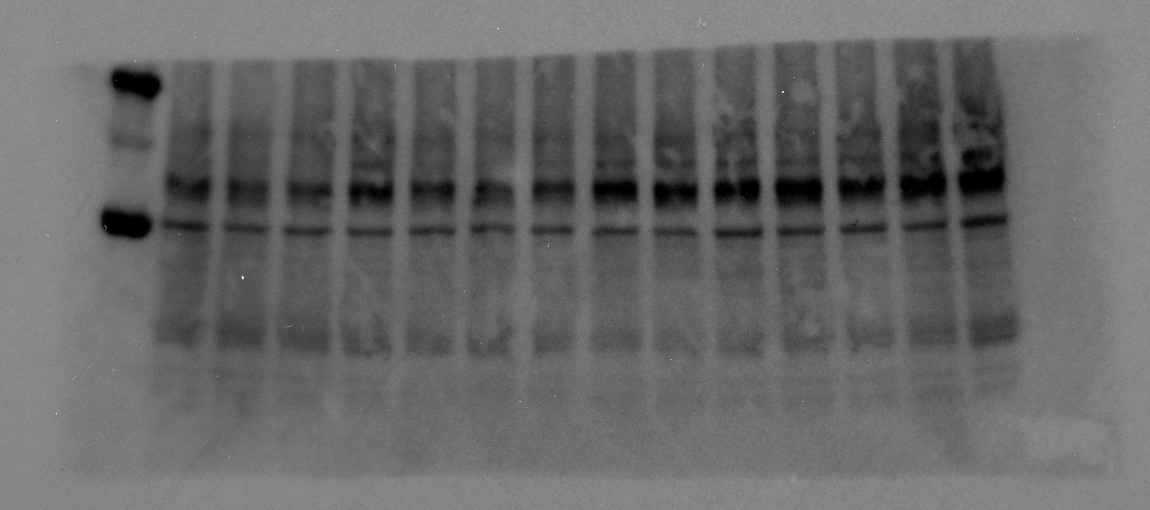

Supplement: Source data 1. [file elife-66582-data1.zip › Blots and Blot Figs/Blots/WT Cyto/WT Cyto p-HDAC4.jpg]

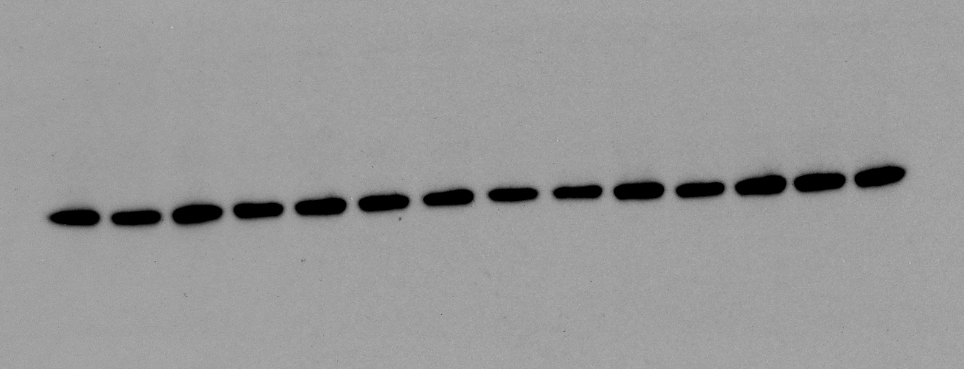

Supplement: Source data 1. [file elife-66582-data1.zip › Blots and Blot Figs/Blots/WT Cyto/WT GAPDH1.jpg]

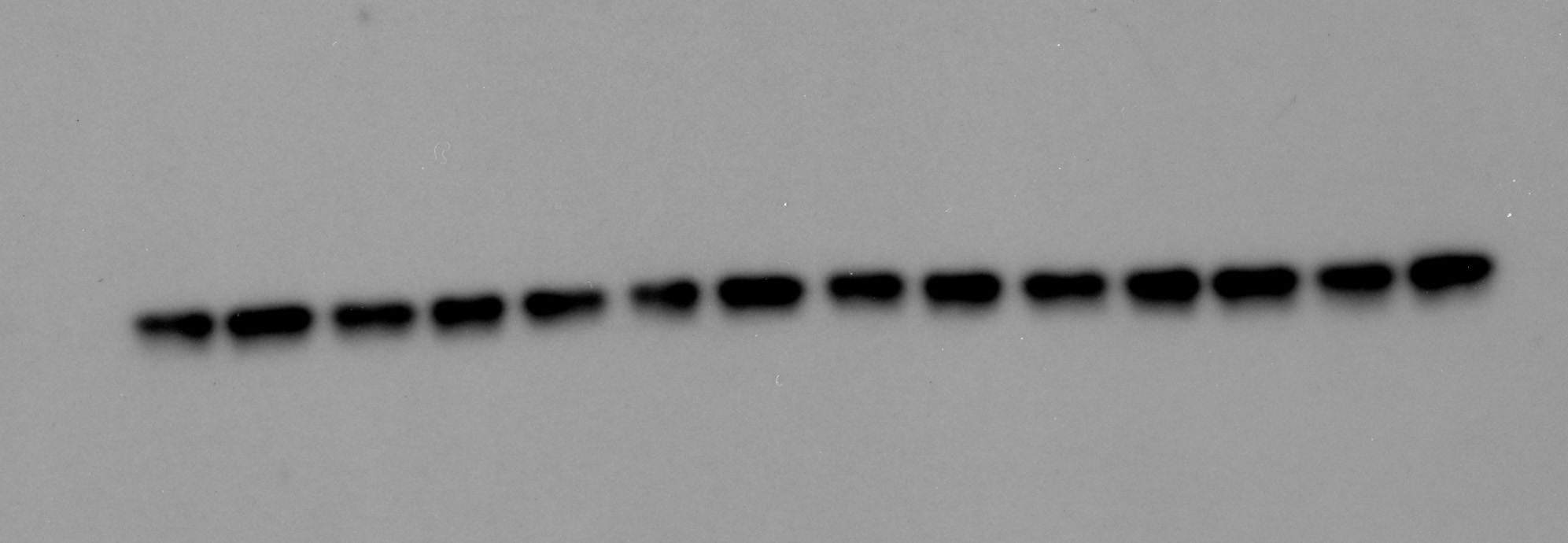

Supplement: Source data 1. [file elife-66582-data1.zip › Blots and Blot Figs/Blots/WT Cyto/WT GAPDH2.jpg]

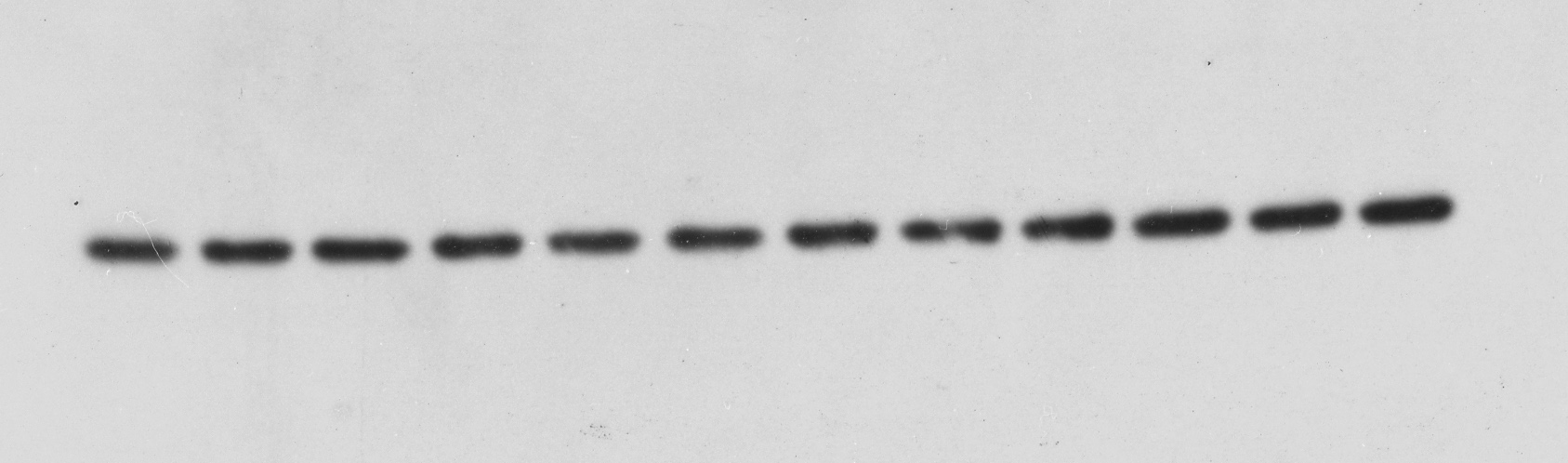

Supplement: Source data 1. [file elife-66582-data1.zip › Blots and Blot Figs/Blots/WT Cyto/WT GAPDH3.jpg]

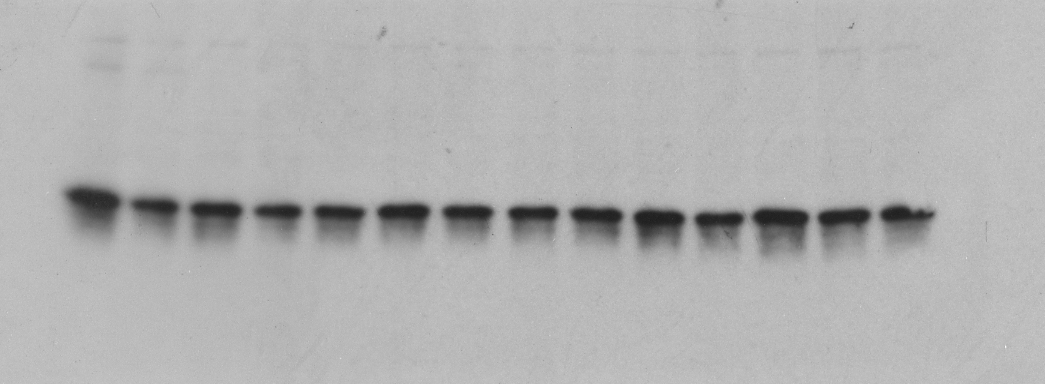

Supplement: Source data 1. [file elife-66582-data1.zip › Blots and Blot Figs/Blots/WT Cyto/WT GAPDH4.jpg]

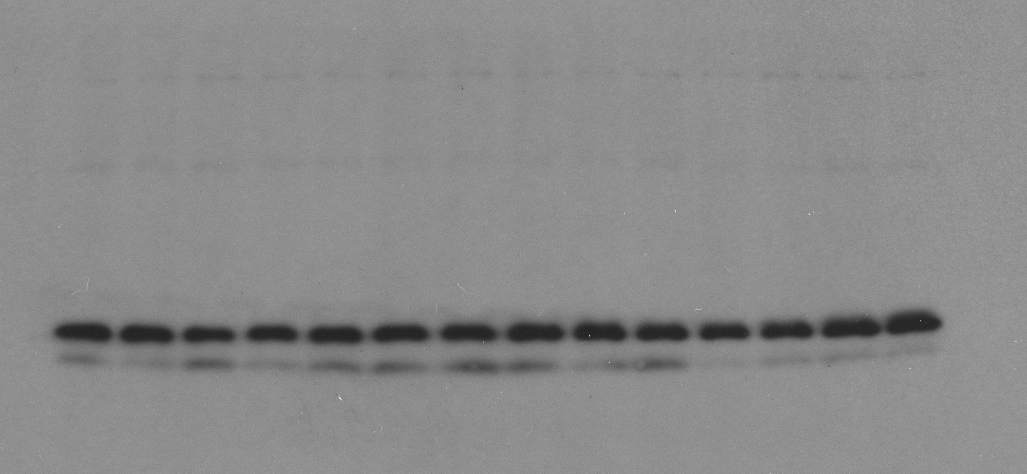

Supplement: Source data 1. [file elife-66582-data1.zip › Blots and Blot Figs/Blots/WT Nu/WT Histone H2B1.jpg]

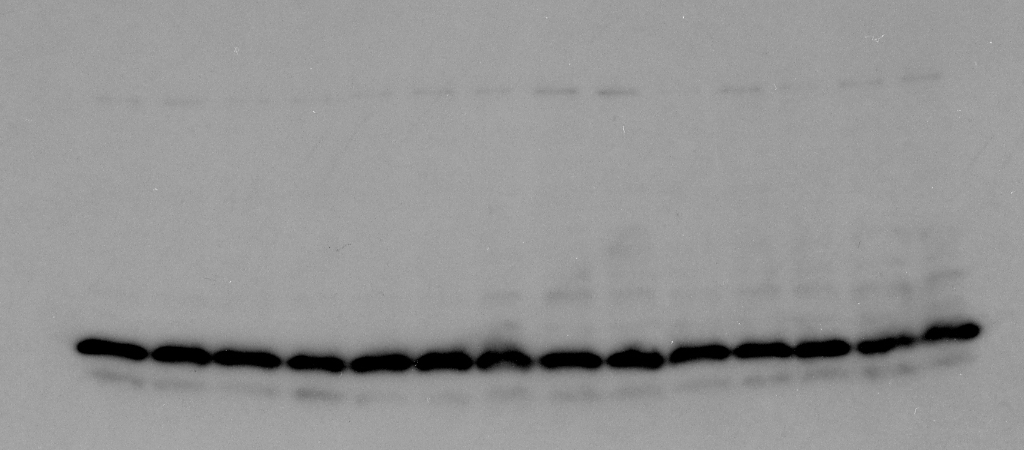

Supplement: Source data 1. [file elife-66582-data1.zip › Blots and Blot Figs/Blots/WT Nu/WT Histone H2B2.jpg]

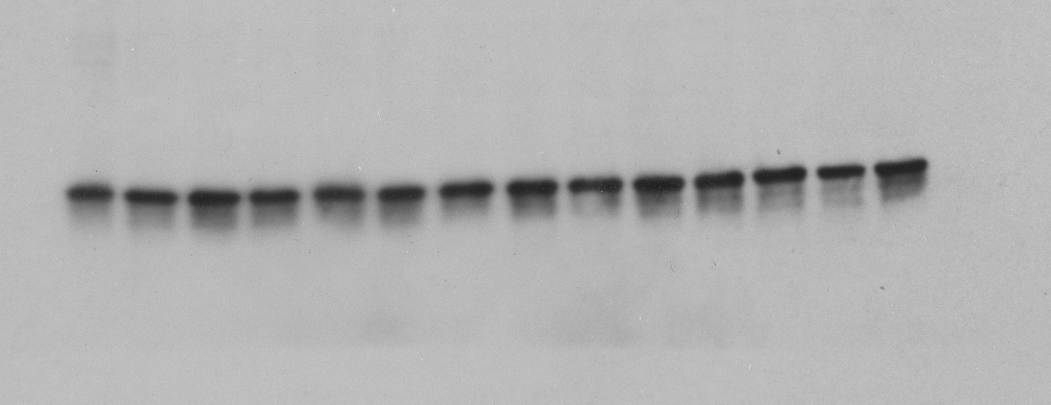

Supplement: Source data 1. [file elife-66582-data1.zip › Blots and Blot Figs/Blots/WT Nu/WT Histone H2B3.jpg]

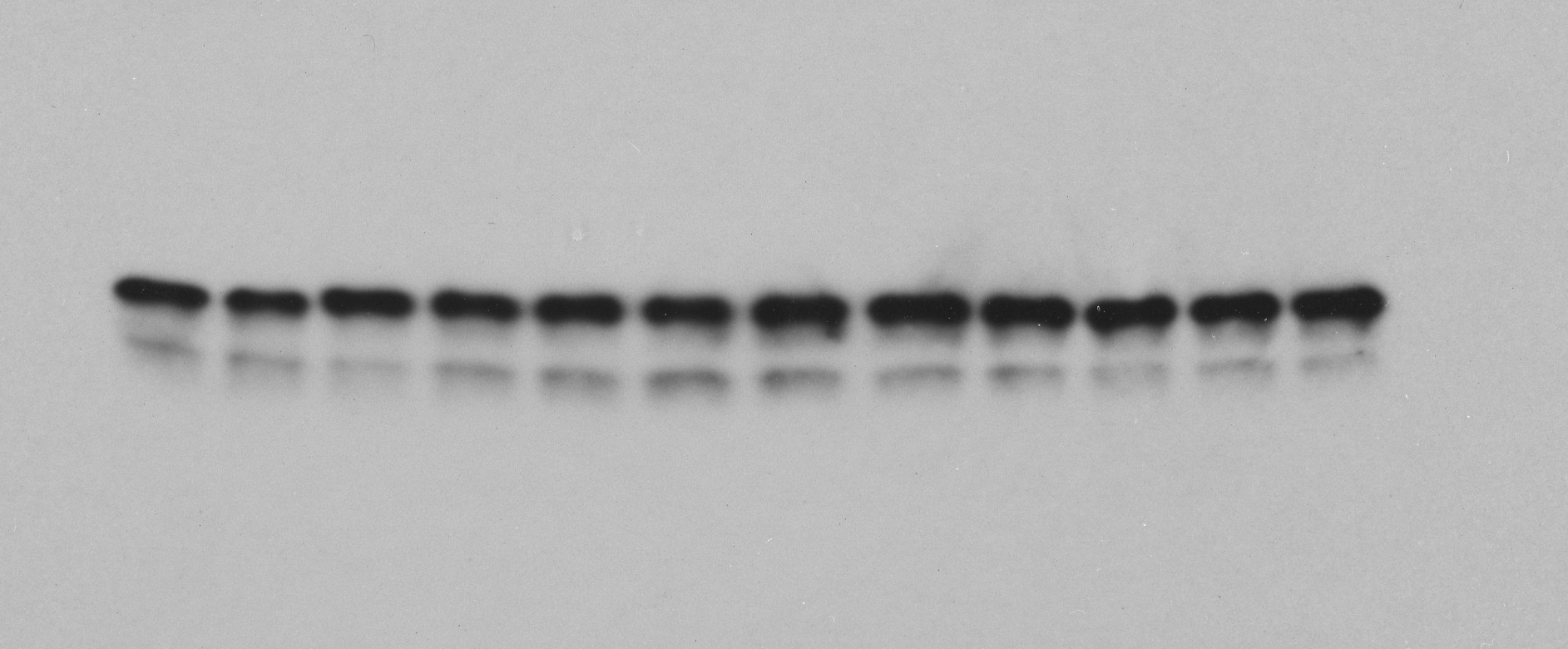

Supplement: Source data 1. [file elife-66582-data1.zip › Blots and Blot Figs/Blots/WT Nu/WT Histone H2B4.jpg]
