# Supplementary material for: The Ca2+-activated cation channel TRPM4 is a positive regulator of pressure overload-induced cardiac hypertrophy
Source: eLife. 2021 Jun 30;10:e66582. doi: 10.7554/eLife.66582 (PMC8245133; doi:10.7554/eLife.66582)
Supplement: Supplementary file 1. — Haemodynamic measurements include heart rate (HR), aortic systolic and diastolic pressure, LV systolic pressure, dP/dtmax and dP/dtmin; anatomical measurements include body weight (BW), heart weight (HW), LV weight (LVW), lung weight (LW), tibial length (TL), heart weight normalised by body weight (HW/BW), LV weight normalised by body weight and tibial length (LVW/BW; LVW/TL), lung weight normalised by body weight (LW/BW). Data are presented as means ± SEM. Comparison between sham and TAC in WT or Trpm4 cKO groups: **p<0.01, ***p<0.001; Comparison between WT and Trpm4 cKO TAC groups: #p<0.05, ###p<0.001. [file elife-66582-supp1.docx]

**Supplementary File 1. Hemodynamic and anatomical parameters after 2 days and 14 days of sham/TAC in WT and *Trpm4* cKO mice.**

|  | **2 days** | | | | **14 days** | | | |
| --- | --- | --- | --- | --- | --- | --- | --- | --- |
|  | WT | | *Trpm4* cKO | | WT | | *Trpm4* cKO | |
|  | Sham | TAC | Sham | TAC | Sham | TAC | Sham | TAC |
| **Hemodynamic parameter** |  |  |  |  |  |  |  |  |
| n |  | | | | 6 | 6 | 6 | 7 |
| HR (bpm) |  |  |  |  | 503.00  ± 2.78 | 505.83  ± 4.27 | 491.17  ± 6.00 | 496.00  ± 10.67 |
| Aortic systolic pressure (mmHg) |  |  |  |  | 103.83  ± 2.27 | 161.00  ± 2.57 *** | 100.83  ± 3.61 | 163.43  ± 2.23 *** |
| Aortic diastolic pressure (mmHg) |  |  |  |  | 73.33  ± 6.31 | 78.67  ± 2.72 | 72.50  ± 1.18 | 77.14  ± 2.72 |
| LV systolic pressure (mmHg) |  |  |  |  | 103.67  ± 3.68 | 162.50  ± 2.86 *** | 106.67  ± 2.86 | 164.43  ± 3.04 *** |
| dP/dt_max_ (mmHg/s) |  |  |  |  | 9403.00 ± 466.66 | 9559.67  ± 703.60 | 9470.67  ± 424.55 | 9199.14  ± 372.03 |
| dP/dt_min_ (mmHg/s) |  |  |  |  | -9492.83 ± 186.90 | -9642.83 ± 681.67 | -9706.33  ± 551.89 | -9924.57 ± 506.24 |
| **Anatomical parameter** |  |  |  |  |  |  |  |  |
| n | 7 | 7 | 8 | 6 | 7 | 7 | 7 | 9 |
| BW (g) | 26.29  ± 0.54 | 26.03  ± 0.80 | 23.75  ± 0.33 | 25.85  ± 0.51 | 26.26  ± 0.67 | 26.60  ± 0.46 | 26.96  ± 0.98 | 27.14  ± 0.69 |
| HW (mg) | 126.57  ± 1.82 | 128.00  ± 4.68 | 116.25  ± 1.77 | 124.50  ± 1.23 | 122.71  ± 3.46 | 163.29  ± 3.79 *** | 127.71  ± 4.15 | 151.22  ± 4.52 ** |
| LVW (mg) | 88.29  ± 1.89 | 92.57  ± 4.78 | 80.25  ± 1.26 | 91.00  ± 1.13 | 88.43  ± 2.87 | 126.71  ± 2.96 *** | 93.00  ± 3.57 | 114.33 ^#^  ± 2.92 *** |
| LW (mg) | 137.44  ± 0.98 | 136.63  ± 2.19 | 130.01  ± 2.06 | 135.07  ± 1.69 | 138.66  ± 2.26 | 147.66  ± 3.65 | 137.94  ± 2.75 | 144.38  ± 2.30 |
| TL (mm) | 17.47  ± 0.10 | 17.49  ± 0.20 | 16.63  ± 0.06 | 17.58  ± 0.15 | 17.37  ± 0.09 | 17.29  ± 0.09 | 17.64  ± 0.16 | 17.66  ± 0.09 |
| HW/BW (mg/g) | 4.82  ± 0.04 | 4.91  ± 0.04 | 4.90  ± 0.05 | 4.83  ± 0.10 | 4.67  ± 0.04 | 6.14  ± 0.09 *** | 4.74  ± 0.03 | 5.57 ^###^  ± 0.09 *** |
| LVW/BW (mg/g) | 3.40  ± 0.03 | 3.55  ± 0.10 | 3.38  ± 0.05 | 3.53  ± 0.08 | 3.36  ± 0.03 | 4.77  ± 0.10 *** | 3.45  ± 0.05 | 4.22 ^###^  ± 0.09 *** |
| LVW/TL (mg/mm) | 5.05  ± 0.09 | 5.28  ± 0.22 | 4.82  ± 0.10 | 5.18  ± 0.07 | 5.09  ± 0.15 | 7.33  ± 0.18 *** | 5.27  ± 0.17 | 6.47 ^###^  ± 0.15 *** |
| LW/BW (mg/g) | 5.24  ± 0.08 | 5.27  ± 0.09 | 5.48  ± 0.07 | 5.24  ± 0.13 | 5.29  ± 0.09 | 5.55  ± 0.12 | 5.15  ± 0.17 | 5.33  ± 0.09 |

Hemodynamic measurements include heart rate (HR), aortic systolic and diastolic pressure, LV systolic pressure, dP/dt_max_ and dP/dt_min_; anatomical measurements include body weight (BW), heart weight (HW), LV weight (LVW), lung weight (LW), tibial length (TL), heart weight normalized by body weight (HW/BW), LV weight normalized by body weight and tibial length (LVW/BW; LVW/TL), lung weight normalized by body weight (LW/BW). Data are presented as means ± SEM. Comparison between sham and TAC in WT or *Trpm4* cKO groups: ***p* < 0.01, ****p* < 0.001; Comparison between WT and *Trpm4* cKO TAC groups: ^#^*p* < 0.05, ^###^*p* < 0.001.
